# Complementary intestinal mucosa and microbiota responses to caloric restriction

**DOI:** 10.1038/s41598-018-29815-7

**Published:** 2018-07-27

**Authors:** Kalina Duszka, Sandrine Ellero-Simatos, Ghim Siong Ow, Marianne Defernez, Eeswari Paramalingam, Adrian Tett, Shi Ying, Jürgen König, Arjan Narbad, Vladimir A. Kuznetsov, Hervé Guillou, Walter Wahli

**Affiliations:** 1 Lee Kong Chian School of Medicine, Nanyang Technological University Singapore, Singapore, 308232 Singapore; 2 Center for Integrative Genomics, University of Lausanne, Lausanne, 1015 Switzerland; 3 Department of Nutritional Sciences, University of Vienna, Vienna, 1090 Austria; 4 Toxalim (Research Centre in Food Toxicology), Université de Toulouse, INRA, ENVT, INP-Purpan, UPS, Toulouse, 31300 France; 5 Bioinformatics Institute, A*STAR Biomedical Sciences Institutes, Singapore, 13867 Singapore; 6 Quadram Institute Bioscience, , Norwich Science Park, Norwich, Norfolk, NR7UA UK; 7 Vienna Metabolomics Center (VIME), University of Vienna, Vienna, 1090 Austria; 8 SUNY Upstate Medical University Syracuse, Syracuse, NY 13210 USA

## Abstract

The intestine is key for nutrient absorption and for interactions between the microbiota and its host. Therefore, the intestinal response to caloric restriction (CR) is thought to be more complex than that of any other organ. Submitting mice to 25% CR during 14 days induced a polarization of duodenum mucosa cell gene expression characterised by upregulation, and downregulation of the metabolic and immune/inflammatory pathways, respectively. The HNF, PPAR, STAT, and IRF families of transcription factors, particularly the *Pparα* and *Isgf3* genes, were identified as potentially critical players in these processes. The impact of CR on metabolic genes in intestinal mucosa was mimicked by inhibition of the mTOR pathway. Furthermore, multiple duodenum and faecal metabolites were altered in CR mice. These changes were dependent on microbiota and their magnitude corresponded to microbial density. Further experiments using mice with depleted gut bacteria and CR-specific microbiota transfer showed that the gene expression polarization observed in the mucosa of CR mice is independent of the microbiota and its metabolites. The holistic interdisciplinary approach that we applied allowed us to characterize various regulatory aspects of the host and microbiota response to CR.

## Introduction

The beneficial effects of caloric restriction (CR) were described as early as the 16th century (Luigi Cornaro, 1484–1566: “Discorsa della vita sobria”) and have been intensively investigated since the 1930s. However, the mechanisms behind the health outcomes of CR are not well understood. Numerous publications have consistently shown that CR increases lifespan and health span in different species regardless of sex, at least when there is little exposure to potentially life-shortening diseases^1–3^. A complex network of pathways and factors including Sirt1, mTOR, AMPK, IGF-1, FoxO, thyroid hormone, and ghrelin contribute to the overall beneficial effects of CR^4^. Most organs share common CR-modulated processes, but also have individual responses. The most prevalent process is the regulation of genes connected with inflammation, protein folding, metabolism, circadian rhythm, tumorigenesis, and detoxification^5,6^.

Surprisingly, the gastrointestinal (GI) tract, which is the first organ in contact with food, has not been well studied in the context of CR, but it is known that CR influences innate and adaptive immunity in the intestine^7,8^. In addition, CR also results in hypertrophy of the stomach^9^, but reduces cell proliferation in the duodenum and colorectal crypt cells and enterocyte differentiation^10–12^. However, there are conflicting results concerning the impact of CR on intestinal epithelial cell apoptosis^11,13^, villi length^14–16^, and colon weight and length^11,15,17^. CR does not influence intestinal permeability^18^, but it does counteract the age-related decline in nutrition absorption by increasing the uptake of proline, fructose, and glucose, without affecting mRNA levels of the glucose transporters^14,15^. Finally, CR increases the bile acid (BA) content in the intestine, which enhances lipid absorption from the intestinal lumen^19^.

The GI tract is singular for its crucial symbiotic interactions with a huge and diverse population of prokaryotic cells, the microbiota, and for harnessing indispensable macro- and micronutrients. Composition changes in the gut microbiota occur during weight gain and loss^20–23^, and the type and amount of food are the main factors shaping the gut microbiota composition^23,24^. An increased ratio of *Firmicutes* to *Bacteroidetes* is a hallmark of obesity^25,26^. Accordingly, CR has a strong influence on gut microbiota^27,28^.

This limited information on the intestinal response to CR prompted us to study the molecular response to CR of the duodenum mucosa and microbiota. We analysed the surface of the mucosa consisting mainly of absorptive enterocytes but also various other types of cells^29^. We also asked if and how the microbiota reacts and contributes to the response to CR. We discovered a clear polarisation of differentially expressed genes, with metabolic genes being upregulated and immune/inflammatory genes downregulated in the duodenum of CR mice. This gene expression dichotomisation revealed tissue-specific transcription markers of CR and their network correlated with essential changes in the faecal microbiota composition and metabolite content.

## Results

### CR induces dichotomisation of the gene expression response in the duodenum mucosa

To explore the effects of CR on duodenum functions, we reduced the food intake of mice by 25% for 14 days (experimental set up is presented in Supplementary Fig. S1), which resulted in ~20% body weight loss (Supplementary Fig. S2A) and a reduction in body fat content (Supplementary Fig. S1B). The length of the small intestine (SI) and colon did not change significantly during CR (Supplementary Fig. S2C,D). However, the ratio of SI and colon length to body mass increased in CR animals, which may reflect an adaptation to increase the efficiency of nutrient uptake (Supplementary Fig. S2E,F).

Microarray-based gene expression profiling analysis of duodenum mucosa scrapings revealed 521 differentially expressed genes (DEGs) (*p*-value cut-off of 0.05; fold-change ≥1.5). A total of 249 genes were significantly upregulated and 272 were downregulated in CR mice compared with *ad libitum* mice (Supplementary Tables S1A,B).

**Figure 1:**
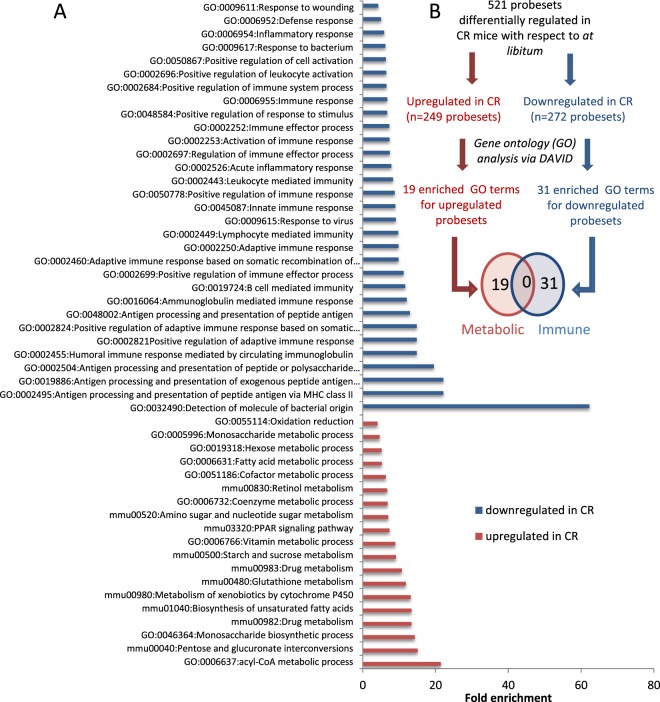
Caloric restriction (CR) induces dichotomisation of two major classes of biological processes in the duodenum mucosa. (**A**) Enriched gene ontology (GO) terms associated with upregulated probe sets or downregulated probe sets (DAVID Bioinformatics, 6.7). (**B**) Analyses performed for differentially expressed probe sets to identify enriched GO terms. Venn diagram of the enriched GO terms identified for the upregulated and downregulated probe sets.

This gene expression response to CR in the duodenum mucosa was assessed by gene ontology (GO) functional enrichment analysis^30^, which defined 19 enriched terms associated with metabolic processes for the upregulated genes (Figs. 1A,B, Supplementary Table S2A) and 31 terms associated with immune and inflammatory responses for the downregulated genes (Fig. 1A,B, Supplementary Table S2B). The lack of any overlapping GO term between the up- and downregulated genes suggested a strong dichotomisation of biological processes for metabolism and immunity. Furthermore, enrichment analysis of tissue-associated proteins carried out using the Uni-Prot datasets (‘UP_Tissue’) (Supplementary Table S2C) showed that the 249 metabolic associated gene subset was represented by proteins referring to small intestine, colon, kidney and liver, while the 272 immune system associated gene subset was represented by the proteins connected to jejunal and colic lymph nodes (at 87.14-fold enrichment), thymus, spleen, activated spleen, and bone marrow. In total, 96 DIGs were categorized as genes related to the immune system and 87.5% of these genes were transcriptionally downregulated. The list of these downregulated genes contains genes serving as specific markers for T-cells (*Il2rg*, *Cd52*, *Atf3* and *Laptm5*), B-cells (*Cd72*, *Cybasc3*, *Parp14*, *Bank1*), NK-lymphocytes (*Nkg7*), murine macrophages (*H2-q6*), monocytes (*Tgm2*, *Nkg7*) and mature dendritic cells (*Cxcl10*, *Ifit1*, *Tnfsf10*, *Atf3 and Usp18)*^31–35^.

In summary, the GO analysis suggested that the functional response of the duodenum mucosa to CR is associated with dichotomised gene expression patterns referring to the immune and metabolic pathways.

MetaCore (MetaCore^TM^; GeneGo Inc.) pathway enrichment analysis of the differentially expressed genes confirmed this dichotomisation (Supplementary Table S3A,B), further demonstrating that CR enhanced metabolic gene expression and reduced immune/inflammatory responses in the mouse duodenum mucosa.

### Enriched interacting partners of the genes affected by CR

Next, using the MetaCore^TM^ gene-connectivity analysis hypergeometric test, we identified 78 genes from the entire mouse genome, which according to the literature could interact with the DEGs in the CR mouse duodenum (Supplementary Table S4A). Each of the 78 genes had a significantly higher number of interactions (literature and manual curation via MetaCore^TM^) with the DEGs, when compared with the background set (i.e. all the genes from the genome), as assessed by the standard hypergeometric test (Supplementary Table S4A). A large number of them (62 out of 78) were transcription factor genes, such as *Stat1/2/3/6*, *Irf1/2/3/7/8/9*, *Pparα*, *Pparγ*, and *Hnf1α/Hnf3α/Hnf3β/Hnf4α*. Other genes encode enzymes such as *p300* and *Oas1* as well as cell membrane receptors such as *Cd36*, *Cd74*, and *Tlr2*. Of interest, the transcription factors *Stat1/2/3/6* and *Irf1/2/3/7/8/9* significantly interacted with genes downregulated under CR (Supplementary Table S4C) but not with those that were upregulated (Supplementary Table S4B). Also notable is that *Stat1*, *Stat2*, *Irf1*, and *Irf9* were among the significantly downregulated genes in the CR mouse duodenum. In contrast, the transcription factor genes *Pparα*, *Pparγ*, and *Hnf1α/Hnf3α/Hnf3β/Hnf4α* significantly interacted with the upregulated probe sets, although only *Pparα* mRNA was significantly upregulated in the CR duodenum (Supplementary Fig. S3A). Overall, of the 57 genes that interacted with the downregulated genes, 16 (28%) were also significantly downregulated after CR, suggesting a potential tight self-regulating interconnected gene network. Furthermore, the limited overlap between the genes that interacted with upregulated vs. downregulated genes confirmed the dichotomic control of metabolic and inflammatory/immune response to CR (Supplementary Fig. S3B).

### Interconnected networks of genes affected by CR

**Figure 2:**
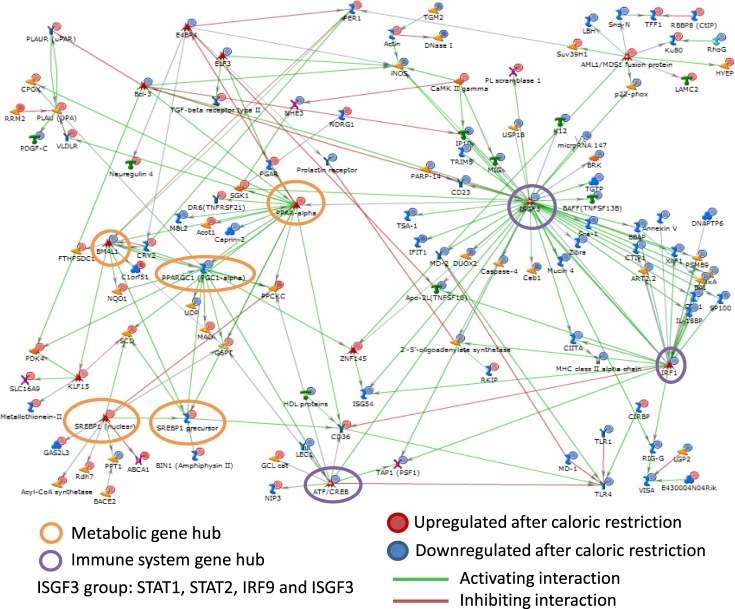
MetaCore^TM^ analysis of gene interactions. The MetaCore^TM^ gene network of the genes differentially regulated after caloric restriction. The genes were represented by Affymetrix probe sets, which were selected based on an adjusted *p-*value threshold of 0.05.

Mapping of the 521 genes differentially expressed in CR yielded 493 MetaCore^TM^ identified genes, which were subsequently used for network building. The MetaCore^TM^ network revealed a very high connectivity between genes (Supplementary Table S5). A large directly interconnected network was comprised of 119 genes (Fig. 2) with several gene hubs, such as the ISGF3 (interferon-stimulated gene factor) group comprising *Stat1*, *Stat2*, *Irf9*, and *Irf1*, and the *Pparα* group comprising *Bmal1*, *Ppargc1* (*Pgc1α*), *Srebp1* (nuclear), *Spebp1* precursor, *Atf-3*, and *Evi-1*. In type 1 interferon signalling, the Stat1–Stat2 heterodimer combines with IRF9 (interferon response factor) to form a complex known as ISGF3^36^. In fact, the most connected hub was the ISGF3 immune system gene hub, with 47, 20, 13, and 5 interactions for *Stat1*, *Stat2*, *Irf9*, and *Isgf3*, respectively. Most of the genes in this hub were downregulated after CR. The next important gene hub was the *Pparα* metabolic gene hub. The PPARα-interacting genes were generally upregulated after CR. The *Pparα* hub interacted with the *Bmal1*, *Ppargc1* (*Pgc1α*), and *Srebp1* hubs, which are all associated with energy metabolism. Overall, this network analysis confirmed the stimulatory and inhibitory impact of CR on the metabolic and immune systems, respectively. In addition, it revealed that PPARα and ISGF3 are likely the key transcription factors mediating this regulation.

**Figure 3:**
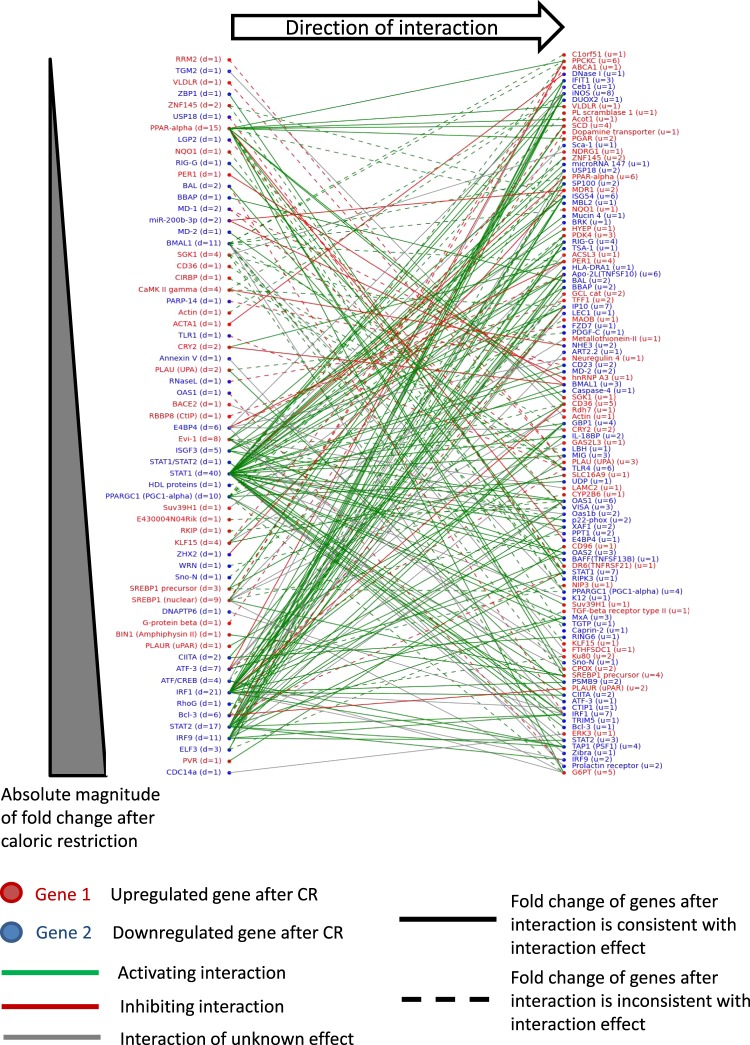
Bipartite graph of the genes differentially regulated after caloric restriction in the wild-type mice. The genes were represented by Affymetrix probe sets, which were selected based on an adjusted *p*-value threshold of 0.05. The numbers of downstream targets and upstream regulators for each of the nodes are denoted by the variables d and u, respectively.

Next, we plotted a bipartite graph of the direct interaction network (Fig. 3) to look for specific patterns of direct regulation with respect to activating or inhibiting interactions with genes exhibiting either small fold- or large fold-changes. The genes that we proposed above to be critical in the control of immune system processes, including *Stat2*, *Irf9*, and *Irf1*, were among those with the largest absolute fold-changes, potentially with a larger impact on downstream targets via transcriptional control.

### Different parts of the GI tract show individual responses to CR

**Figure 4:**
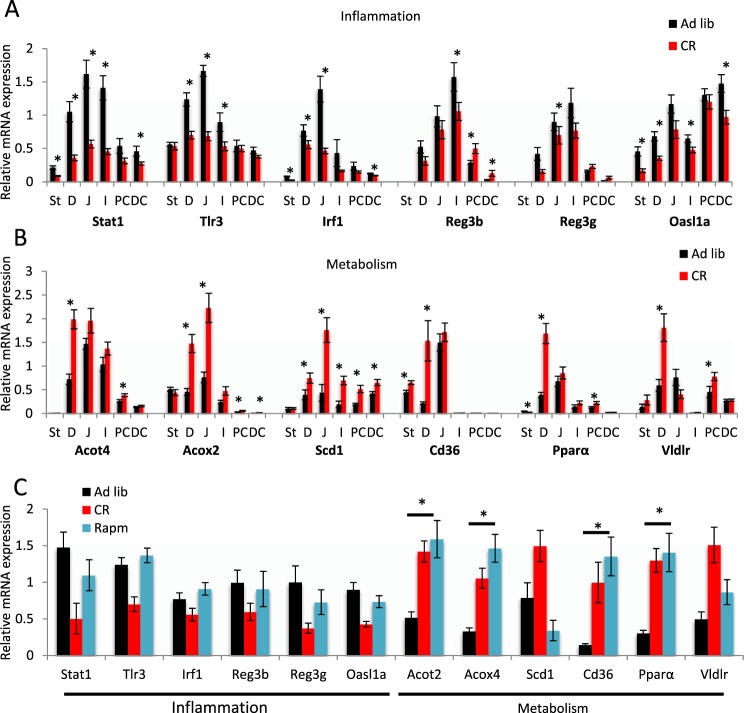
Caloric restriction influences inflammatory and metabolic gene expression changes along the gastrointestinal (GI) tract. The relative mRNA levels of representative inflammatory (**A**) and metabolic (**B**) genes were measured in six tissues along the GI tract. Two-tailed Student’s t-tests were used to assess statistical significance; n = 8–10, *p < 0.05. Gene expression changes were also assayed in the duodenum epithelium of mice receiving rapamycin (Rapm) in their drinking water (**C**). One-way ANOVA and the Bonferroni post-hoc test were used to compare the experimental groups; n = 7–9. Data are presented as the mean ± SEM. St-stomach, D-duodenum, J-jejunum, I-ileum, PC proximal colon, DC distal colon.

To characterise the response of the entire GI tract to CR, we performed comparative qPCR analysis of several DEGs identified by microarray analysis in the duodenum mucosa, using samples from the stomach, jejunum, ileum, and proximal and distal colon. Duodenum samples were also included for qPCR-based validation of the microarray-based observations (Fig. 4). The groups of genes tested were connected with the immune/inflammatory response (*Stat1*, *Tlr3*, *Irf1*, *Reg3b*, *Reg3g*, *Oasl1a*; Fig. 4A) and metabolism (*Acot4*, *Acox2*, *Scd1*, *Cd36*, *Pparα*, *Vldlr*; Fig. 4B). Among genes connected with immunity/inflammation (*Stat1*, *Tlr3*, *Irf1*), there was a significant downregulation when duodenum samples from *ad libitum* mice were compared to those from CR mice, confirming the microarray results. Strong responses were also observed in other parts of the SI (jejunum, ileum) compared with the stomach and colon, where they were much weaker. In addition, antimicrobial peptide (*Reg3b*, *Reg3g*, *Oasl1a*) gene expression was reduced during CR in all parts of the GI tract with the exception of the colon, which responded to CR by increasing transcription of *Reg3b* (Fig. 4A).

Genes connected with metabolism showed higher expression in the duodenum compared with colon and stomach in *ad libitum* fed mice. However, in response to CR, the expression of most of these genes increased not only in the duodenum, but also in the colon and stomach (Fig. 4B). Transcription of *Pparα* increased, in line with a previous report^37^, and network analysis revealed this gene was an essential regulator of the CR response (Fig. 2) and confirmed that a short 14 day CR is sufficient to induce a strong CR-like phenotype. Of interest, a simple overnight fast resulted in statistically significant CR-like gene expression changes for *Stat1*, *Tlr3*, *Acot2*, and *Acox4* (Supplementary Fig. S3C). Altogether, these results show that gene expression in the SI differs in amplitude from that of the stomach and colon under *ad libitum* and CR conditions.

### Response to CR in duodenum mucosa differs from that of other organs

We took advantage of published comparisons of commonly regulated genes after CR for several organs^38^, including the hypothalamus, liver, muscle, and colon to assess the potential specificity of the duodenum CR response. In contrast to the immune, inflammatory, and defence processes that were mostly downregulated in the duodenum, DNA packaging, nucleosome and chromatin assembly, and the biosynthesis of cholesterol, lipids, steroids, and sterol were the most downregulated processes in the colon, liver, muscle, and hypothalamus (Supplementary Fig. S4A). Duodenum shared a very limited number (14 upregulated and 5 downregulated) of CR-regulated genes with the colon, liver, muscle, and hypothalamus (Supplementary Fig. S4B,C) showing a strong tissue specific gene expression response to CR. These findings were confirmed by comparison with other recent data^39^. There was minimum or no overlap between microarray DEGs identified in different tissues (skeletal muscles, white adipose tissue, heart and brain neocortex)^39^ with those identified in duodenum mucosa samples in this study (Supplementary Figure S4D–H). These observations show that the duodenum mucosa presents a singular CR gene expression signature, most likely reflecting unique interactions among the duodenum, microbiota and food.

### The mTOR pathway regulates metabolic gene expression in a CR-like manner

The most strongly upregulated (8.5 fold change, p ≤ 0.001) gene in the CR duodenum gene expression microarray data set was *Fkbp5* (Supplementary Table S1A). FKBP5 forms a complex with rapamycin and acts as an inhibitor of mTOR^40,41^. This finding prompted us to investigate whether rapamycin would induce a duodenum CR phenotype. It did so partially by stimulating the metabolism-associated genes. However, it did not affect immunity/inflammation genes, including those encoding antibacterial peptides (Fig. 4C). Additionally, similar to CR-exposed mice, mice receiving rapamycin had lower body weight than the control mice (Supplementary Fig. S4I). We concluded that the mTOR pathway regulates the expression of metabolic genes in a CR-like manner.

### CR-triggered metabolic and inflammatory gene regulation is not microbiota-dependent

**Figure 5:**
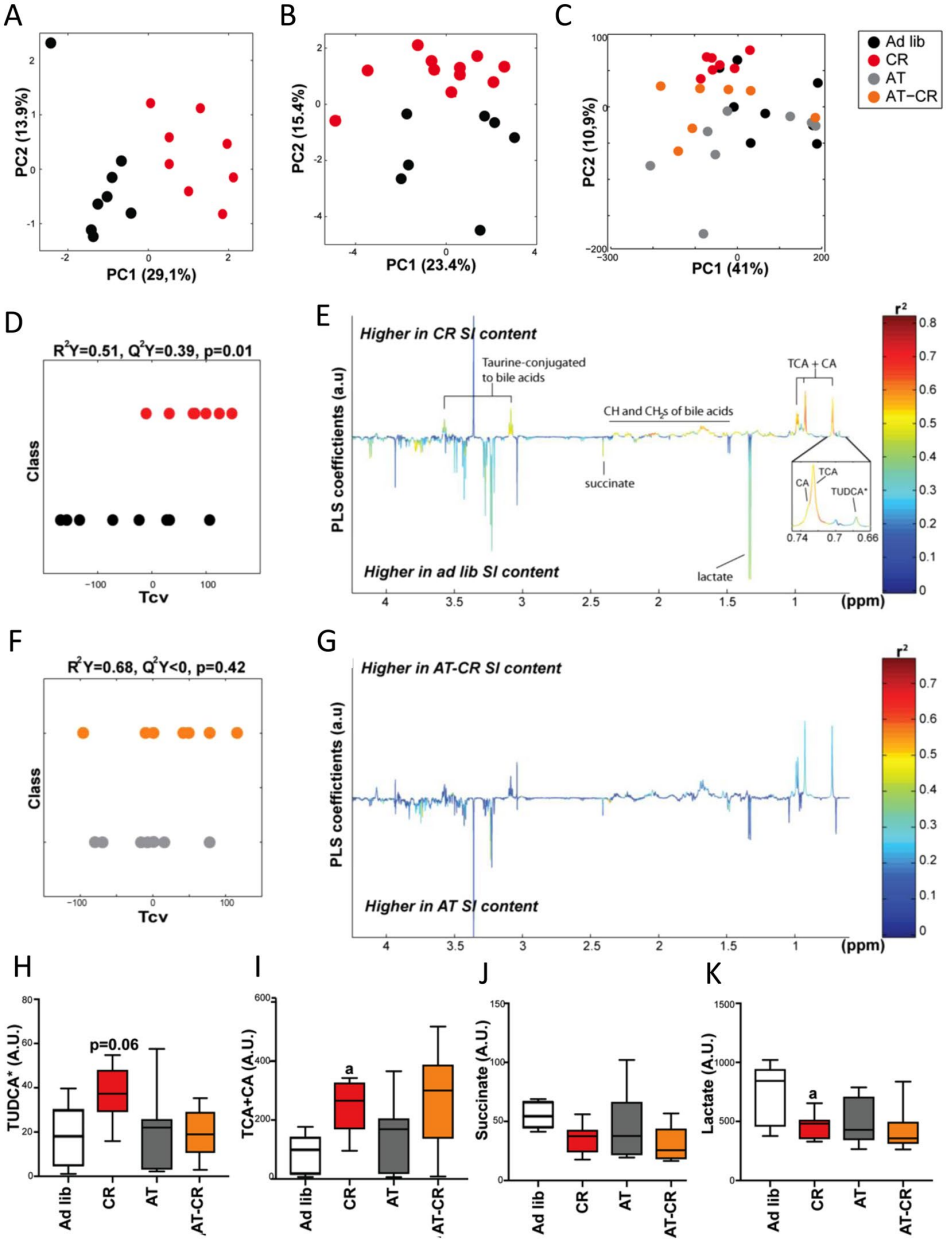
Caloric restriction (CR) results in significant changes in the duodenum bacteria composition and metabolite content. Principal component analysis was performed on 16 S rRNA sequences from faecal (**A**) and duodenum microbiota (**B**) and the duodenum content ^1^H-NMR metabolic profiles (**C**). The projection on latent structure-discriminant analysis (PLS-DA) cross-validated scores (**D**,**F**) and loadings (**E**,**G**) for the discrimination between ^1^H-NMR spectra from duodenum extracts from CR vs. *ad libitum* (Ad lib) mice (**D**,**E**) and AT vs AT-CR mice (**F**,**G**). Q^2^Y represents the goodness of fit for the PLS-DA models and *p-*values were derived using 1000 permutations of the Y matrix. Metabolites are color-coded according to their correlation coefficient. Metabolites pointing upward and with R^2^ > 0.49 were significantly increased and those pointing downwards were decreased in the faeces from CR mice. The area under the curve of the 1H-NMR spectra was integrated for TUDCA (H), TCA + CA (**I**), succinate (**J**), and lactate (**K**). Data are presented as a 10–90 percentile boxplot with mean. Groups were compared using one-way ANOVA and Sidak’s multiple comparison post-tests. a: *p* < 0.05 for CR compared with Ad lib.

Homeostasis in the intestinal tissue is orchestrated by microbiota cues that are mediated by Toll-like receptors (TLRs)^42^. In addition to this regulation, we found CR modifies the expression of antimicrobial peptides all along the GI tract. Taken together, these results suggest crosstalk between the intestine and the microbiota during CR. To address this question, we analysed the microbiota in both the feces and small intestine. In the feces, at the phyla level, the ratio of *Firmicutes* to *Bacteroidetes* was not drastically altered by CR, but increased diversity was observed within the families that constitute each of these phyla (Fig. 5A,B, Supplementary Figs S5 and S6). *Firmicutes Clostridia Clostridiales* were less dominant under CR, to the benefit of other families such as *Firmicutes Bacillii* (*Lactobacillaceae*), *Clostridia* (*Lachnospiraceae*, *Ruminococcaceae*) and *Erysipelotrichi* (*Erysipelotrichaceae*). Similarly, the *Bacteroidetes Bacteroida S24-7* was less dominant to the benefit of *Bacteroidetes Bacteroida* (*Bacteroidaceae*, *Porphyromonadaceae*, and *Prevotellaceae*). In the small intestine, 25% of the OTUs were unassigned under *ad libitum* conditions and 9% under CR. Under CR, there was a decrease in *Actinobacteria* and *Proteobacteria* relative to both *Firmicutes* and *Bacteroidetes*. Moreover, the *Firmicutes* were dominated by *Lactobacillaceae*, whilst a more balanced spectrum (e.g. with *Lachnospiraceae*, as well as *Ruminococcaceae*) was observed in the *ad libitum* samples. *S24-7* was the only assigned family for *Bacteroidetes* (Supplementary Figs S5 and S6). We observed a stronger impact of CR on SI bacteria than faecal bacteria. We then asked whether the microbiota has a significant role in the duodenum dichotomisation of gene expression described above. We tested this hypothesis using mice treated with antibiotics (AT) to deplete their gut flora, and then subjected them to CR. Successful depletion of microbiota in AT mice was confirmed by (i) DAPI staining of bacteria in faecal samples (Supplementary Table S6), (ii) the enlarged cecum of the treated mice, and (iii) the lack of short-chain fatty acids (SCFAs) in the CR faeces (Supplementary Fig. S7A–D); while, microbiota transfer reverted the cecum phenotype (Supplementary Fig. S7A).

Among the subsets of inflammation- and metabolism-related genes that we tested, the CR-induced gene expression changes in the duodenum mucosa were similar between AT animals and animals with a regular microbiota (Supplementary Fig. S7E). Furthermore, duodenum gene expression profiles did not differ between *ad libitum* mice gavaged with faecal or duodenum microbiota from CR (MT-F, MT-D) mice and from control *ad libitum*–fed mice (MT-FC, MT-DC; Supplementary Fig. S7E). We concluded that the duodenum mucosa regulation of metabolic and inflammatory genes triggered by CR in the duodenum mucosa is not primarily microbiota-dependent.

### CR induces changes in intestinal and faecal metabolites

To characterise the metabolic consequences of CR, we analysed duodenum content (Supplementary Table S7A) and faecal samples (Supplementary Table S7B) using ^1^H-NMR–based metabolomics. Examples of the typical metabolic profiles obtained are displayed in Supplementary Fig. S8A,B. CR induced significant changes in duodenum metabolite content, as illustrated by the significant differences between the *ad libitum* and CR metabolic profiles using projection on latent structure-discriminant analysis (PLS-DA) modelling (Fig. 5C–E). Microbiota depletion attenuated the effect of CR, with a non-significant PLS-DA model between AT and AT-CR animals (Fig. 5C,F,G). In the duodenum, CR selectively increased many signals related to BA moieties, such as taurine-conjugated to BAs (triplets at 3.08 and 3.57 ppm) and CH- and -CH_2_ groups of BAs (multiplets between 1.2 and 2.2 ppm) (Fig. 5E). CR also increased the signals of two singlets at 0.726 and 0.73 ppm, corresponding to the C-18, C-19, and C-21 methyl groups of BAs. These signals were unambiguously assigned to cholic (CA) and taurocholic (TCA) acids by comparison with spikes in experiments of standard molecules (Supplementary Fig. S8C,D). Three other BA signals were detected in the NMR spectra of duodenum content, which were assigned to ß-muricholic acid (ßMCA), tauro-ß-muricholic acid (TßMCA), and tauroursodeoxycholic (TUDCA) acids based on previous publications^43^. Levels of TUDCA tended to increase upon CR (Fig. 5E,H); while, those of ßMCA, and TßMCA remained unchanged (Supplementary Fig. S8E). Accordingly, CR resulted in increased levels of ß-hydroxybutyrate in plasma (Supplementary Fig. S8E). Importantly, antibiotic treatment prevented the rise of CA, TCA, and TUDCA levels in the duodenum of AT-CR mice (Fig. 5H,I). Additionally, removal of bacteria resulted in increases in ßMCA and TßMCA levels independently of CR (Supplementary Fig. S8E). CR also decreased succinate and lactate levels in the duodenum content (Fig. 5J,K). This regulation was absent in the small intestine of CR mice with deprived microbiota.

**Figure 6:**
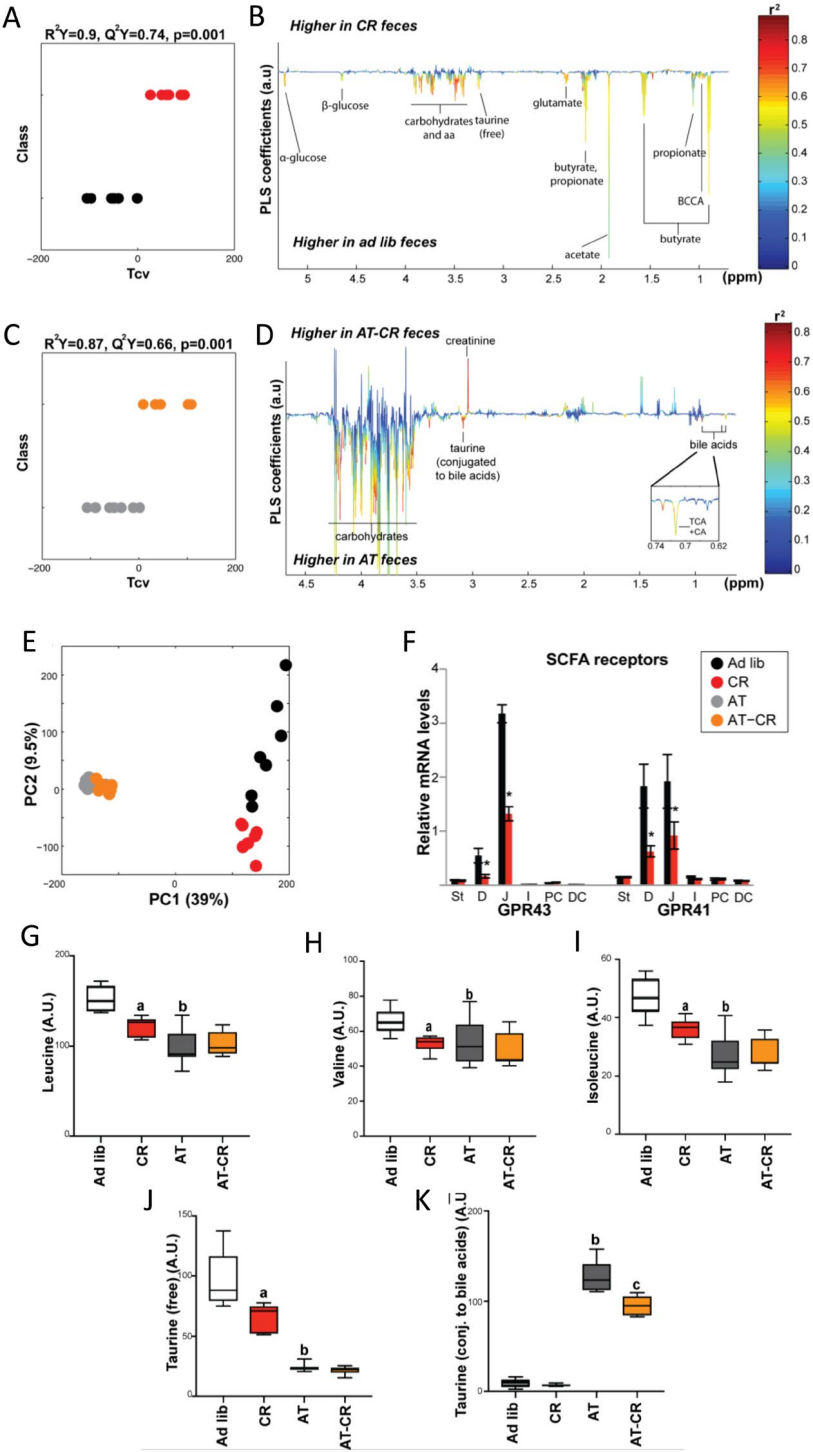
Caloric restriction (CR) results in significant changes in faecal bacteria composition and metabolite content. Projection on latent structure-discriminant analysis (PLS-DA) cross-validated scores (**A**,**C**) and loadings (**B**,**D**) for the discrimination between ^1^H-NMR spectra from duodenum extracts from CR vs. *ad libitum* (Ad lib) mice (**A**,**C**) and AT vs AT-CR mice (**B**,**D**). Q^2^Y represents the goodness of fit for the PLS-DA models and *p-*values were derived using 1000 permutations of the Y matrix. Metabolites are color-coded according to their correlation coefficient. Metabolites pointing upward and with R^2^ > 0.49 were significantly increased and those pointing downwards were decreased in the faeces from CR mice. Principal component analysis was performed on faecal ^1^H-NMR metabolic profiles (**E**). mRNA relative expression for short chain fatty acid (SCFA) receptors along the intestine (**F**). St-stomach, D-duodenum, J-jejunum, I-ileum, PC proximal colon, DC distal colon. The area under the curve of the ^1^H-NMR spectra was integrated for leucine (**G**), valine (**H**), isoleucine (**I**), free taurine (**J**), and conjugated taurine (**K**). Data are presented as a 10–90 percentile boxplot with mean. Groups were compared using one-way ANOVA and Sidak’s multiple comparison post-tests. a: *p* < 0.05 CR compared with Ad lib, b: *p* < 0.05 AT compared with Ad lib, c: *p* < 0.05 AT-CR compared with AT.

The PLS-DA analyses conducted on faecal metabolite profiles confirmed that CR induced strong metabolite composition changes in both control and AT-treated mice (Fig. 6A–E). The analysis revealed antibiotic treatment had a very strong effect and there was significant discrimination between *ad libitum* and CR animals (Fig. 6C,E). CR affected SCFAs with decreased acetate, butyrate, and propionate levels (Fig. 6B, Supplementary Fig. S7B–D). Of interest, the CR-induced reduction in SCFAs was concomitant with a decrease in SCFA receptor expression in the duodenum and jejunum, but not in the colon (Fig. 6F). Faecal levels of many amino acids (branched-chain amino acids [BCAAs] - leucine, isoleucine, valine, as well as glutamine and phenylalanine), glucose and other oligosaccharides, and taurine (free) decreased under CR (Figs. 6B,G–J, Supplementary Fig. S8G,H). Most of these changes were gut microbiota-dependant since the levels of SCFAs, amino acids, and taurine were not affected by CR in AT-treated mice (Fig. 6G–I, and Supplementary Fig. S8G). Interestingly, contrary to faecal samples, the BCAA plasma concentrations increased during CR (Supplementary Fig. S8I), in line with a previously published report^44^. In mice lacking microbiota, CR decreased the levels of all NMR-detectable BA signals, as well as the BA-conjugated taurine signals (Fig. 6K, Supplementary Fig. S8J,K). In summary, CR induced significant changes in both duodenum and faecal metabolites, with a strong impact on BAs in the duodenum and on SCFAs and BCAAs in the faeces.

## Discussion

To the best of our knowledge, this is the first report to present gene expression profiling of the SI mucosa after CR. The intestine showed a tissue-specific response to CR by regulating groups of genes with selected functions. In addition, the holistic approach applied here identified a microbiota-dependent response to CR. We found that, under CR, the duodenum mucosa increased metabolism-associated gene transcription, while strongly reducing immune/inflammatory gene expression. This expression patterns most likely reflects the impact of CR on the activation of metabolic processes in the epithelial cells and possibly a reduction of the number of lymphocytes (not studied herein) or/and the suppression of immune cell activity in the duodenum mucosa.

In the intestinal mucosa, 96 immune tissue associated genes responded to CR. Among them were genes associated with T-, B- and NK-lymphocytes, which play pivotal roles in the cellular and humoral immune responses^31–35^. In the activating interaction sub-network, the genes connected with the immunity/inflammatory response were concentrated around the *Isgf3* and *Irf1* hubs; while, the enhanced metabolic genes were associated with *Pparα*, *Pgc1α*, *Srebp1*, and *Bmal1*. In CR, *Bmal1* and other circadian clock genes are also affected in other tissues^38^. In fact, *Bmal1* plays role in circadian rhythm as well as in metabolism. Thus, changes in its expression could reflect the affected metabolism of the host under caloric deficit. However, it may also indicate changes in the food intake pattern due to the fact that the CR mice received their daily food allowance as one meal, which they consumed rapidly. Time-restricted food intake has previously been shown to affect clock gene transcription^45^. Importantly, the gut microbiota itself affects the circadian rhythm of gene expression^42^. Disruption of the intestinal circadian clock was reported to change the uptake of water, sugars, proteins, lipids and drugs, as well as gut motility, gut serotonin and cortisol production, with an impact on the whole body^42,46–52^. In addition, PPARα and STAT1 inhibit each other at the transcriptional level^53^, which is consistent with our data. The upregulation of *Pparα*, coupled with the downregulation of *Stat1*, *Stat2*, *Irf1*, and *Irf9* are the likely drivers of the dichotomic gene transcription observed under CR. Our bioinformatics analysis supports the hypothesis that the PPAR, STAT, IRF, and HNF families of transcription factors play critical roles in CR-induced transcriptional regulation.

To gain an overview of transcriptional regulation in response to CR, we compared our gene expression data with published microarray data sets from several organs after CR^5,39^. Although regulation of several immunity/inflammatory and metabolic genes has also been observed in various tissues in response to CR, the number of genes affected was much lower than found here in the duodenum mucosa. Therefore, the duodenum response is clearly distinct from that of other organs. Although decreased transcription of inflammatory genes triggered by CR is not the most prominent response to CR in organs other than the intestine mucosa, it constitutes one of the most common responses to CR among all tissues. It suggests that under CR, the whole organism saves energy by reducing its immune/inflammatory vigilance. Undernutrition, a severe form of CR, and infectious diseases caused by microorganisms have always been intricately linked, and the former is the primary cause of immunodeficiency worldwide^54^. Intestinal response most likely contributes to the outcome of malnutrition.

**Figure 7:**
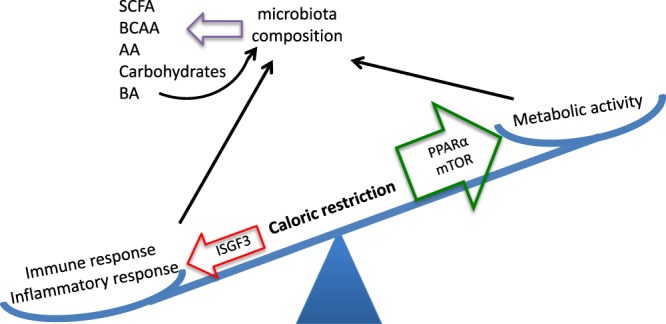
A model of the impact of caloric restriction (CR) on the duodenum. Two important functions coexist and are well balanced in the intestinal mucosa: control of metabolism and the immune/inflammatory response. Various stressors can impact this balance. Intestinal CR leads to decreased expression of immune/inflammatory response genes, which is mediated via ISGF3, and elevated expression of metabolic and antioxidant genes, activated by PPARα and mTOR. The changes in gene expression affect the microbiota composition and microbial metabolite production. BCAA-branch chain amino acids, AA-amino acids, BA-bile acid.

Gut microbiota–host interactions are based on mutual benefits, and several bacterial metabolites serve as signalling molecules, for instance, in shaping host immunity or in appetite regulation and energy homeostasis. It is now well established that microbiota-generated metabolites are implicated in health and disease^55^. An important finding of the current study in mice with depleted gut flora was that the CR-triggered changes in the expression of metabolic and immune/inflammatory genes were not significantly influenced by microbiota. However, it is likely that the changes in microbiota and their metabolites in CR are induced by the host, at least partly. Therefore, we anticipate that the strong polarisation of gene expression under CR and increased efficiency of nutrient uptake by the host would influence the microbiota composition (Fig. 7), as seen in this study for CR and previously found for restricted nutrient uptake^28^. This outcome is reflected by the more abundant and partially different faecal and duodenum metabolites in the *ad libitum* condition compared with CR.

The CR decreases BCAA in faeces, which was not observed in CR mice treated with antibiotics, implying that the CR-induced changes in BCAA almost fully depend on gut microbiota metabolism. In the colon, amino acids are intensively metabolised by microbiota, with lysine, glycine, and arginine BCAAs being the preferred substrates^56^. The BCAAs, as well as other AAs, that were affected by CR such as phenylalanine and glutamate, can be biosynthesized by bacteria and this process contributes significantly to the host AA pool^57^. Of interest, leucine, isoleucine, valine, and phenylalanine, which were upregulated in the faeces of *ad libitum* mice, compared with CR mice, are plasma markers that correlate with an increased diabetes risk^58^. However, it is unclear if the reduced levels of these amino acids contribute to the beneficial health effects of CR.

We found decreased concentrations of all SCFAs in the faeces of CR mice, an effect that was dependent on the microbiota. Of note, significantly higher concentrations of SCFAs were found in obese versus normal-weight children and reduced consumption of carbohydrates by obese subjects resulted in decreased concentrations of butyrate and butyrate-producing bacteria in faeces^59,60^. Butyrate and propionate stimulate intestinal gluconeogenesis by activating gluconeogenic gene expression^61–63^. Thus, shortage of butyrate and propionate during CR, or after antibiotic treatment, may impact the glucose balance in the whole body and enhance the energy deficit during CR. Moreover, decreased butyrate and propionate levels may play a signalling role in CR by preventing stimulation of adipocyte proliferation^64^ and attenuating signalling of satiety^65,66^.

A substantial part of our metabolic profiling results concerns changes in BA levels in the duodenum and faeces, which may directly impact lipid, glucose, and energy metabolism^67,68^. CR induced an increase of TUDCA, CA, and TCA in the SI; while, other BA-related signals remained unchanged. These observations are in line with previous reports demonstrating that CR dose-dependently increased the amount of tauro-conjugated but not unconjugated BAs. Moreover, CR selectively increased 12α-hydroxylated-BAs (i.e. BAs of the CA pathway) and decreased non-12α-hydroxylated groups (i.e. BAs from the MCA pathway) in all compartments of the enterohepatic cycle in mice^19,69^. Interestingly, this increased ratio of 12α-hydroxylated BAs/non-12α-hydroxylated BAs correlated with improved glucose tolerance and lipid parameters^19^ and is suspected to play a key role in metabolic improvement after bypass surgery in humans^70^. Furthermore, impaired generation of 12α-hydroxylated BAs has been associated with dyslipidaemia and insulin resistance^71^. However, these results are controversial since eliminating 12α-hydroxylated BAs in mice through *Cyp8b1* knockout caused low body weight and improved glucose tolerance^72^. Additionally, antidiabetic treatment decreased the 12α-hydroxylated BA/non-12α-hydroxylated BA plasmatic ratio in humans^73^. Therefore, it remains unclear whether the CR-induced BA changes observed in our study mediate the beneficial effects of CR.

The herein described increase in total BAs in plasma and the SI and the increase in TCA and TDCA in the serum of CR mice were previously associated with CR^19,69^, but this is the first report in the context of a microbiota analysis. We demonstrated that the gut microbiota plays a key role in both the CR-induced increase in the amount of tauro-conjugated BAs and in the CR-induced changes in the relative proportions of BAs, as microbiota depletion disrupted the BA levels observed after CR. Moreover, the levels of conjugated vs. free BAs, which is dependent on the presence of microbiota, mirrored the well-established role of gut microbiota in BA conjugation^74,75^. We observed increased levels of TßMCA in antibiotic-treated mice. This corresponds with results of a previous comparative study in germ-free vs. conventional mice, in which TßMCA was suggested to regulate FXR activity^76^. Importantly, secondary BA production by microbiota is accompanied by a feedback mechanism of BAs on microbiota composition^77,78^. Thus, BA concentration changes during CR may reciprocally contribute to the observed microbiota composition changes. In general, the differences in metabolite composition between antibiotic-treated vs. non-treated mice, as well as between *ad libitum* and CR groups, were more pronounced in the faeces compared with the duodenum contents. Thus, the magnitude of the changes corresponds to bacteria abundance in duodenum and colon^79^, indicating a role for gut bacteria in the metabolic response to CR.

It is important to point out that our CR experimental procedure does not include dietary supplementation that would prevent malnutrition, which is a common practice^80^. Importantly, the chow fed during CR provides more than the minimal requirements of micronutrients for mice (Nutrient Requirements of Laboratory Animals)^81^. Therefore, a 25% reduction of the diet should not result in deficiency of amino acids, minerals, or vitamins. Thus, we propose that the short duration and relatively mild diet restriction applied in our study would not result in micronutrient malnutrition.

Gene expression profiling analysis of the duodenum mucosa showed that its response to CR was a strong downregulation of immunity/inflammatory genes and an upregulation of metabolic genes in the duodenum mucosa cells. Shulzhenko and colleagues^82^ reported that elevated inflammation in the intestine leads to impaired lipid absorption. These results combined with ours suggest a finely adjusted balance between mucosal cell metabolism and immune/inflammatory response to optimise energy management and homeostasis during CR. Moreover, the microbiota in the intestine and colon represents an essential source of metabolites, which can serve as nutrients or signalling molecules contributing to the adaptation of the host to the CR metabolic challenge. Further studies are needed to directly identify gene expression profiles of the individual cells, distinct cell types and their integrative response to CR in duodenum. Such studies would provide a better understanding of the metabolic and immune system pathways affected by CR including the roles of the gut microbiota and its metabolites in regulating the host’s physiology and pathophysiology during CR.

## Materials and Methods

### Animal care and experimental procedures

Male C56Bl/6 mice from in-house breed were kept under a 12-h light/12-h dark cycle in standard housing cages with corncob bedding (BioCob, Singapore). The animals were fed Diet 3436 from Irradiated Rat and Mouse Diet (Speciality Feeds, Glen Forrest, Australia) and housed with free water access. Mice aged 12 weeks were randomly divided into experimental groups of 8–10 mice, as follows: Ad lib – control *ad libitum* fed; CR – caloric restriction; AT – antibiotic treated; AT-CR – antibiotic treated and caloric restriction; MT-FC – *ad libitum* fed, antibiotic treated with microbiota transplant from the faeces of Ad lib mice; MT-F – *ad libitum* fed, antibiotic treated with microbiota transplant from the faeces of CR mice; MT-DC – *ad libitum* fed, antibiotic treated with microbiota transplant from the duodenum of Ad lib mice; MT-D – *ad libitum* fed, antibiotic treated with microbiota transplant from the duodenum of CR mice; Rapm – *ad libitum* fed, with rapamycin in drinking water. The groups did not differ significantly in body weight when starting the experimental procedures. This experimental setup is depicted in Suppl Fig. 1.

Animal food intake was measured for one week prior to the intervention to determine the amount of chow diet to be given under CR. The mice from the CR and AT-CR groups underwent 14 days of CR that consisted of an ~75% reduction of daily food intake. To deplete gut flora, mice from the AT, AT-CR, MT-C, and MT groups were repeatedly gavaged with 200 µl of an antibiotic cocktail (vancomycin 0.5 g/l, neomycin 1 g/l, ampicillin 1 g/l, metronidazole 1 g/l; all from Sigma-Aldrich, St. Louis, MO, USA). The AT and AT-CR groups were gavaged three times within 14 days of the experimental procedure, and the MT group was gavaged twice: 5 and 3 days before microbiota transplant. Microbiota depletion in the AT and AT-CR groups was confirmed by faecal sample sequencing.

After gut flora depletion, the mice from the MT groups were gavaged twice at a 2-day interval with freshly extracted duodenum or faecal microbiota from CR or Ad lib mice. To obtain inoculants, the duodenum content was squeezed out and mixed with 1 ml of sterile phosphate-buffered saline. Similarly, fresh faeces were mixed with PBS. The mixture was vortexed and centrifuged for 3 min at 1000 × g, and the isolated supernatant was immediately gavaged into MT mice. Mice were killed 14 days after the first gavage.

Mice in the Rapm group received rapamycin in drinking water for 14 days at a concentration that yielded an intake of 1.5 mg/kg body weight/day of rapamycin.

More information is available in the Supplementary materials and methods.

All animal experimentation protocols were approved by the Vaud Cantonal Authority (authorisation VD 2440), Switzerland, and by the Institutional Animal Care and Use Committee (authorisation 2015/SHS/1023) in Singapore. All experiments were carried out according to Swiss and Singaporean animal experimentation guidelines.

### Microarray

Total RNA from mice duodenum scrapings was isolated and purified using the RNeasy Mini Kit (Qiagen, Venlo, Netherlands). The total RNA was measured with a NanoDrop®ND-1000 spectrophotometer, and the RNA quality was assessed using RNA 6000 NanoChips with the Agilent 2100 Bioanalyzer (Agilent, Palo Alto, CA, USA). For each sample, 100 ng of total RNA was amplified using the Ambion® WT Expression Kit (#4411973, Life Technologies). A total of 5.5 μg of the cDNA was fragmented and labelled using the GeneChip® WT Terminal Labeling kit (901525, Affymetrix). Affymetrix mouse gene 1.0 ST arrays (Affymetrix, Santa Clara, CA, USA) were hybridised with 2.3 μg of fragmented target at 45 °C for 16 h and washed and stained according to the protocol described in the Affymetrix GeneChip® Expression Analysis Manual (Fluidics protocol FS450_0007).

### Sequencing the 16 S rDNA genes and metataxonomic analysis

Bacterial DNA was extracted and sequenced; the results were analysed as described previously^83^. In brief, DNA was extracted using a FastDNA Spin Kit for Soil (MP Biomedicals, UK). Following PCR amplification of the V4 and V5 regions of the 16 S rDNA genes, DNA sequencing was performed using the high-throughput GS FLX Titanium 454 pyrosequencing platform (the GS FLX Titanium platform)^84^. Analyses of the sequencing reads were performed using the Quantitative Insights Into Microbial Ecology (QIIME) pipeline. The resulting taxonomic data were analysed by principal component analysis (PCA).

### ^1^H NMR metabolomics

Faecal and duodenum extract preparations, NMR spectroscopy, and data pre-treatment were conducted as previously described^83^. Data were mean-centred and scaled using unit variance scaling prior to analysis using orthogonal projection on latent structure-discriminant analysis (O-PLS-DA). The ^1^H NMR data were used as independent variables (X matrix) and regressed against a dummy matrix (Y matrix) indicating the class of samples (*ad libitum* or CR)^85^. The O-PLS–derived model was evaluated for goodness of prediction (Q^2^Y value) using 8-fold cross-validation. The reliability of each model was established using a permutation test of the Y vector (1000 permutations) to determine a *p-*value for each Q^2^Y, as previously described^86^. Parameters of the final models are indicated in the figure legends.

To identify metabolites discriminating the animal groups, the O-PLS-DA correlation coefficients (r^2^) were calculated for each variable and back-scaled into a spectral domain, so that the shape of the NMR spectra and the sign of the coefficients were preserved^87^. The weights of the variables were color-coded according to the square of the O-PLS-DA correlation coefficients. Correlation coefficients extracted from significant models were filtered so that only significant correlations above the threshold defined by Pearson’s critical correlation coefficient (*p* < 0.05; |r| > 0.49) were considered significant. For illustration purposes, the area under the curve of several signals of interest was integrated, and statistical significance was tested using t-tests or one-way ANOVA. More information is provided in the Supplementary materials and methods. All authors had access to the study data and reviewed and approved the final manuscript.

### Data availability

The microarray datasets generated during and analyzed during the current study are available in the ArrayExpress repository, https://www.ebi.ac.uk/arrayexpress/experiments/E-MTAB-6248. The sequencing results are deposited in the NCBI data base.

## Electronic supplementary material

Supplementary tables

Supplementary figures

Supplementary information

## References

[1] Masoro EJ (2000). Caloric restriction and aging: an update. Exp Gerontol.

[2] Weindruch R, Sohal RS (1997). Seminars in medicine of the Beth Israel Deaconess Medical Center. Caloric intake and aging. N Engl J Med.

[3] Ayres JS, Schneider DS (2009). The role of anorexia in resistance and tolerance to infections in Drosophila. PLoS Biol.

[4] Speakman JR, Mitchell SE (2011). Caloric restriction. Molecular aspects of medicine.

[5] Swindell WR (2008). Comparative analysis of microarray data identifies common responses to caloric restriction among mouse tissues. Mech Ageing Dev.

[6] Swindell WR (2009). Genes and gene expression modules associated with caloric restriction and aging in the laboratory mouse. BMC Genomics.

[7] Lara-Padilla E (2011). Caloric restriction reduces IgA levels and modifies cytokine mRNA expression in mouse small intestine. The Journal of nutritional biochemistry.

[8] Suarez-Souto MA (2012). Caloric restriction modifies both innate and adaptive immunity in the mouse small intestine. Journal of physiology and biochemistry.

[9] Yang H, Youm YH, Nakata C, Dixit VD (2007). Chronic caloric restriction induces forestomach hypertrophy with enhanced ghrelin levels during aging. Peptides.

[10] Lok E (1988). Dietary restriction, cell proliferation and carcinogenesis: a preliminary study. Cancer letters.

[11] Yilmaz OH (2012). mTORC1 in the Paneth cell niche couples intestinal stem-cell function to calorie intake. Nature.

[12] Koga A, Kimura S (1980). Influence of restricted diet on the cell cycle in the crypt of mouse small intestine. Journal of nutritional science and vitaminology.

[13] Holt PR, Moss SF, Heydari AR, Richardson A (1998). Diet restriction increases apoptosis in the gut of aging rats. *The journals of gerontology*. Series A, Biological sciences and medical sciences.

[14] Casirola DM, Lan Y, Ferraris RP (1997). Effects of changes in calorie intake on intestinal nutrient uptake and transporter mRNA levels in aged mice. *The journals of gerontology*. Series A, Biological sciences and medical sciences.

[15] Casirola DM, Rifkin B, Tsai W, Ferraris RP (1996). Adaptations of intestinal nutrient transport to chronic caloric restriction in mice. The American journal of physiology.

[16] Zhang JG (2012). Food restriction alters villi morphology in obese rats: gut mechanism for weight regain?. Experimental biology and medicine.

[17] Ferraris RP, Cao QX, Prabhakaram S (2001). Chronic but not acute energy restriction increases intestinal nutrient transport in mice. The Journal of nutrition.

[18] Ma TY, Hollander D, Dadufalza V, Krugliak P (1992). Effect of aging and caloric restriction on intestinal permeability. Experimental gerontology.

[19] Fu ZD, Klaassen CD (2013). Increased bile acids in enterohepatic circulation by short-term calorie restriction in male mice. Toxicology and applied pharmacology.

[20] Cani PD (2007). Selective increases of bifidobacteria in gut microflora improve high-fat-diet-induced diabetes in mice through a mechanism associated with endotoxaemia. Diabetologia.

[21] Murphy EF (2010). Composition and energy harvesting capacity of the gut microbiota: relationship to diet, obesity and time in mouse models. Gut.

[22] Turnbaugh PJ, Backhed F, Fulton L, Gordon JI (2008). Diet-induced obesity is linked to marked but reversible alterations in the mouse distal gut microbiome. Cell host & microbe.

[23] Zhang C (2010). Interactions between gut microbiota, host genetics and diet relevant to development of metabolic syndromes in mice. The ISME journal.

[24] van Hylckama Vlieg JE, Veiga P, Zhang C, Derrien M, Zhao L (2011). Impact of microbial transformation of food on health - from fermented foods to fermentation in the gastro-intestinal tract. Current opinion in biotechnology.

[25] Armougom F, Raoult D (2008). Use of pyrosequencing and DNA barcodes to monitor variations in Firmicutes and Bacteroidetes communities in the gut microbiota of obese humans. BMC genomics.

[26] Ley RE (2005). Obesity alters gut microbial ecology. Proceedings of the National Academy of Sciences of the United States of America.

[27] Santacruz A (2009). Interplay between weight loss and gut microbiota composition in overweight adolescents. Obesity (Silver Spring).

[28] Zhang C (2013). Structural modulation of gut microbiota in life-long calorie-restricted mice. Nat Commun.

[29] Evans EM, Burdett K (1973). The use of isolated cells to assess the contribution of the mucosal epithelium to the metabolism of the intestinal wall. Gut.

[30] Huang da W, Sherman BT, Lempicki RA (2009). Systematic and integrative analysis of large gene lists using DAVID bioinformatics resources. Nat Protoc.

[31] Glowacka WK, Alberts P, Ouchida R, Wang JY, Rotin D (2012). LAPTM5 protein is a positive regulator of proinflammatory signaling pathways in macrophages. J Biol Chem.

[32] Lind A (2012). The immune cell composition in Barrett’s metaplastic tissue resembles that in normal duodenal tissue. PLoS One.

[33] Lyons YA, Wu SY, Overwijk WW, Baggerly KA, Sood AK (2017). Immune cell profiling in cancer: molecular approaches to cell-specific identification. NPJ Precis Oncol.

[34] Ouchida R (2008). A lysosomal protein negatively regulates surface T cell antigen receptor expression by promoting CD3zeta-chain degradation. Immunity.

[35] Pedersen G (2015). Development, validation and implementation of an *in vitro* model for the study of metabolic and immune function in normal and inflamed human colonic epithelium. Dan Med J.

[36] Qureshi SA, Salditt-Georgieff M, Darnell JE (1995). Tyrosine-phosphorylated Stat1 and Stat2 plus a 48-kDa protein all contact DNA in forming interferon-stimulated-gene factor 3. Proceedings of the National Academy of Sciences of the United States of America.

[37] Sung B, Park S, Yu BP, Chung HY (2004). Modulation of PPAR in aging, inflammation, and calorie restriction. The journals of gerontology. Series A, Biological sciences and medical sciences.

[38] Selman C (2006). Coordinated multitissue transcriptional and plasma metabonomic profiles following acute caloric restriction in mice. Physiol Genomics.

[39] Barger JL (2017). Identification of tissue-specific transcriptional markers of caloric restriction in the mouse and their use to evaluate caloric restriction mimetics. Aging Cell.

[40] Baughman G, Wiederrecht GJ, Campbell NF, Martin MM, Bourgeois S (1995). FKBP51, a novel T-cell-specific immunophilin capable of calcineurin inhibition. Molecular and cellular biology.

[41] Hausch F, Kozany C, Theodoropoulou M, Fabian AK (2013). FKBPs and the Akt/mTOR pathway. Cell Cycle.

[42] Mukherji A, Kobiita A, Ye T, Chambon P (2013). Homeostasis in intestinal epithelium is orchestrated by the circadian clock and microbiota cues transduced by TLRs. Cell.

[43] Martin FP (2007). A top-down systems biology view of microbiome-mammalian metabolic interactions in a mouse model. Mol Syst Biol.

[44] Valerio A, D’Antona G, Nisoli E (2011). Branched-chain amino acids, mitochondrial biogenesis, and healthspan: an evolutionary perspective. Aging.

[45] Balakrishnan A, Stearns AT, Ashley SW, Tavakkolizadeh A, Rhoads DB (2010). Restricted feeding phase shifts clock gene and sodium glucose cotransporter 1 (SGLT1) expression in rats. The Journal of nutrition.

[46] Stearns AT, Balakrishnan A, Rhoads DB, Ashley SW, Tavakkolizadeh A (2008). Diurnal rhythmicity in the transcription of jejunal drug transporters. J Pharmacol Sci.

[47] Pan X, Terada T, Irie M, Saito H, Inui K (2002). Diurnal rhythm of H+-peptide cotransporter in rat small intestine. American journal of physiology. Gastrointestinal and liver physiology.

[48] Pan X, Hussain MM (2009). Clock is important for food and circadian regulation of macronutrient absorption in mice. J Lipid Res.

[49] Furuya S, Yugari Y (1974). Daily rhythmic change of L-histidine and glucose absorptions in rat small intestine *in vivo*. Biochim Biophys Acta.

[50] Fisher RB, Gardner ML (1976). A diurnal rhythm in the absorption of glucose and water by isolated rat small intestine. J Physiol.

[51] Ebert-Zavos E, Horvat-Gordon M, Taylor A, Bartell PA (2013). Biological clocks in the duodenum and the diurnal regulation of duodenal and plasma serotonin. PloS one.

[52] Balakrishnan A (2008). Diurnal rhythmicity in glucose uptake is mediated by temporal periodicity in the expression of the sodium-glucose cotransporter (SGLT1). Surgery.

[53] Lee JH, Joe EH, Jou I (2005). PPAR-alpha activators suppress STAT1 inflammatory signaling in lipopolysaccharide-activated rat glia. Neuroreport.

[54] Katona P, Katona-Apte J (2008). The interaction between nutrition and infection. Clin Infect Dis.

[55] Lee WJ, Hase K (2014). Gut microbiota-generated metabolites in animal health and disease. Nature chemical biology.

[56] Macfarlane S, Macfarlane GT (2003). Regulation of short-chain fatty acid production. The Proceedings of the Nutrition Society.

[57] Sridharan GV (2014). Prediction and quantification of bioactive microbiota metabolites in the mouse gut. Nature communications.

[58] Wang TJ (2011). Metabolite profiles and the risk of developing diabetes. Nature medicine.

[59] Payne AN (2011). The metabolic activity of gut microbiota in obese children is increased compared with normal-weight children and exhibits more exhaustive substrate utilization. Nutrition & diabetes.

[60] Duncan SH (2007). Reduced dietary intake of carbohydrates by obese subjects results in decreased concentrations of butyrate and butyrate-producing bacteria in feces. Appl Environ Microbiol.

[61] Gautier-Stein A, Zitoun C, Lalli E, Mithieux G, Rajas F (2006). Transcriptional regulation of the glucose-6-phosphatase gene by cAMP/vasoactive intestinal peptide in the intestine. Role of HNF4alpha, CREM, HNF1alpha, and C/EBPalpha. J Biol Chem.

[62] De Vadder F (2014). Microbiota-generated metabolites promote metabolic benefits via gut-brain neural circuits. Cell.

[63] De Vadder F, Plessier F, Gautier-Stein A, Mithieux G (2015). Vasoactive intestinal peptide is a local mediator in a gut-brain neural axis activating intestinal gluconeogenesis. Neurogastroenterol Motil.

[64] Li G, Yao W, Jiang H (2014). Short-chain fatty acids enhance adipocyte differentiation in the stromal vascular fraction of porcine adipose tissue. The Journal of nutrition.

[65] Chambers ES (2015). Effects of targeted delivery of propionate to the human colon on appetite regulation, body weight maintenance and adiposity in overweight adults. Gut.

[66] Psichas A (2015). The short chain fatty acid propionate stimulates GLP-1 and PYY secretion via free fatty acid receptor 2 in rodents. Int J Obes (Lond).

[67] Nguyen A, Bouscarel B (2008). Bile acids and signal transduction: role in glucose homeostasis. Cellular signalling.

[68] Wahlstrom A, Sayin SI, Marschall HU, Backhed F (2016). Intestinal Crosstalk between Bile Acids and Microbiota and Its Impact on Host Metabolism. Cell Metab.

[69] Fu ZD, Cui JY, Klaassen CD (2015). The Role of Sirt1 in Bile Acid Regulation during Calorie Restriction in Mice. PLoS One.

[70] Dutia R (2015). Temporal changes in bile acid levels and 12alpha-hydroxylation after Roux-en-Y gastric bypass surgery in type 2 diabetes. Int J Obes (Lond).

[71] Haeusler RA, Astiarraga B, Camastra S, Accili D, Ferrannini E (2013). Human insulin resistance is associated with increased plasma levels of 12alpha-hydroxylated bile acids. Diabetes.

[72] Bertaggia E (2017). Cyp8b1 ablation prevents Western diet-induced weight gain and hepatic steatosis because of impaired fat absorption. Am J Physiol Endocrinol Metab.

[73] Gu Y (2017). Analyses of gut microbiota and plasma bile acids enable stratification of patients for antidiabetic treatment. Nat Commun.

[74] Gilliland SE, Speck ML (1977). Deconjugation of bile acids by intestinal lactobacilli. Appl Environ Microbiol.

[75] Archer, R. H., Chong, R. & Maddox, I.S. Hydrolysis of bile acid conjugates by Clostridium bifermentans. *Applied Microbiology and Biotechnology* **14** (1982).

[76] Sayin SI (2013). Gut microbiota regulates bile acid metabolism by reducing the levels of tauro-beta-muricholic acid, a naturally occurring FXR antagonist. Cell Metab.

[77] Begley M, Gahan CG, Hill C (2005). The interaction between bacteria and bile. FEMS Microbiol Rev.

[78] Buffie CG (2015). Precision microbiome reconstitution restores bile acid mediated resistance to Clostridium difficile. Nature.

[79] Sender R, Fuchs S, Milo R (2016). Revised Estimates for the Number of Human and Bacteria Cells in the Body. PLoS Biol.

[80] Cerqueira FM, Kowaltowski AJ (2010). Commonly adopted caloric restriction protocols often involve malnutrition. Ageing Res Rev.

[81] *National Research Council (US) Subcommittee on Laboratory Animal Nutrition. Nutrient Requirements of Laboratory Animals: Fourth Revised Edition*, 1995 (1995). 25121259

[82] Shulzhenko N (2011). Crosstalk between B lymphocytes, microbiota and the intestinal epithelium governs immunity versus metabolism in the gut. Nature medicine.

[83] Duszka K (2016). Intestinal PPARgamma signalling is required for sympathetic nervous system activation in response to caloric restriction. Sci Rep.

[84] Ellis RJ (2013). Comparison of the distal gut microbiota from people and animals in Africa. PloS one.

[85] Trygg J, Wold S (2002). Orthogonal projections to latent structures (O-PLS). J Chemometr.

[86] Eriksson L, Trygg J, Wold S (2008). CV-ANOVA for significance testing of PLS and OPLS (R) models. J Chemometr.

[87] Cloarec O (2005). Statistical total correlation spectroscopy: an exploratory approach for latent biomarker identification from metabolic 1H NMR data sets. Anal Chem.

