# Supplementary material for: Complementary intestinal mucosa and microbiota responses to caloric restriction
Source: Sci Rep. 2018 Jul 27;8:11338. doi: 10.1038/s41598-018-29815-7 (PMC6063912; doi:10.1038/s41598-018-29815-7)
Supplement: Supplementary file 1 — Supplementary tables [file 41598_2018_29815_MOESM1_ESM.docx]

**Supplementary Tables 1-7**

Supplementary Table 1: The 521 DEGs. **(A)** 249 significantly upregulated DEGs **(B)** 272 downregulated DEGs in the duodenum mucosa samples of caloric restriction vs. *ad libitum* control mice, detected by microarray-based gene expression profiling and selected by the adjusted *p*-value <0.05 at |FC|>1.5. Probe sets are ordered by individual adjusted *p*-values.

**(A)** Up-regulated DEGs

| **Probsets ID** | **Gene Symbol** | **Gene Description** | **mRNA ID** | **adj. *P*-value** | **Fold Change** |
| --- | --- | --- | --- | --- | --- |
| 10433241 | Dnase1 | deoxyribonuclease I | NM_010061 | 1.54E-04 | -1.89 |
| 10347928 | Sp110 | Sp110 nuclear body protein | NM_175397 | 1.62E-04 | -1.66 |
| 10385526 | --- | --- | ENSMUST00000109210 | 1.62E-04 | -1.56 |
| 10582874 | Sp110 | Sp110 nuclear body protein | NM_175397 | 1.62E-04 | -1.96 |
| 10606016 | Il2rg | interleukin 2 receptor, gamma chain | NM_013563 | 1.62E-04 | -1.88 |
| 10531994 | Gbp6 | guanylate binding protein 6 | NM_194336 | 1.81E-04 | -1.88 |
| 10550749 | Nlrp9b | NLR family, pyrin domain containing 9B | NM_194058 | 2.00E-04 | -2.43 |
| 10545001 | Ppm1k | protein phosphatase 1K (PP2C domain containing) | NM_175523 | 2.05E-04 | -1.70 |
| 10605143 | Arhgap4 | Rho GTPase activating protein 4 | NM_138630 | 2.05E-04 | -1.54 |
| 10445803 | Unc5cl | unc-5 homolog C (C. elegans)-like | NM_152823 | 2.27E-04 | -1.74 |
| 10502791 | Ifi44 | interferon-induced protein 44 | NM_133871 | 3.04E-04 | -1.61 |
| 10581479 | Smpd3 | sphingomyelin phosphodiesterase 3, neutral | NM_021491 | 3.50E-04 | -1.72 |
| 10385513 | 9930111J21Rik2 | RIKEN cDNA 9930111J21 gene 2 | NM_173434 | 3.59E-04 | -2.00 |
| 10439268 | Dtx3l | deltex 3-like (Drosophila) | NM_001013371 | 3.61E-04 | -1.65 |
| 10425037 | Apol10a | apolipoprotein L 10a | NM_177744 | 3.87E-04 | -1.62 |
| 10369040 | Ros1 | Ros1 proto-oncogene | NM_011282 | 4.09E-04 | -2.01 |
| 10448350 | Paqr4 | progestin and adipoQ receptor family member IV | NM_023824 | 4.09E-04 | -1.51 |
| 10507784 | Ppt1 | palmitoyl-protein thioesterase 1 | NM_008917 | 4.09E-04 | -1.54 |
| 10347948 | Sp100 | nuclear antigen Sp100 | NM_013673 | 4.88E-04 | -1.63 |
| 10440918 | Tmem50b | transmembrane protein 50B | NM_030018 | 4.88E-04 | -1.59 |
| 10502042 | Alpk1 | alpha-kinase 1 | NM_027808 | 4.88E-04 | -1.55 |
| 10461487 | Cybasc3 | cytochrome b, ascorbate dependent 3 | NM_201351 | 5.64E-04 | -1.51 |
| 10545569 | Reg3g | regenerating islet-derived 3 gamma | NM_011260 | 5.67E-04 | -1.58 |
| 10422608 | Oxct1 | 3-oxoacid CoA transferase 1 | NM_024188 | 6.06E-04 | -1.74 |
| 10539179 | Reg3b | regenerating islet-derived 3 beta | NM_011036 | 6.79E-04 | -1.56 |
| 10344966 | Ly96 | lymphocyte antigen 96 | NM_016923 | 7.04E-04 | -2.99 |
| 10347933 | Sp140 | Sp140 nuclear body protein | NM_001013817 | 7.34E-04 | -2.25 |
| 10394060 | Sectm1b | secreted and transmembrane 1B | NM_026907 | 7.34E-04 | -1.74 |
| 10517165 | Cd52 | CD52 antigen | NM_013706 | 7.34E-04 | -1.66 |
| 10429588 | 9030619P08Rik | RIKEN cDNA 9030619P08 gene | NM_001039720 | 7.36E-04 | -1.54 |
| 10490150 | Zbp1 | Z-DNA binding protein 1 | NM_021394 | 7.74E-04 | -1.51 |
| 10368859 | Armc2 | armadillo repeat containing 2 | NM_001034858 | 8.52E-04 | -1.50 |
| 10444229 | H2-DMa | histocompatibility 2, class II, locus DMa | ENSMUST00000042121 | 8.87E-04 | -1.88 |
| 10478145 | Ppp1r16b | protein phosphatase 1, regulatory (inhibitor) subunit 16B | ENSMUST00000052927 | 8.87E-04 | -2.16 |
| 10534909 | Sp110 | Sp110 nuclear body protein | NM_175397 | 8.87E-04 | -1.81 |
| 10411459 | Tmem171 | transmembrane protein 171 | NM_001025606 | 9.74E-04 | -1.52 |
| 10582997 | Casp4 | caspase 4, apoptosis-related cysteine peptidase | ENSMUST00000027012 | 9.85E-04 | -1.76 |
| 10566132 | Rhog | ras homolog gene family, member G | NM_019566 | 1.00E-03 | -1.54 |
| 10480035 | Pfkfb3 | 6-phosphofructo-2-kinase/fructose-2,6-biphosphatase 3 | NM_001177753 | 1.01E-03 | -1.54 |
| 10538590 | Herc6 | hect domain and RLD 6 | NM_025992 | 1.01E-03 | -2.14 |
| 10379630 | Slfn2 | schlafen 2 | NM_011408 | 1.05E-03 | -1.54 |
| 10385504 | Gm5431 | predicted gene 5431 | ENSMUST00000109212 | 1.05E-03 | -1.71 |
| 10429564 | Ly6a | lymphocyte antigen 6 complex, locus A | NM_010738 | 1.05E-03 | -1.72 |
| 10559580 | Syt5 | synaptotagmin V | NM_016908 | 1.08E-03 | -1.54 |
| 10459905 | Setbp1 | SET binding protein 1 | NM_053099 | 1.10E-03 | -2.03 |
| 10460237 | Unc93b1 | unc-93 homolog B1 (C. elegans) | ENSMUST00000162708 | 1.14E-03 | -2.92 |
| 10566050 | Il18bp | interleukin 18 binding protein | NM_010531 | 1.14E-03 | -1.88 |
| 10505517 | Tlr4 | toll-like receptor 4 | ENSMUST00000048096 | 1.23E-03 | -1.55 |
| 10444554 | Slc44a4 | solute carrier family 44, member 4 | NM_023557 | 1.24E-03 | -1.52 |
| 10476276 | Mavs | mitochondrial antiviral signaling protein | NM_144888 | 1.25E-03 | -2.21 |
| 10358978 | Ier5 | immediate early response 5 | NM_010500 | 1.28E-03 | -1.92 |
| 10550994 | Ceacam10 | carcinoembryonic antigen-related cell adhesion molecule 10 | NM_007675 | 1.41E-03 | -1.97 |
| 10388914 | Ksr1 | kinase suppressor of ras 1 | NM_013571 | 1.48E-03 | -2.12 |
| 10414514 | Pnp | purine-nucleoside phosphorylase | NM_013632 | 1.48E-03 | -2.17 |
| 10491091 | Tnfsf10 | tumor necrosis factor (ligand) superfamily, member 10 | NM_009425 | 1.48E-03 | -2.17 |
| 10391207 | Dhx58 | DEXH (Asp-Glu-X-His) box polypeptide 58 | NM_030150 | 1.53E-03 | -1.86 |
| 10424213 | Zhx2 | zinc fingers and homeoboxes 2 | NM_199449 | 1.56E-03 | -1.96 |
| 10357660 | Mfsd4 | major facilitator superfamily domain containing 4 | NM_001114662 | 1.61E-03 | -2.27 |
| 10577792 | Plekha2 | pleckstrin homology domain-containing, family A (phosphoinositide binding specific) member 2 | ENSMUST00000064883 | 1.79E-03 | -2.19 |
| 10450145 | Psmb9 | proteasome (prosome, macropain) subunit, beta type 9 (large multifunctional peptidase 2) | NM_013585 | 1.80E-03 | -1.53 |
| 10525158 | Oas1b | 2'-5' oligoadenylate synthetase 1B | NR_003507 | 1.82E-03 | -6.76 |
| 10414527 | Pnp2 | purine-nucleoside phosphorylase 2 | NM_001123371 | 1.86E-03 | -2.45 |
| 10373367 | Coq10a | coenzyme Q10 homolog A (yeast) | NM_001081040 | 1.93E-03 | -1.59 |
| 10402347 | Ifi27l2a | interferon, alpha-inducible protein 27 like 2A | NM_029803 | 1.93E-03 | -2.13 |
| 10374236 | Upp1 | uridine phosphorylase 1 | NM_009477 | 1.97E-03 | -1.62 |
| 10409278 | Nfil3 | nuclear factor, interleukin 3, regulated | NM_017373 | 1.98E-03 | -1.50 |
| 10415293 | Rnf31 | ring finger protein 31 | NM_194346 | 2.01E-03 | -1.57 |
| 10519268 | Mir200b | microRNA 200b | NR_029587 | 2.07E-03 | -1.54 |
| 10514912 | Dio1 | deiodinase, iodothyronine, type I | NM_007860 | 2.08E-03 | -2.34 |
| 10502050 | Alpk1 | alpha-kinase 1 | NM_027808 | 2.12E-03 | -1.70 |
| 10535524 | Ocm | oncomodulin | NM_033039 | 2.14E-03 | -1.65 |
| 10509596 | Rnf186 | ring finger protein 186 | NM_025786 | 2.16E-03 | -2.21 |
| 10585186 | 1600029D21Rik | RIKEN cDNA 1600029D21 gene | NM_029639 | 2.16E-03 | -2.02 |
| 10552406 | Nkg7 | natural killer cell group 7 sequence | ENSMUST00000070518 | 2.21E-03 | -1.54 |
| 10598083 | --- | --- | NC_005089 | 2.23E-03 | -2.04 |
| 10356278 | Sp110 | Sp110 nuclear body protein | NM_175397 | 2.24E-03 | -1.60 |
| 10502774 | Lphn2 | latrophilin 2 | NM_001081298 | 2.29E-03 | -1.54 |
| 10566358 | Trim30a | tripartite motif-containing 30A | NM_009099 | 2.29E-03 | -1.91 |
| 10356262 | --- | --- | ENSMUST00000073378 | 2.35E-03 | -1.69 |
| 10578071 | Wrn | Werner syndrome homolog (human) | NM_011721 | 2.36E-03 | -2.00 |
| 10346191 | Stat1 | signal transducer and activator of transcription 1 | NM_001205313 | 2.37E-03 | -1.98 |
| 10350742 | Rnasel | ribonuclease L (2', 5'-oligoisoadenylate synthetase-dependent) | NM_011882 | 2.37E-03 | -1.86 |
| 10376060 | Irf1 | interferon regulatory factor 1 | NM_008390 | 2.43E-03 | -1.51 |
| 10405619 | Idnk | idnK gluconokinase homolog (E. coli) | NM_198004 | 2.47E-03 | -1.81 |
| 10440593 | Rwdd2b | RWD domain containing 2B | NM_016924 | 2.57E-03 | -1.66 |
| 10462623 | Ifit1 | interferon-induced protein with tetratricopeptide repeats 1 | NM_008331 | 2.57E-03 | -1.63 |
| 10566326 | Trim12a | tripartite motif-containing 12A | NM_023835 | 2.58E-03 | -2.33 |
| 10593646 | Tnfaip8l3 | tumor necrosis factor, alpha-induced protein 8-like 3 | NM_001033535 | 2.74E-03 | -1.51 |
| 10502052 | Alpk1 | alpha-kinase 1 | NM_027808 | 2.84E-03 | -1.56 |
| 10521391 | Acox3 | acyl-Coenzyme A oxidase 3, pristanoyl | NM_030721 | 2.84E-03 | -1.59 |
| 10556302 | Ampd3 | adenosine monophosphate deaminase 3 | NM_009667 | 2.92E-03 | -2.18 |
| 10470027 | Npdc1 | neural proliferation, differentiation and control gene 1 | NM_008721 | 2.98E-03 | -4.01 |
| 10475517 | AA467197 | expressed sequence AA467197 | ENSMUST00000047498 | 2.99E-03 | -1.85 |
| 10426606 | 4930415O20Rik | RIKEN cDNA 4930415O20 gene | NM_001201322 | 3.02E-03 | -1.67 |
| 10490611 | Ptk6 | PTK6 protein tyrosine kinase 6 | NM_009184 | 3.02E-03 | -2.67 |
| 10420198 | Ripk3 | receptor-interacting serine-threonine kinase 3 | NM_019955 | 3.04E-03 | -1.63 |
| 10371846 | Apaf1 | apoptotic peptidase activating factor 1 | NM_001042558 | 3.05E-03 | -2.28 |
| 10587854 | Slc9a9 | solute carrier family 9 (sodium/hydrogen exchanger), member 9 | NM_177909 | 3.12E-03 | -1.66 |
| 10487748 | 4930402H24Rik | RIKEN cDNA 4930402H24 gene | BC052447 | 3.16E-03 | -1.92 |
| 10356299 | Gpr55 | G protein-coupled receptor 55 | NM_001033290 | 3.23E-03 | -3.77 |
| 10502780 | Lphn2 | latrophilin 2 | NM_001081298 | 3.23E-03 | -1.55 |
| 10466165 | --- | --- | ENSMUST00000167437 | 3.28E-03 | -1.72 |
| 10346607 | Fzd7 | frizzled homolog 7 (Drosophila) | NM_008057 | 3.36E-03 | -2.25 |
| 10367224 | Stat2 | signal transducer and activator of transcription 2 | NM_019963 | 3.36E-03 | -1.61 |
| 10435457 | Parp9 | poly (ADP-ribose) polymerase family, member 9 | NM_030253 | 3.36E-03 | -1.71 |
| 10499777 | Ints3 | integrator complex subunit 3 | NM_145540 | 3.36E-03 | -1.62 |
| 10569707 | Myadm | myeloid-associated differentiation marker | ENSMUST00000096744 | 3.36E-03 | -1.57 |
| 10502766 | Lphn2 | latrophilin 2 | NM_001081298 | 3.41E-03 | -1.84 |
| 10424676 | Ly6e | lymphocyte antigen 6 complex, locus E | NM_001164036 | 3.46E-03 | -2.06 |
| 10566767 | St5 | suppression of tumorigenicity 5 | NM_001001326 | 3.58E-03 | -1.50 |
| 10492689 | Pdgfc | platelet-derived growth factor, C polypeptide | NM_019971 | 3.73E-03 | -1.61 |
| 10531415 | Cxcl10 | chemokine (C-X-C motif) ligand 10 | NM_021274 | 3.80E-03 | -1.68 |
| 10383233 | Rnf213 | ring finger protein 213 | AK173199 | 3.88E-03 | -1.55 |
| 10439249 | Parp14 | poly (ADP-ribose) polymerase family, member 14 | NM_001039530 | 3.93E-03 | -1.62 |
| 10381049 | Rapgefl1 | Rap guanine nucleotide exchange factor (GEF)-like 1 | NM_001080925 | 4.15E-03 | -1.51 |
| 10383214 | Rnf213 | ring finger protein 213 | AK173199 | 4.26E-03 | -1.79 |
| 10478169 | Dhx35 | DEAH (Asp-Glu-Ala-His) box polypeptide 35 | NM_145742 | 4.32E-03 | -1.56 |
| 10489204 | Tgm2 | transglutaminase 2, C polypeptide | NM_009373 | 4.48E-03 | -2.36 |
| 10530145 | Tlr1 | toll-like receptor 1 | NM_030682 | 4.93E-03 | -1.80 |
| 10563362 | Sphk2 | sphingosine kinase 2 | NM_020011 | 4.93E-03 | -1.76 |
| 10508392 | Rnf19b | ring finger protein 19B | NM_029219 | 4.96E-03 | -2.30 |
| 10531407 | Cxcl9 | chemokine (C-X-C motif) ligand 9 | NM_008599 | 4.96E-03 | -1.57 |
| 10582303 | Cyba | cytochrome b-245, alpha polypeptide | NM_007806 | 5.04E-03 | -2.08 |
| 10420488 | D14Ertd668e | DNA segment, Chr 14, ERATO Doi 668, expressed | NM_199015 | 5.27E-03 | -1.59 |
| 10363231 | Smpdl3a | sphingomyelin phosphodiesterase, acid-like 3A | NM_020561 | 5.40E-03 | -1.54 |
| 10394068 | Sectm1a | secreted and transmembrane 1A | NM_145373 | 5.41E-03 | -1.60 |
| 10469984 | Lrrc26 | leucine rich repeat containing 26 | NM_146117 | 5.50E-03 | -1.61 |
| 10379953 | 4632419I22Rik | RIKEN cDNA 4632419I22 gene | BC067002 | 5.59E-03 | -2.14 |
| 10512470 | Cd72 | CD72 antigen | NM_001110320 | 5.71E-03 | -1.77 |
| 10360070 | Fcer1g | Fc receptor, IgE, high affinity I, gamma polypeptide | NM_010185 | 5.74E-03 | -1.52 |
| 10404606 | Ly86 | lymphocyte antigen 86 | NM_010745 | 5.78E-03 | -1.62 |
| 10383196 | Rnf213 | ring finger protein 213 | ENSMUST00000131035 | 5.80E-03 | -1.53 |
| 10582862 | Arhgef12 | Rho guanine nucleotide exchange factor (GEF) 12 | NM_027144 | 5.81E-03 | -2.27 |
| 10530627 | Lrrc66 | leucine rich repeat containing 66 | NM_153568 | 5.90E-03 | -2.30 |
| 10367045 | Rdh16 | retinol dehydrogenase 16 | NM_009040 | 6.18E-03 | -2.22 |
| 10450819 | Znrd1 | zinc ribbon domain containing, 1 | ENSMUST00000113669 | 6.18E-03 | -3.36 |
| 10563350 | Fut2 | fucosyltransferase 2 | ENSMUST00000069800 | 6.22E-03 | -1.52 |
| 10541307 | Usp18 | ubiquitin specific peptidase 18 | NM_011909 | 6.24E-03 | -1.54 |
| 10368806 | Smpd2 | sphingomyelin phosphodiesterase 2, neutral | NM_009213 | 6.25E-03 | -1.58 |
| 10362811 | Sesn1 | sestrin 1 | NM_001162908 | 6.33E-03 | -1.60 |
| 10383202 | Rnf213 | ring finger protein 213 | ENSMUST00000131035 | 6.33E-03 | -1.53 |
| 10430174 | Apol9a | apolipoprotein L 9a | NM_173786 | 6.33E-03 | -1.55 |
| 10383194 | Rnf213 | ring finger protein 213 | ENSMUST00000131035 | 6.46E-03 | -1.53 |
| 10406176 | Slc9a3 | solute carrier family 9 (sodium/hydrogen exchanger), member 3 | NM_001081060 | 6.49E-03 | -1.55 |
| 10389581 | Ypel2 | yippee-like 2 (Drosophila) | NM_001005341 | 6.53E-03 | -1.61 |
| 10533246 | Oas1g | 2'-5' oligoadenylate synthetase 1G | NM_011852 | 6.53E-03 | -3.57 |
| 10530612 | Fryl | furry homolog-like (Drosophila) | NM_028194 | 6.67E-03 | -2.62 |
| 10349782 | Nuak2 | NUAK family, SNF1-like kinase, 2 | NM_001195025 | 6.78E-03 | -1.54 |
| 10524621 | Oasl2 | 2'-5' oligoadenylate synthetase-like 2 | NM_011854 | 6.78E-03 | -1.72 |
| 10460767 | Batf2 | basic leucine zipper transcription factor, ATF-like 2 | ENSMUST00000045042 | 6.80E-03 | -1.63 |
| 10462613 | Ifit2 | interferon-induced protein with tetratricopeptide repeats 2 | NM_008332 | 6.83E-03 | -2.08 |
| 10561927 | Aplp1 | amyloid beta (A4) precursor-like protein 1 | NM_007467 | 7.10E-03 | -1.51 |
| 10603208 | Mid1 | midline 1 | NM_010797 | 7.10E-03 | -1.59 |
| 10383204 | Rnf213 | ring finger protein 213 | ENSMUST00000131035 | 7.16E-03 | -2.19 |
| 10365640 | Slc5a8 | solute carrier family 5 (iodide transporter), member 8 | NM_145423 | 7.16E-03 | -2.92 |
| 10576757 | Fcer2a | Fc receptor, IgE, low affinity II, alpha polypeptide | ENSMUST00000005678 | 7.30E-03 | -1.73 |
| 10417526 | Dnase1l3 | deoxyribonuclease 1-like 3 | NM_007870 | 7.39E-03 | -3.10 |
| 10569203 | Chid1 | chitinase domain containing 1 | NM_001142681 | 7.92E-03 | -1.51 |
| 10366052 | Kitl | kit ligand | NM_013598 | 8.00E-03 | -2.72 |
| 10360391 | Ifi203 | interferon activated gene 203 | NM_001045481 | 8.20E-03 | -2.25 |
| 10390075 | Gm11545 | predicted gene 11545 | NM_001105561 | 8.32E-03 | -2.06 |
| 10492997 | Etv3 | ets variant gene 3 | NM_001083318 | 8.55E-03 | -1.58 |
| 10546685 | Eif4e3 | eukaryotic translation initiation factor 4E member 3 | NM_025829 | 8.57E-03 | -1.96 |
| 10598203 | Ccl28 | chemokine (C-C motif) ligand 28 | NM_020279 | 9.07E-03 | -1.52 |
| 10502748 | Lphn2 | latrophilin 2 | NM_001081298 | 9.09E-03 | -1.78 |
| 10566333 | Trim12c | tripartite motif-containing 12C | NM_001146007 | 9.09E-03 | -1.63 |
| 10569168 | Slc25a22 | solute carrier family 25 (mitochondrial carrier, glutamate), member 22 | NM_026646 | 9.09E-03 | -1.83 |
| 10516266 | Zc3h12a | zinc finger CCCH type containing 12A | NM_153159 | 9.27E-03 | -1.80 |
| 10358027 | Elf3 | E74-like factor 3 | NM_001163131 | 9.32E-03 | -1.80 |
| 10378833 | Ssh2 | slingshot homolog 2 (Drosophila) | NM_177710 | 9.45E-03 | -1.53 |
| 10501063 | Cd53 | CD53 antigen | NM_007651 | 9.65E-03 | -1.55 |
| 10477644 | Trp53inp2 | transformation related protein 53 inducible nuclear protein 2 | NM_178111 | 9.70E-03 | -1.75 |
| 10606792 | Nxf7 | nuclear RNA export factor 7 | ENSMUST00000113163 | 9.70E-03 | -1.50 |
| 10435112 | Muc4 | mucin 4 | NM_080457 | 9.70E-03 | -2.64 |
| 10565994 | Art2b | ADP-ribosyltransferase 2b | NM_019915 | 9.70E-03 | -2.17 |
| 10462473 | Mbl2 | mannose-binding lectin (protein C) 2 | NM_010776 | 9.93E-03 | -2.09 |
| 10496555 | Gbp1 | guanylate binding protein 1 | NM_010259 | 1.02E-02 | -1.89 |
| 10383556 | Fn3krp | fructosamine 3 kinase related protein | NM_181420 | 1.03E-02 | -1.63 |
| 10425049 | Apol9b | apolipoprotein L 9b | NM_173743 | 1.05E-02 | -2.02 |
| 10496569 | Gbp7 | guanylate binding protein 7 | NM_145545 | 1.08E-02 | -1.64 |
| 10423556 | Pgcp | plasma glutamate carboxypeptidase | NM_018755 | 1.12E-02 | -1.95 |
| 10346348 | Spats2l | spermatogenesis associated, serine-rich 2-like | NM_144882 | 1.17E-02 | -1.78 |
| 10383212 | Rnf213 | ring finger protein 213 | ENSMUST00000131035 | 1.17E-02 | -4.94 |
| 10529977 | Ppargc1a | peroxisome proliferative activated receptor, gamma, coactivator 1 alpha | NM_008904 | 1.22E-02 | -1.77 |
| 10378068 | Xaf1 | XIAP associated factor 1 | NM_001037713 | 1.22E-02 | -1.70 |
| 10467493 | Tctn3 | tectonic family member 3 | NM_026260 | 1.23E-02 | -1.59 |
| 10521892 | Slc34a2 | solute carrier family 34 (sodium phosphate), member 2 | NM_011402 | 1.23E-02 | -1.60 |
| 10475448 | Duoxa2 | dual oxidase maturation factor 2 | NM_025777 | 1.27E-02 | -1.56 |
| 10486956 | Duox2 | dual oxidase 2 | NM_177610 | 1.27E-02 | -1.80 |
| 10500434 | Bcl9 | B cell CLL/lymphoma 9 | ENSMUST00000046521 | 1.30E-02 | -4.96 |
| 10474958 | Dll4 | delta-like 4 (Drosophila) | NM_019454 | 1.31E-02 | -1.58 |
| 10479165 | Edn3 | endothelin 3 | NM_007903 | 1.32E-02 | -1.93 |
| 10378549 | Rtn4rl1 | reticulon 4 receptor-like 1 | ENSMUST00000102514 | 1.32E-02 | -2.00 |
| 10444824 | H2-Q6 | histocompatibility 2, Q region locus 6 | NM_207648 | 1.33E-02 | -1.65 |
| 10451665 | Apobec2 | apolipoprotein B mRNA editing enzyme, catalytic polypeptide 2 | NM_009694 | 1.34E-02 | -6.48 |
| 10361282 | Cr1l | complement component (3b/4b) receptor 1-like | NM_013499 | 1.35E-02 | -2.37 |
| 10379228 | Nos2 | nitric oxide synthase 2, inducible | ENSMUST00000018610 | 1.39E-02 | -1.72 |
| 10429568 | Ly6c1 | lymphocyte antigen 6 complex, locus C1 | NM_010741 | 1.41E-02 | -1.54 |
| 10430166 | Apol7a | apolipoprotein L 7a | NM_029419 | 1.47E-02 | -1.65 |
| 10576784 | Cd209a | CD209a antigen | AF373408 | 1.48E-02 | -1.58 |
| 10448402 | Prss30 | protease, serine, 30 | NM_013921 | 1.48E-02 | -1.82 |
| 10366983 | Tmem194 | transmembrane protein 194 | NM_001113211 | 1.48E-02 | -1.56 |
| 10571984 | Ddx60 | DEAD (Asp-Glu-Ala-Asp) box polypeptide 60 | NM_001081215 | 1.49E-02 | -1.57 |
| 10377372 | Slc25a35 | solute carrier family 25, member 35 | NM_028048 | 1.51E-02 | -1.79 |
| 10385518 | Tgtp1 | T cell specific GTPase 1 | NM_011579 | 1.51E-02 | -2.03 |
| 10560685 | Bcl3 | B cell leukemia/lymphoma 3 | NM_033601 | 1.51E-02 | -2.15 |
| 10489850 | Znfx1 | zinc finger, NFX1-type containing 1 | NM_001033196 | 1.56E-02 | -1.67 |
| 10556463 | Arntl | aryl hydrocarbon receptor nuclear translocator-like | NM_007489 | 1.56E-02 | -1.69 |
| 10607619 | Cdkl5 | cyclin-dependent kinase-like 5 | NM_001024624 | 1.57E-02 | -1.74 |
| 10601312 | Chic1 | cysteine-rich hydrophobic domain 1 | NM_009767 | 1.58E-02 | -1.90 |
| 10475544 | Sema6d | sema domain, transmembrane domain (TM), and cytoplasmic domain, (semaphorin) 6D | NM_199241 | 1.60E-02 | -2.82 |
| 10579799 | Tmem184c | transmembrane protein 184C | NM_145599 | 1.62E-02 | -3.23 |
| 10508663 | Laptm5 | lysosomal-associated protein transmembrane 5 | NM_010686 | 1.62E-02 | -1.60 |
| 10437224 | Mx2 | myxovirus (influenza virus) resistance 2 | NR_003508 | 1.64E-02 | -2.55 |
| 10361091 | Atf3 | activating transcription factor 3 | NM_007498 | 1.68E-02 | -1.85 |
| 10395039 | Cmpk2 | cytidine monophosphate (UMP-CMP) kinase 2, mitochondrial | NM_020557 | 1.74E-02 | -3.68 |
| 10502778 | Lphn2 | latrophilin 2 | NM_001081298 | 1.75E-02 | -1.70 |
| 10466528 | Rfk | riboflavin kinase | NM_019437 | 1.78E-02 | -1.66 |
| 10441361 | Tiam2 | T cell lymphoma invasion and metastasis 2 | NM_001122998 | 1.84E-02 | -1.90 |
| 10491300 | Skil | SKI-like | NM_011386 | 1.86E-02 | -1.62 |
| 10444244 | Tap1 | transporter 1, ATP-binding cassette, sub-family B (MDR/TAP) | NM_013683 | 1.86E-02 | -1.72 |
| 10533198 | Oas2 | 2'-5' oligoadenylate synthetase 2 | NM_145227 | 1.98E-02 | -3.20 |
| 10446763 | Lbh | limb-bud and heart | NM_029999 | 1.99E-02 | -1.77 |
| 10450344 | C2 | complement component 2 (within H-2S) | NM_013484 | 1.99E-02 | -1.63 |
| 10529979 | Ppargc1a | peroxisome proliferative activated receptor, gamma, coactivator 1 alpha | NR_027710 | 2.00E-02 | -1.65 |
| 10453544 | Mettl4 | methyltransferase like 4 | NM_176917 | 2.04E-02 | -1.87 |
| 10351801 | Igsf9 | immunoglobulin superfamily, member 9 | NM_033608 | 2.05E-02 | -1.59 |
| 10376324 | Gm12250 | predicted gene 12250 | NM_001135115 | 2.12E-02 | -1.53 |
| 10435508 | 2010005H15Rik | RIKEN cDNA 2010005H15 gene | NM_029733 | 2.16E-02 | -2.21 |
| 10473384 | Slc43a3 | solute carrier family 43, member 3 | NM_021398 | 2.19E-02 | -2.62 |
| 10462618 | Ifit3 | interferon-induced protein with tetratricopeptide repeats 3 | NM_010501 | 2.26E-02 | -1.62 |
| 10443007 | Neurl1b | neuralized homolog 1b (Drosophila) | NM_001081656 | 2.26E-02 | -1.51 |
| 10378855 | Ssh2 | slingshot homolog 2 (Drosophila) | NM_177710 | 2.31E-02 | -2.73 |
| 10383210 | Rnf213 | ring finger protein 213 | ENSMUST00000131035 | 2.32E-02 | -1.56 |
| 10450161 | H2-Ea-ps | histocompatibility 2, class II antigen E alpha, pseudogene | NM_010381 | 2.32E-02 | -1.90 |
| 10495574 | Sass6 | spindle assembly 6 homolog (C. elegans) | NM_028349 | 2.32E-02 | -1.81 |
| 10383152 | Rnf213 | ring finger protein 213 | ENSMUST00000131035 | 2.35E-02 | -1.54 |
| 10470562 | --- | --- | ENSMUST00000074761 | 2.37E-02 | -1.65 |
| 10548450 | Klra5 | killer cell lectin-like receptor, subfamily A, member 5 | ENSMUST00000118060 | 2.40E-02 | -1.53 |
| 10501629 | Cdc14a | CDC14 cell division cycle 14A | NM_001080818 | 2.49E-02 | -2.83 |
| 10383047 | Enpp7 | ectonucleotide pyrophosphatase/phosphodiesterase 7 | NM_001030291 | 2.50E-02 | -1.76 |
| 10549473 | Caprin2 | caprin family member 2 | NM_181541 | 2.52E-02 | -1.61 |
| 10366144 | Mgat4c | mannosyl (alpha-1,3-)-glycoprotein beta-1,4-N-acetylglucosaminyltransferase, isozyme C (putative) | NM_001162369 | 2.55E-02 | -1.66 |
| 10433507 | Ciita | class II transactivator | NM_001243760 | 2.59E-02 | -1.58 |
| 10405587 | Tgfbi | transforming growth factor, beta induced | ENSMUST00000045173 | 2.84E-02 | -1.84 |
| 10562812 | Spib | Spi-B transcription factor (Spi-1/PU.1 related) | NM_019866 | 2.90E-02 | -1.67 |
| 10466735 | Fam189a2 | family with sequence similarity 189, member A2 | NM_001114174 | 2.94E-02 | -2.13 |
| 10569278 | Dusp8 | dual specificity phosphatase 8 | NM_008748 | 2.99E-02 | -1.65 |
| 10574166 | Cpne2 | copine II | NM_153507 | 3.04E-02 | -2.07 |
| 10385533 | Gm12185 | predicted gene 12185 | ENSMUST00000094476 | 3.13E-02 | -1.90 |
| 10526502 | Rabl5 | RAB, member of RAS oncogene family-like 5 | NM_026073 | 3.21E-02 | -1.56 |
| 10561055 | Ceacam2 | carcinoembryonic antigen-related cell adhesion molecule 2 | NM_001113368 | 3.22E-02 | -1.69 |
| 10363161 | Tmem229b | transmembrane protein 229B | NM_178745 | 3.32E-02 | -1.59 |
| 10401109 | Gpx2 | glutathione peroxidase 2 | NM_030677 | 3.35E-02 | -1.75 |
| 10601612 | Atrn | attractin | NM_009730 | 3.38E-02 | -1.74 |
| 10423049 | Prlr | prolactin receptor | NM_011169 | 3.58E-02 | -1.72 |
| 10545237 | --- | --- | ENSMUST00000103384 | 3.66E-02 | -1.88 |
| 10550740 | Ceacam20 | carcinoembryonic antigen-related cell adhesion molecule 20 | NM_027839 | 3.73E-02 | -1.88 |
| 10374727 | Bcl11a | B cell CLL/lymphoma 11A (zinc finger protein) | NM_016707 | 3.74E-02 | -1.87 |
| 10349051 | Tnfrsf11a | tumor necrosis factor receptor superfamily, member 11a | NM_009399 | 3.76E-02 | -4.39 |
| 10570018 | Tnfsf13b | tumor necrosis factor (ligand) superfamily, member 13b | ENSMUST00000164629 | 3.76E-02 | -1.54 |
| 10497817 | Anxa5 | annexin A5 | NM_009673 | 3.84E-02 | -1.55 |
| 10353716 | Gm6489 | Aly/REF export factor pseudogene | XM_003086145 | 3.94E-02 | -1.99 |
| 10461594 | Ms4a4c | membrane-spanning 4-domains, subfamily A, member 4C | NM_029499 | 4.01E-02 | -1.69 |
| 10533256 | Oas1a | 2'-5' oligoadenylate synthetase 1A | NM_145211 | 4.23E-02 | -1.66 |
| 10589884 | Bcl2a1c | B cell leukemia/lymphoma 2 related protein A1c | NM_007535 | 4.42E-02 | -1.69 |
| 10438415 | --- | --- | M34598 | 4.42E-02 | -1.63 |
| 10496001 | Cfi | complement component factor i | NM_007686 | 4.52E-02 | -1.65 |
| 10572733 | Zfp961 | zinc finger protein 961 | NM_001164581 | 4.63E-02 | -1.96 |
| 10563178 | Cd37 | CD37 antigen | NM_007645 | 4.76E-02 | -2.18 |
| 10434778 | Rtp4 | receptor transporter protein 4 | NM_023386 | 4.82E-02 | -1.68 |
| 10502335 | Bank1 | B cell scaffold protein with ankyrin repeats 1 | NM_001033350 | 4.98E-02 | -1.66 |

**(B)** Positive-regulated DEGs

| **Probsets ID** | **Gene Symbol** | **Gene Description** | **mRNA ID** | **adj. *P*-value** | **Fold Change** |
| --- | --- | --- | --- | --- | --- |
| 10356082 | Plscr1 | phospholipid scramblase 1 | NM_011636 | 1.54E-04 | 1.76 |
| 10406050 | Slc6a3 | solute carrier family 6 (neurotransmitter transporter, dopamine), member 3 | NM_010020 | 1.62E-04 | 1.79 |
| 10417544 | Acox2 | acyl-Coenzyme A oxidase 2, branched chain | NM_053115 | 1.62E-04 | 3.00 |
| 10430794 | Pmm1 | phosphomannomutase 1 | NM_013872 | 1.62E-04 | 1.58 |
| 10479047 | Pck1 | phosphoenolpyruvate carboxykinase 1, cytosolic | NM_011044 | 1.62E-04 | 2.11 |
| 10501235 | Gstm4 | glutathione S-transferase, mu 4 | NM_026764 | 1.62E-04 | 1.51 |
| 10589654 | Als2cl | ALS2 C-terminal like | NM_001146059 | 1.62E-04 | 1.74 |
| 10461979 | Aldh1a1 | aldehyde dehydrogenase family 1, subfamily A1 | NM_013467 | 1.76E-04 | 1.57 |
| 10352838 | Lamb3 | laminin, beta 3 | NM_008484 | 1.81E-04 | 2.98 |
| 10397364 | Mfsd7c | major facilitator superfamily domain containing 7C | NM_145447 | 2.00E-04 | 1.91 |
| 10587792 | Plscr1 | phospholipid scramblase 1 | NM_011636 | 2.00E-04 | 2.32 |
| 10386683 | Slc47a1 | solute carrier family 47, member 1 | NM_026183 | 2.05E-04 | 1.97 |
| 10394978 | Rrm2 | ribonucleotide reductase M2 | NM_009104 | 2.05E-04 | 2.81 |
| 10519527 | Abcb1a | ATP-binding cassette, sub-family B (MDR/TAP), member 1A | NM_011076 | 2.27E-04 | 1.75 |
| 10565255 | 9930013L23Rik | RIKEN cDNA 9930013L23 gene | NM_030728 | 2.27E-04 | 1.98 |
| 10355205 | D630023F18Rik | RIKEN cDNA D630023F18 gene | BC137870 | 2.56E-04 | 1.56 |
| 10424667 | 4930572J05Rik | RIKEN cDNA 4930572J05 gene | NM_198607 | 2.56E-04 | 1.80 |
| 10515201 | Cyp4b1 | cytochrome P450, family 4, subfamily b, polypeptide 1 | NM_007823 | 2.73E-04 | 1.74 |
| 10574498 | Ces2a | carboxylesterase 2A | NM_133960 | 3.23E-04 | 2.24 |
| 10542470 | Mgst1 | microsomal glutathione S-transferase 1 | NM_019946 | 3.45E-04 | 3.07 |
| 10528090 | Rundc3b | RUN domain containing 3B | NM_198620 | 3.59E-04 | 1.78 |
| 10370054 | Slc5a4b | solute carrier family 5 (neutral amino acid transporters, system A), member 4b | NM_023219 | 3.59E-04 | 1.84 |
| 10554693 | Stard5 | StAR-related lipid transfer (START) domain containing 5 | NM_023377 | 3.59E-04 | 1.86 |
| 10360684 | Ephx1 | epoxide hydrolase 1, microsomal | NM_010145 | 3.59E-04 | 1.62 |
| 10464328 | Pnliprp2 | pancreatic lipase-related protein 2 | NM_011128 | 4.09E-04 | 1.82 |
| 10540028 | Klf15 | Kruppel-like factor 15 | NM_023184 | 4.22E-04 | 1.76 |
| 10466624 | Aldh1a7 | aldehyde dehydrogenase family 1, subfamily A7 | NM_011921 | 4.24E-04 | 1.86 |
| 10580678 | Ces1g | carboxylesterase 1G | NM_021456 | 6.06E-04 | 2.83 |
| 10501229 | Gstm1 | glutathione S-transferase, mu 1 | NM_010358 | 6.58E-04 | 1.60 |
| 10595148 | Gsta2 | glutathione S-transferase, alpha 2 (Yc2) | NM_008182 | 6.58E-04 | 1.50 |
| 10366275 | --- | --- | ENSMUST00000083719 | 7.02E-04 | 1.59 |
| 10595480 | Me1 | malic enzyme 1, NADP(+)-dependent, cytosolic | NM_008615 | 7.02E-04 | 1.65 |
| 10597619 | Slc22a13b | solute carrier family 22 (organic cation transporter), member 13b | NR_033303 | 7.05E-04 | 3.06 |
| 10394990 | Mboat2 | membrane bound O-acyltransferase domain containing 2 | NM_026037 | 7.34E-04 | 4.82 |
| 10405753 | Me1 | malic enzyme 1, NADP(+)-dependent, cytosolic | NM_008615 | 7.34E-04 | 1.81 |
| 10508721 | Snora44 | small nucleolar RNA, H/ACA box 44 | NR_034050 | 7.34E-04 | 1.84 |
| 10580635 | Ces1d | carboxylesterase 1D | NM_053200 | 7.34E-04 | 5.25 |
| 10581538 | Nqo1 | NAD(P)H dehydrogenase, quinone 1 | NM_008706 | 7.34E-04 | 2.10 |
| 10587339 | Gm10639 | predicted gene 10639 | NM_001122660 | 7.34E-04 | 1.51 |
| 10485700 | Bbox1 | butyrobetaine (gamma), 2-oxoglutarate dioxygenase 1 (gamma-butyrobetaine hydroxylase) | ENSMUST00000046233 | 8.15E-04 | 1.55 |
| 10449452 | Fkbp5 | FK506 binding protein 5 | NM_010220 | 9.35E-04 | 1.64 |
| 10491952 | Mgst2 | microsomal glutathione S-transferase 2 | NM_174995 | 9.72E-04 | 1.78 |
| 10428814 | Anxa13 | annexin A13 | NM_027211 | 9.74E-04 | 1.57 |
| 10590957 | Fut4 | fucosyltransferase 4 | NM_010242 | 1.01E-03 | 2.24 |
| 10492300 | Aadac | arylacetamide deacetylase (esterase) | NM_023383 | 1.01E-03 | 1.53 |
| 10531910 | Hsd17b13 | hydroxysteroid (17-beta) dehydrogenase 13 | NM_001163486 | 1.01E-03 | 1.94 |
| 10358879 | Npl | N-acetylneuraminate pyruvate lyase | NM_028749 | 1.03E-03 | 2.27 |
| 10397148 | Acot1 | acyl-CoA thioesterase 1 | NM_012006 | 1.03E-03 | 1.65 |
| 10360840 | Mosc1 | MOCO sulphurase C-terminal domain containing 1 | NM_001081361 | 1.06E-03 | 1.51 |
| 10364712 | Cirbp | cold inducible RNA binding protein | NM_007705 | 1.11E-03 | 4.02 |
| 10576581 | Kcnk1 | potassium channel, subfamily K, member 1 | NM_008430 | 1.11E-03 | 2.13 |
| 10500272 | Gm129 | predicted gene 129 | BC132471 | 1.14E-03 | 1.67 |
| 10390748 | Tns4 | tensin 4 | NM_172564 | 1.28E-03 | 2.79 |
| 10425987 | Ppara | peroxisome proliferator activated receptor alpha | NM_011144 | 1.34E-03 | 2.34 |
| 10462281 | Vldlr | very low density lipoprotein receptor | NM_013703 | 1.34E-03 | 5.57 |
| 10475990 | Slc20a1 | solute carrier family 20, member 1 | NM_015747 | 1.35E-03 | 1.87 |
| 10574572 | Ces2g | carboxylesterase 2G | NM_197999 | 1.41E-03 | 1.53 |
| 10603087 | Pir | pirin | NM_027153 | 1.41E-03 | 1.74 |
| 10413047 | Plau | plasminogen activator, urokinase | NM_008873 | 1.48E-03 | 1.65 |
| 10429140 | Ndrg1 | N-myc downstream regulated gene 1 | NM_008681 | 1.48E-03 | 2.99 |
| 10403312 | Akr1c19 | aldo-keto reductase family 1, member C19 | NM_001013785 | 1.57E-03 | 1.92 |
| 10436978 | Cbr3 | carbonyl reductase 3 | NM_173047 | 1.62E-03 | 1.96 |
| 10391454 | Vat1 | vesicle amine transport protein 1 homolog (T californica) | NM_012037 | 1.66E-03 | 1.57 |
| 10512949 | Abca1 | ATP-binding cassette, sub-family A (ABC1), member 1 | NM_013454 | 1.66E-03 | 1.85 |
| 10501222 | Gstm2 | glutathione S-transferase, mu 2 | ENSMUST00000012348 | 1.86E-03 | 1.96 |
| 10594988 | Mapk6 | mitogen-activated protein kinase 6 | NM_015806 | 1.98E-03 | 2.70 |
| 10587331 | Gm3776 | predicted gene 3776 | NM_001243092 | 2.00E-03 | 1.51 |
| 10454580 | Bin1 | bridging integrator 1 | NM_009668 | 2.01E-03 | 1.65 |
| 10345065 | Gsta3 | glutathione S-transferase, alpha 3 | NM_001077353 | 2.04E-03 | 1.67 |
| 10506470 | 1700024P16Rik | RIKEN cDNA 1700024P16 gene | NM_001162980 | 2.04E-03 | 1.78 |
| 10587323 | Gsta1 | glutathione S-transferase, alpha 1 (Ya) | NM_008181 | 2.05E-03 | 2.20 |
| 10352097 | 1700016C15Rik | RIKEN cDNA 1700016C15 gene | NM_027077 | 2.11E-03 | 1.88 |
| 10514510 | Cyp2j6 | cytochrome P450, family 2, subfamily j, polypeptide 6 | NM_010008 | 2.16E-03 | 3.37 |
| 10580649 | Ces1e | carboxylesterase 1E | NM_133660 | 2.18E-03 | 1.81 |
| 10528200 | Hnrnpa3 | heterogeneous nuclear ribonucleoprotein A3 | NM_053263 | 2.21E-03 | 1.85 |
| 10528207 | Cd36 | CD36 antigen | NM_001159557 | 2.21E-03 | 1.76 |
| 10344741 | Hnrnpa3 | heterogeneous nuclear ribonucleoprotein A3 | NM_053263 | 2.22E-03 | 1.60 |
| 10515113 | Hnrnpa3 | heterogeneous nuclear ribonucleoprotein A3 | NM_053263 | 2.22E-03 | 1.54 |
| 10491083 | Nceh1 | arylacetamide deacetylase-like 1 | NM_178772 | 2.23E-03 | 1.93 |
| 10570837 | Slc20a2 | solute carrier family 20, member 2 | NM_011394 | 2.35E-03 | 2.08 |
| 10436392 | Cpox | coproporphyrinogen oxidase | NM_007757 | 2.37E-03 | 1.59 |
| 10361075 | Mfsd7b | major facilitator superfamily domain containing 7B | BC010797 | 2.43E-03 | 1.94 |
| 10384985 | Rhbdf1 | rhomboid family 1 (Drosophila) | NM_010117 | 2.64E-03 | 2.49 |
| 10582592 | Acta1 | actin, alpha 1, skeletal muscle | NM_009606 | 2.65E-03 | 1.55 |
| 10467739 | Avpi1 | arginine vasopressin-induced 1 | NM_027106 | 2.85E-03 | 2.22 |
| 10397153 | Acot4 | acyl-CoA thioesterase 4 | NM_134247 | 2.87E-03 | 1.67 |
| 10437222 | Hnrnpa3 | heterogeneous nuclear ribonucleoprotein A3 | NM_053263 | 2.94E-03 | 1.52 |
| 10492045 | Hnrnpa3 | heterogeneous nuclear ribonucleoprotein A3 | NM_053263 | 2.94E-03 | 2.65 |
| 10574023 | Mt2 | metallothionein 2 | NM_008630 | 3.12E-03 | 1.53 |
| 10450038 | Angptl4 | angiopoietin-like 4 | ENSMUST00000002360 | 3.14E-03 | 2.43 |
| 10386473 | Srebf1 | sterol regulatory element binding transcription factor 1 | NM_011480 | 3.30E-03 | 1.63 |
| 10593225 | Zbtb16 | zinc finger and BTB domain containing 16 | NM_001033324 | 3.63E-03 | 1.53 |
| 10531051 | Ugt2b36 | UDP glucuronosyltransferase 2 family, polypeptide B36 | NM_001029867 | 3.63E-03 | 1.58 |
| 10421172 | Slc25a37 | solute carrier family 25, member 37 | NM_026331 | 3.63E-03 | 2.87 |
| 10373330 | Rdh7 | retinol dehydrogenase 7 | NM_001150749 | 3.75E-03 | 1.72 |
| 10459604 | 4933403F05Rik | RIKEN cDNA 4933403F05 gene | NM_153794 | 3.93E-03 | 1.88 |
| 10416071 | Chrna2 | cholinergic receptor, nicotinic, alpha polypeptide 2 (neuronal) | NM_144803 | 4.06E-03 | 1.62 |
| 10457733 | B4galt6 | UDP-Gal:betaGlcNAc beta 1,4-galactosyltransferase, polypeptide 6 | NM_019737 | 4.21E-03 | 1.68 |
| 10463005 | Cyp2c55 | cytochrome P450, family 2, subfamily c, polypeptide 55 | NM_028089 | 4.37E-03 | 1.68 |
| 10410007 | Fbp1 | fructose bisphosphatase 1 | NM_019395 | 4.50E-03 | 1.60 |
| 10545255 | Rpia | ribose 5-phosphate isomerase A | NM_009075 | 4.80E-03 | 1.67 |
| 10534102 | Gusb | glucuronidase, beta | ENSMUST00000026613 | 4.90E-03 | 1.51 |
| 10470182 | Bmyc | brain expressed myelocytomatosis oncogene | NM_023326 | 5.01E-03 | 1.83 |
| 10417972 | Camk2g | calcium/calmodulin-dependent protein kinase II gamma | NM_178597 | 5.01E-03 | 8.54 |
| 10368881 | Armc2 | armadillo repeat containing 2 | NM_001034858 | 5.01E-03 | 2.04 |
| 10381416 | Rnd2 | Rho family GTPase 2 | NM_009708 | 5.43E-03 | 2.69 |
| 10508719 | Snora16a | small nucleolar RNA, H/ACA box 16A | NR_029412 | 5.50E-03 | 1.67 |
| 10553521 | Gas2 | growth arrest specific 2 | ENSMUST00000129604 | 5.63E-03 | 1.64 |
| 10595466 | Pgm3 | phosphoglucomutase 3 | NM_028352 | 5.63E-03 | 1.70 |
| 10603431 | Suv39h1 | suppressor of variegation 3-9 homolog 1 (Drosophila) | NM_011514 | 5.63E-03 | 1.56 |
| 10501208 | Gstm6 | glutathione S-transferase, mu 6 | NM_008184 | 5.83E-03 | 1.63 |
| 10347748 | Acsl3 | acyl-CoA synthetase long-chain family member 3 | NM_028817 | 6.33E-03 | 1.71 |
| 10447885 | Acat3 | acetyl-Coenzyme A acetyltransferase 3 | NM_153151 | 6.33E-03 | 1.64 |
| 10369932 | Susd2 | sushi domain containing 2 | NM_027890 | 6.38E-03 | 2.45 |
| 10492174 | Tm4sf4 | transmembrane 4 superfamily member 4 | NM_145539 | 6.72E-03 | 2.81 |
| 10358787 | Lamc2 | laminin, gamma 2 | NM_008485 | 6.78E-03 | 2.70 |
| 10493798 | S100a16 | S100 calcium binding protein A16 | ENSMUST00000098911 | 6.90E-03 | 2.95 |
| 10438478 | Abcc5 | ATP-binding cassette, sub-family C (CFTR/MRP), member 5 | NM_013790 | 6.93E-03 | 2.01 |
| 10422962 | Nadkd1 | NAD kinase domain containing 1 | NM_001085410 | 7.10E-03 | 1.59 |
| 10367041 | Rdh9 | retinol dehydrogenase 9 | NM_153133 | 7.16E-03 | 2.63 |
| 10359446 | AI848100 | expressed sequence AI848100 | NM_172645 | 7.25E-03 | 2.06 |
| 10450482 | --- | --- | ENSMUST00000082919 | 7.25E-03 | 2.72 |
| 10428070 | 9430069I07Rik | RIKEN cDNA 9430069I07 gene | NM_001256161 | 7.30E-03 | 2.26 |
| 10506188 | Pgm2 | phosphoglucomutase 2 | NM_028132 | 7.39E-03 | 1.64 |
| 10587799 | Plscr2 | phospholipid scramblase 2 | NM_001195084 | 7.57E-03 | 1.56 |
| 10503023 | Cth | cystathionase (cystathionine gamma-lyase) | NM_145953 | 7.85E-03 | 2.41 |
| 10461735 | Glyat | glycine-N-acyltransferase | ENSMUST00000044976 | 8.08E-03 | 1.78 |
| 10406564 | Acot12 | acyl-CoA thioesterase 12 | NM_028790 | 8.28E-03 | 4.39 |
| 10600593 | Hnrnpa3 | heterogeneous nuclear ribonucleoprotein A3 | NM_053263 | 8.54E-03 | 1.81 |
| 10531057 | Ugt2b5 | UDP glucuronosyltransferase 2 family, polypeptide B5 | NM_009467 | 8.68E-03 | 1.61 |
| 10488797 | Pxmp4 | peroxisomal membrane protein 4 | NM_021534 | 8.74E-03 | 2.95 |
| 10414269 | Bnip3 | BCL2/adenovirus E1B interacting protein 3 | NM_009760 | 8.95E-03 | 1.50 |
| 10377439 | Per1 | period homolog 1 (Drosophila) | NM_001159367 | 8.96E-03 | 1.62 |
| 10404439 | Serpinb9b | serine (or cysteine) peptidase inhibitor, clade B, member 9b | ENSMUST00000006392 | 9.08E-03 | 1.53 |
| 10363773 | Rhobtb1 | Rho-related BTB domain containing 1 | NM_001252636 | 9.27E-03 | 1.96 |
| 10475437 | Sord | sorbitol dehydrogenase | NM_146126 | 9.32E-03 | 1.88 |
| 10467979 | Scd1 | stearoyl-Coenzyme A desaturase 1 | NM_009127 | 9.51E-03 | 1.53 |
| 10360454 | Opn3 | opsin 3 | NM_010098 | 9.67E-03 | 1.99 |
| 10597592 | Acaa1b | acetyl-Coenzyme A acyltransferase 1B | NM_146230 | 9.70E-03 | 1.61 |
| 10522819 | Ugt2b35 | UDP glucuronosyltransferase 2 family, polypeptide B35 | NM_172881 | 9.83E-03 | 1.82 |
| 10397145 | Acot2 | acyl-CoA thioesterase 2 | NM_134188 | 9.91E-03 | 1.53 |
| 10352703 | Mfsd7b | major facilitator superfamily domain containing 7B | NM_001081259 | 1.02E-02 | 1.67 |
| 10559185 | Syt8 | synaptotagmin VIII | NM_018802 | 1.02E-02 | 1.96 |
| 10358894 | Sord | sorbitol dehydrogenase | NM_146126 | 1.03E-02 | 3.70 |
| 10362359 | Pebp1 | phosphatidylethanolamine binding protein 1 | ENSMUST00000036951 | 1.03E-02 | 1.61 |
| 10519886 | Sema3c | sema domain, immunoglobulin domain (Ig), short basic domain, secreted, (semaphorin) 3C | NM_013657 | 1.03E-02 | 1.92 |
| 10354649 | Pgap1 | post-GPI attachment to proteins 1 | NM_001163314 | 1.04E-02 | 1.98 |
| 10431014 | Vkorc1l1 | vitamin K epoxide reductase complex, subunit 1-like 1 | NM_027121 | 1.04E-02 | 2.15 |
| 10485170 | Cry2 | cryptochrome 2 (photolyase-like) | NM_009963 | 1.04E-02 | 1.62 |
| 10593473 | 1810046K07Rik | RIKEN cDNA 1810046K07 gene | NM_027217 | 1.04E-02 | 1.54 |
| 10445627 | 2310039H08Rik | RIKEN cDNA 2310039H08 gene | NM_025966 | 1.07E-02 | 1.71 |
| 10417759 | Ube2e2 | ubiquitin-conjugating enzyme E2E 2 | NM_144839 | 1.08E-02 | 1.78 |
| 10565873 | Ppme1 | protein phosphatase methylesterase 1 | NM_028292 | 1.08E-02 | 1.59 |
| 10597518 | Tgfbr2 | transforming growth factor, beta receptor II | ENSMUST00000061101 | 1.12E-02 | 3.12 |
| 10535938 | N4bp2l1 | NEDD4 binding protein 2-like 1 | NM_133898 | 1.14E-02 | 1.72 |
| 10344713 | Ahcy | S-adenosylhomocysteine hydrolase | NM_016661 | 1.17E-02 | 1.70 |
| 10425763 | Cyp2d9 | cytochrome P450, family 2, subfamily d, polypeptide 9 | BC061505 | 1.18E-02 | 2.91 |
| 10401418 | Acot4 | acyl-CoA thioesterase 4 | NM_134247 | 1.19E-02 | 3.09 |
| 10599972 | LOC100862477 // Hnrnpa3 | heterogeneous nuclear ribonucleoprotein A3-like // heterogeneous nuclear ribonucleoprotein A3 | ENSMUST00000111964 | 1.21E-02 | 3.90 |
| 10554938 | Rab30 | RAB30, member RAS oncogene family | NM_029494 | 1.22E-02 | 1.79 |
| 10516906 | Snora73b | small nucleolar RNA, H/ACA box 73b | NR_028513 | 1.23E-02 | 1.59 |
| 10499899 | Sprr1a | small proline-rich protein 1A | NM_009264 | 1.27E-02 | 1.57 |
| 10540122 | Slc6a6 | solute carrier family 6 (neurotransmitter transporter, taurine), member 6 | ENSMUST00000032185 | 1.29E-02 | 1.92 |
| 10529052 | Slc5a6 | solute carrier family 5 (sodium-dependent vitamin transporter), member 6 | NM_001177621 | 1.32E-02 | 1.98 |
| 10586166 | --- | --- | ENSMUST00000083818 | 1.32E-02 | 1.79 |
| 10393047 | Galk1 | galactokinase 1 | NM_016905 | 1.35E-02 | 2.00 |
| 10537169 | Akr1b7 | aldo-keto reductase family 1, member B7 | NM_009731 | 1.36E-02 | 1.76 |
| 10514520 | Cyp2j9 | cytochrome P450, family 2, subfamily j, polypeptide 9 | NM_028979 | 1.36E-02 | 1.70 |
| 10466439 | Pebp1 | phosphatidylethanolamine binding protein 1 | NM_018858 | 1.36E-02 | 2.34 |
| 10530130 | Rell1 | RELT-like 1 | NM_145923 | 1.37E-02 | 1.51 |
| 10476939 | Zfp937 | zinc finger protein 937 | NM_001142411 | 1.37E-02 | 1.70 |
| 10424662 | Psca | prostate stem cell antigen | NM_028216 | 1.38E-02 | 2.09 |
| 10402512 | Scarna13 | small Cajal body-specific RNA 1 | NR_028576 | 1.41E-02 | 1.69 |
| 10563099 | Snord35b | small nucleolar RNA, C/D box 35B | NR_000004 | 1.41E-02 | 1.86 |
| 10373452 | --- | --- | GENSCAN00000035267 | 1.42E-02 | 1.64 |
| 10506488 | Ppap2b | phosphatidic acid phosphatase type 2B | NM_080555 | 1.42E-02 | 1.52 |
| 10556701 | Acsm5 | acyl-CoA synthetase medium-chain family member 5 | ENSMUST00000066465 | 1.48E-02 | 1.51 |
| 10355464 | Pecr | peroxisomal trans-2-enoyl-CoA reductase | NM_023523 | 1.48E-02 | 2.56 |
| 10473008 | Hnrnpa3 | heterogeneous nuclear ribonucleoprotein A3 | NM_146130 | 1.48E-02 | 2.50 |
| 10423333 | Fam134b | family with sequence similarity 134, member B | NM_001034851 | 1.51E-02 | 2.37 |
| 10595145 | --- | --- | ENSMUST00000095071 | 1.51E-02 | 1.55 |
| 10568785 | Bnip3 | BCL2/adenovirus E1B interacting protein 3 | NM_009760 | 1.55E-02 | 1.98 |
| 10404071 | Trim38 | tripartite motif-containing 38 | NM_001029935 | 1.60E-02 | 1.57 |
| 10449666 | Tff1 | trefoil factor 1 | NM_009362 | 1.60E-02 | 1.63 |
| 10587266 | Gclc | glutamate-cysteine ligase, catalytic subunit | NM_010295 | 1.62E-02 | 2.30 |
| 10556242 | --- | --- | ENSMUST00000082666 | 1.64E-02 | 1.60 |
| 10363860 | Slc16a9 | solute carrier family 16 (monocarboxylic acid transporters), member 9 | NM_025807 | 1.68E-02 | 1.55 |
| 10574511 | Ces2b | carboxyesterase 2B | NM_198171 | 1.68E-02 | 1.67 |
| 10493820 | S100a6 | S100 calcium binding protein A6 (calcyclin) | NM_011313 | 1.68E-02 | 2.37 |
| 10439762 | Ahcy | S-adenosylhomocysteine hydrolase | NM_016661 | 1.86E-02 | 2.23 |
| 10426891 | Mettl7a1 | methyltransferase like 7A1 | NM_027334 | 1.90E-02 | 1.60 |
| 10551197 | Cyp2b10 | cytochrome P450, family 2, subfamily b, polypeptide 10 | NM_009999 | 1.96E-02 | 1.55 |
| 10401149 | Plek2 | pleckstrin 2 | NM_013738 | 2.03E-02 | 1.75 |
| 10445241 | Tnfrsf21 | tumor necrosis factor receptor superfamily, member 21 | NM_178589 | 2.03E-02 | 1.60 |
| 10587818 | Plscr4 | phospholipid scramblase 4 | NM_178711 | 2.06E-02 | 1.60 |
| 10505568 | Frmd3 | FERM domain containing 3 | NM_172869 | 2.09E-02 | 4.46 |
| 10367641 | Mthfd1l | methylenetetrahydrofolate dehydrogenase (NADP+ dependent) 1-like | NM_172308 | 2.13E-02 | 2.32 |
| 10600355 | Snora70 | small nucleolar RNA, H/ACA box 70 | NR_002899 | 2.16E-02 | 2.01 |
| 10371770 | Gas2l3 | growth arrest-specific 2 like 3 | NM_001033331 | 2.16E-02 | 1.71 |
| 10488816 | Ahcy | S-adenosylhomocysteine hydrolase | NM_016661 | 2.17E-02 | 1.60 |
| 10605055 | Haus7 | HAUS augmin-like complex, subunit 7 | NM_028633 | 2.21E-02 | 1.50 |
| 10453867 | Rbbp8 | retinoblastoma binding protein 8 | NM_001252495 | 2.25E-02 | 1.59 |
| 10574598 | Ces3a | carboxylesterase 3A | NM_198672 | 2.29E-02 | 4.25 |
| 10543017 | Pdk4 | pyruvate dehydrogenase kinase, isoenzyme 4 | NM_013743 | 2.31E-02 | 2.25 |
| 10503370 | Hnrnpa3 | heterogeneous nuclear ribonucleoprotein A3 | NM_146130 | 2.35E-02 | 1.66 |
| 10513918 | --- | --- | ENSMUST00000120048 | 2.40E-02 | 1.58 |
| 10408656 | Eci2 | enoyl-Coenzyme A delta isomerase 2 | NM_011868 | 2.43E-02 | 1.94 |
| 10438911 | Atp13a3 | ATPase type 13A3 | NM_001128096 | 2.43E-02 | 3.20 |
| 10528507 | Pus7 | pseudouridylate synthase 7 homolog (S. cerevisiae) | NM_178403 | 2.43E-02 | 1.56 |
| 10385814 | Leap2 | liver-expressed antimicrobial peptide 2 | NM_153069 | 2.46E-02 | 1.90 |
| 10603746 | Maob | monoamine oxidase B | NM_172778 | 2.47E-02 | 1.67 |
| 10587194 | Gnb5 | guanine nucleotide binding protein (G protein), beta 5 | ENSMUST00000076889 | 2.49E-02 | 1.53 |
| 10485624 | Prrg4 | proline rich Gla (G-carboxyglutamic acid) 4 (transmembrane) | NM_178695 | 2.53E-02 | 3.37 |
| 10550906 | Plaur | plasminogen activator, urokinase receptor | NM_011113 | 2.57E-02 | 1.77 |
| 10597833 | Sec22c | SEC22 vesicle trafficking protein homolog C (S. cerevisiae) | NM_178677 | 2.68E-02 | 2.38 |
| 10516908 | Snora73a | small nucleolar RNA, H/ACA box 73a | NR_028512 | 2.71E-02 | 2.26 |
| 10598863 | Rgn | regucalcin | NM_009060 | 2.72E-02 | 1.83 |
| 10387723 | 2810408A11Rik | RIKEN cDNA 2810408A11 gene | NM_027419 | 2.74E-02 | 2.05 |
| 10440037 | Nit2 | nitrilase family, member 2 | NM_023175 | 2.82E-02 | 1.56 |
| 10388834 | Slc13a2 | solute carrier family 13 (sodium-dependent dicarboxylate transporter), member 2 | NM_022411 | 2.89E-02 | 2.04 |
| 10429555 | 2010109I03Rik | RIKEN cDNA 2010109I03 gene | NM_025929 | 2.93E-02 | 1.70 |
| 10497590 | Mecom | MDS1 and EVI1 complex locus | ENSMUST00000108271 | 2.99E-02 | 1.74 |
| 10361065 | Mfsd7b | major facilitator superfamily domain containing 7B | NM_001081259 | 3.00E-02 | 1.91 |
| 10347232 | Xrcc5 | X-ray repair complementing defective repair in Chinese hamster cells 5 | NM_009533 | 3.01E-02 | 2.46 |
| 10404965 | Rnf144b | ring finger protein 144B | NM_146042 | 3.16E-02 | 1.83 |
| 10401296 | Slc8a3 | solute carrier family 8 (sodium/calcium exchanger), member 3 | NM_080440 | 3.17E-02 | 1.69 |
| 10593776 | Nrg4 | neuregulin 4 | NM_032002 | 3.45E-02 | 2.54 |
| 10404872 | --- | --- | ENSMUST00000054395 | 3.45E-02 | 2.19 |
| 10465831 | 5730408K05Rik | RIKEN cDNA 5730408K05 gene | NR_027866 | 3.49E-02 | 2.63 |
| 10538459 | Aqp1 | aquaporin 1 | NM_007472 | 3.50E-02 | 1.73 |
| 10504757 | BC005685 | cDNA sequence BC005685 | BC005685 | 3.57E-02 | 1.90 |
| 10513320 | Ptgr1 | prostaglandin reductase 1 | NM_025968 | 3.64E-02 | 1.51 |
| 10367624 | --- | --- | ENSMUST00000083437 | 3.65E-02 | 1.65 |
| 10507671 | Guca2a | guanylate cyclase activator 2a (guanylin) | NM_008190 | 3.70E-02 | 1.88 |
| 10425686 | Ccdc134 | coiled-coil domain containing 134 | NM_172428 | 3.76E-02 | 1.66 |
| 10497209 | Mrps28 | mitochondrial ribosomal protein S28 | NM_025434 | 3.80E-02 | 1.92 |
| 10438423 | Olfr165 | olfactory receptor 165 | NM_146466 | 3.84E-02 | 1.64 |
| 10535698 | Cyp3a44 | cytochrome P450, family 3, subfamily a, polypeptide 44 | NM_177380 | 3.84E-02 | 2.00 |
| 10362073 | Sgk1 | serum/glucocorticoid regulated kinase 1 | NM_001161845 | 3.90E-02 | 1.74 |
| 10514219 | Scarna8 | small Cajal body-specific RNA 8 | NR_028545 | 3.99E-02 | 1.51 |
| 10428827 | Tmem65 | transmembrane protein 65 | NM_175212 | 4.04E-02 | 1.61 |
| 10542335 | Gprc5a | G protein-coupled receptor, family C, group 5, member A | NM_181444 | 4.15E-02 | 1.58 |
| 10381387 | G6pc | glucose-6-phosphatase, catalytic | NM_008061 | 4.18E-02 | 1.75 |
| 10430866 | Cyp2d10 | cytochrome P450, family 2, subfamily d, polypeptide 10 | NM_010005 | 4.29E-02 | 2.07 |
| 10450363 | Snord52 | small nucleolar RNA, C/D box 52 | NR_028527 | 4.29E-02 | 1.70 |
| 10352000 | Kmo | kynurenine 3-monooxygenase (kynurenine 3-hydroxylase) | NM_133809 | 4.32E-02 | 2.20 |
| 10598721 | Rpl3 | ribosomal protein L3 | NM_013762 | 4.34E-02 | 1.65 |
| 10553403 | Htatip2 | HIV-1 tat interactive protein 2, homolog (human) | NM_001146049 | 4.52E-02 | 2.01 |
| 10560709 | Pvr | poliovirus receptor | NM_027514 | 4.74E-02 | 1.61 |

Supplementary Table 2: Functionally enriched DAVID 6.7 gene ontology terms for the **(A)** 249 upregulated (19 enriched terms) and **(B)** 272 downregulated probe sets (31 enriched terms) for the caloric-restricted subgroup with respect to the control subgroup. **(C)** UP_TISSUE by DAVID 6.7. The adjusted p-value of 0.05 was set as the threshold for statistical significance. The background set was defined as the list of 26,966 microarray probesets after filtering the unannotated and control microarray probesets.

**(A)** #Probe sets: The 19 enriched terms associated with the 249 differentially upregulated probe sets (caloric-restricted with respect to control)

| **Term** | **Count** | **%** | **Fold-**  **Enrichment** | **Benjamini** |
| --- | --- | --- | --- | --- |
| mmu00982: Drug metabolism | 16 | 6.99 | 13.40 | 2.42E-11 |
| mmu00980: Metabolism of xenobiotics by cytochrome P450 | 14 | 6.11 | 13.19 | 7.51E-10 |
| GO: 0055114~oxidation reduction | 31 | 13.54 | 4.04 | 7.52E-08 |
| mmu00480: Glutathione metabolism | 10 | 4.37 | 11.82 | 2.90E-06 |
| mmu00983: Drug metabolism | 8 | 3.49 | 10.72 | 1.51E-04 |
| mmu03320: PPAR signalling pathway | 9 | 3.93 | 7.33 | 4.17E-04 |
| GO: 0051186~cofactor metabolic process | 13 | 5.68 | 6.34 | 4.46E-04 |
| mmu01040: Biosynthesis of unsaturated fatty acids | 6 | 2.62 | 13.40 | 9.37E-04 |
| GO: 0006732~coenzyme metabolic process | 11 | 4.80 | 6.84 | 1.44E-03 |
| mmu00830: Retinol metabolism | 7 | 3.06 | 6.70 | 6.57E-03 |
| GO: 0006631~fatty acid metabolic process | 11 | 4.80 | 5.25 | 1.10E-02 |
| GO: 0006637~acyl-CoA metabolic process | 5 | 2.18 | 21.46 | 1.40E-02 |
| GO: 0019318~hexose metabolic process | 10 | 4.37 | 5.20 | 1.74E-02 |
| GO: 0006766~vitamin metabolic process | 7 | 3.06 | 8.97 | 1.95E-02 |
| mmu00040: Pentose and glucuronate interconversions | 4 | 1.75 | 15.07 | 1.96E-02 |
| mmu00500: Starch and sucrose metabolism | 5 | 2.18 | 9.13 | 2.08E-02 |
| GO: 0005996~monosaccharide metabolic process | 10 | 4.37 | 4.66 | 3.43E-02 |
| GO: 0046364~monosaccharide biosynthetic process | 5 | 2.18 | 14.30 | 3.96E-02 |
| mmu00520: Amino sugar and nucleotide sugar metabolism | 5 | 2.18 | 7.01 | 4.38E-02 |

**(B)** The in 31 enriched terms associated with the 272 differentially downregulated probe sets (caloric-restricted with respect to control)

| **Term** | **Count** | **%** | **Fold-**  **Enrichment** | **Benjamini** |
| --- | --- | --- | --- | --- |
| GO: 0006955~immune response | 35 | 14.06 | 6.70 | 9.96E-16 |
| GO: 0006952~defense response | 25 | 10.04 | 5.03 | 7.09E-08 |
| GO: 0050778~positive regulation of immune response | 13 | 5.22 | 8.78 | 9.98E-06 |
| GO: 0002684~positive regulation of immune system process | 15 | 6.02 | 6.49 | 2.08E-05 |
| GO: 0048584~positive regulation of response to stimulus | 14 | 5.62 | 6.72 | 3.50E-05 |
| GO: 0006954~inflammatory response | 15 | 6.02 | 5.88 | 4.73E-05 |
| GO: 0045087~innate immune response | 11 | 4.42 | 8.96 | 6.20E-05 |
| GO: 0009611~response to wounding | 17 | 6.83 | 4.27 | 3.19E-04 |
| GO: 0002460~adaptive immune response based on somatic recombination of immune receptors built from immunoglobulin superfamily domains | 9 | 3.61 | 9.84 | 4.29E-04 |
| GO: 0002250~adaptive immune response | 9 | 3.61 | 9.84 | 4.29E-04 |
| GO: 0016064~immunoglobulin-mediated immune response | 8 | 3.21 | 12.08 | 4.48E-04 |
| GO: 0019724~B cell–mediated immunity | 8 | 3.21 | 11.66 | 5.20E-04 |
| GO: 0002252~immune effector process | 10 | 4.02 | 7.29 | 8.58E-04 |
| GO: 0009617~response to bacterium | 11 | 4.42 | 6.22 | 9.40E-04 |
| GO: 0002449~lymphocyte-mediated immunity | 8 | 3.21 | 9.77 | 1.34E-03 |
| GO: 0002443~leukocyte-mediated immunity | 8 | 3.21 | 8.31 | 3.63E-03 |
| GO: 0009615~response to virus | 7 | 2.81 | 9.09 | 8.17E-03 |
| GO: 0002526~acute inflammatory response | 7 | 2.81 | 7.86 | 1.71E-02 |
| GO: 0002697~regulation of immune effector process | 7 | 2.81 | 7.46 | 2.04E-02 |
| GO: 0002455~humoral immune response mediated by circulating immunoglobulin | 5 | 2.01 | 14.84 | 2.07E-02 |
| GO: 0002821~positive regulation of adaptive immune response | 5 | 2.01 | 14.84 | 2.07E-02 |
| GO: 0002824~positive regulation of adaptive immune response based on somatic recombination of immune receptors built from immunoglobulin superfamily domains | 5 | 2.01 | 14.84 | 2.07E-02 |
| GO: 0002253~activation of immune response | 7 | 2.81 | 7.36 | 2.08E-02 |
| GO: 0048002~antigen processing and presentation of peptide antigen | 5 | 2.01 | 12.98 | 2.99E-02 |
| GO: 0002495~antigen processing and presentation of peptide antigen via MHC class II | 4 | 1.61 | 22.15 | 3.58E-02 |
| GO: 0019886~antigen processing and presentation of exogenous peptide antigen via MHC class II | 4 | 1.61 | 22.15 | 3.58E-02 |
| GO: 0002696~positive regulation of leukocyte activation | 7 | 2.81 | 6.46 | 3.61E-02 |
| GO: 0032490~detection of molecule of bacterial origin | 3 | 1.20 | 62.31 | 3.85E-02 |
| GO: 0050867~positive regulation of cell activation | 7 | 2.81 | 6.32 | 3.88E-02 |
| GO: 0002699~positive regulation of immune effector process | 5 | 2.01 | 11.23 | 4.21E-02 |
| GO: 0002504~antigen processing and presentation of peptide or polysaccharide antigen via MHC class II | 4 | 1.61 | 19.55 | 4.27E-02 |

**(C)** UP_TISSUE by DAVID 6.7

| Input: All 521 DEGs; 477 DAVID gene IDs found | | | | | |
| --- | --- | --- | --- | --- | --- |
| Gene Ontology Term | Count | % | Fold Enrichment | P-Value | Benjamini |
| Colon | 54 | 1.304 | 2.41 | 4.73E-09 | 7.09E-07 |
| Liver | 119 | 2.873 | 1.60 | 5.71E-08 | 4.28E-06 |
| Spleen | 46 | 1.111 | 2.10 | 3.83E-06 | 1.44E-04 |
| Kidney | 77 | 1.859 | 1.65 | 1.17E-05 | 3.51E-04 |
| Small intestine | 22 | 0.531 | 2.82 | 4.13E-05 | 1.03E-03 |
| Activated spleen | 31 | 0.748 | 2.23 | 7.02E-05 | 1.50E-03 |
| Bone marrow | 50 | 1.207 | 1.63 | 7.00E-04 | 1.30E-02 |
| Jejunal and colic lymph node | 3 | 0.072 | 46.53 | 1.36E-03 | 2.24E-02 |
| Input: 249 Up-regulated DEGs, 207 DAVID gene IDs found | | | | | |
| Liver | 81 | 39.13 | 2.33 | 2.05E-14 | 2.36E-12 |
| Kidney | 54 | 26.09 | 2.47 | 4.90E-10 | 2.82E-08 |
| Colon | 19 | 9.179 | 1.81 | 1.74E-02 | 3.95E-01 |
| Small intestine | 9 | 4.348 | 2.46 | 3.02E-02 | 5.06E-01 |
| Input: 272 downregulated DEGs; 236 DAVID IDs found | | | | | |
| Spleen | 38 | 16.1 | 3.25 | 4.12E-10 | 4.90E-08 |
| Colon | 35 | 14.83 | 2.93 | 3.22E-08 | 1.92E-06 |
| Activated spleen | 25 | 10.59 | 3.37 | 4.32E-07 | 1.71E-05 |
| Bone marrow | 36 | 15.25 | 2.20 | 1.39E-05 | 3.30E-04 |
| Jejunal and colic lymph node | 3 | 1.271 | 87.14 | 3.87E-04 | 7.65E-03 |
| Small intestine | 13 | 5.508 | 3.12 | 1.00E-03 | 1.69E-02 |
| Thymus | 44 | 18.64 | 1.54 | 3.6E-03 | 5.00E-02 |

Supplementary Table 3: Enriched MetaCore™ pathways for the **(A)** 249 upregulated probe sets and **(B)** 272 downregulated probe sets for the caloric-restricted subgroup with respect to the control subgroup.

**(A)** Pathways for the 249 differentially upregulated probe sets (caloric-restricted with respect to control)

| **Maps** | **Total** | **FDR** | **In Data** | **Network Objects from Active Data** |
| --- | --- | --- | --- | --- |
| Regulation of metabolism_Bile acids regulation of glucose and lipid metabolism via FXR | 37 | 3.09E-07 | 8 | PPAR-alpha, ME1, PPCKC, G6PT, SREBP1 precursor, SREBP1 (nuclear), F16P, SCD |
| Transcription_Sirtuin6 regulation and functions | 64 | 7.77E-07 | 9 | SREBP1 (Golgi membrane), CD36, PPCKC, G6PT, RBBP8 (CtIP), SREBP1 precursor, SREBP1 (nuclear), SCD, PDK4 |
| Glutathione metabolism/Rodent version | 70 | 1.17E-06 | 9 | MGST2, GCL cat, MGST, GSTM1, GSTA3, GSTM4, GSTM1 (rodent), GSTA5, GSTM5 |
| Glutathione metabolism | 64 | 6.52E-06 | 8 | MGST2, GCL cat, MGST, GSTM1, GSTA3, GSTM4, GSTA5, GSTM5 |
| Glutathione metabolism/Human version | 65 | 6.52E-06 | 8 | MGST2, GCL cat, MGST, GSTM1, GSTA3, GSTM4, GSTA5, GSTM5 |
| Adiponectin in pathogenesis of type 2 diabetes | 29 | 8.67E-06 | 6 | PPAR-alpha, PPCKC, G6PT, SREBP1 precursor, SREBP1 (nuclear), SCD |
| Retinol metabolism | 72 | 1.77E-03 | 6 | UGT2B7, ADHFE1, CYP2B6, AL1A7, AL1A1, CYP2C18 |
| Retinol metabolism/Rodent version | 67 | 1.04E-02 | 5 | UGT2B7, CYP2B6, AL1A7, AL1A1, CYP2C18 |
| Regulation of lipid metabolism_Regulation of lipid metabolism via LXR, NF-Y and SREBP | 38 | 1.04E-02 | 4 | SREBP1 (Golgi membrane), SREBP1 precursor, SREBP1 (nuclear), SCD |
| Regulation of metabolism_Role of Adiponectin in regulation of metabolism | 43 | 1.40E-02 | 4 | PPAR-alpha, PPCKC, G6PT, SREBP1 precursor |
| Regulation of lipid metabolism.Regulation of fatty acid synthase activity in hepatocytes | 19 | 1.40E-02 | 3 | SREBP1 (Golgi membrane), SREBP1 precursor, SREBP1 (nuclear) |
| Glycolysis and gluconeogenesis p. 1 | 46 | 1.62E-02 | 4 | PGMU, G6PT, DHSO, F16P |
| Fructose metabolism/Rodent version | 83 | 1.83E-02 | 5 | ALD1, PMM1, G6PT, DHSO, F16P |
| Oxidative stress.Role of Sirtuin1 and PGC1-alpha in activation of antioxidant defense system | 60 | 3.76E-02 | 4 | GCL cat, NQO1, GSTA5, GSTM5 |
| Galactose metabolism/Rodent version | 64 | 4.45E-02 | 4 | PGMU, ALD1, GALK1, G6PT |
| Pyruvate metabolism/Rodent version | 66 | 4.66E-02 | 4 | ALD1, PPCKC, ADHFE1, AL1A7 |

**(B)** Pathways for the 272 differentially downregulated probe sets (caloric-restricted with respect to control)

| **Maps** | **Total** | **FDR** | **In Data** | **Network Objects from Active Data** |
| --- | --- | --- | --- | --- |
| Immune response.Antiviral actions of Interferons | 52 | 4.22E-12 | 12 | CIITA, OAS1, iNOS, IRF9, IRF1, 2'-5'-oligoadenylate synthetase, STAT1, MxA, RNaseL, STAT2, OAS2, ISGF3 |
| Immune response.IFN alpha/beta signalling pathway | 24 | 2.29E-09 | 8 | IRF9, IRF1, ISG54, STAT1, STAT2, USP18, STAT1/STAT2, ISGF3 |
| Bacterial infections in CF airways | 48 | 2.66E-04 | 6 | iNOS, IRF1, TLR4, STAT1, TLR1, MD-2 |
| Immune response. Bacterial infections in normal airways | 49 | 2.66E-04 | 6 | iNOS, IRF1, TLR4, STAT1, TLR1, MD-2 |
| Immune response.Lectin-induced complement pathway | 49 | 3.46E-03 | 5 | C2b, MBL2, C2a, C2, Factor I |
| Development.Angiotensin signalling via STATs | 32 | 7.20E-03 | 4 | IRF9, STAT1, STAT2, ISGF3 |
| Immune response.TLR3 and TLR4 induce TICAM1-specific signalling pathway | 20 | 2.47E-02 | 3 | TLR4, RIPK3, MD-2 |
| PDE4 regulation of cyto/chemokine expression in inflammatory skin diseases | 50 | 3.06E-02 | 4 | iNOS, MIG, MxA, IP10 |
| Immune response.Classical complement pathway | 52 | 3.16E-02 | 4 | C2b, C2a, C2, Factor I |
| Immune response . IFN gamma signalling pathway | 54 | 3.27E-02 | 4 | IRF9, IRF1, STAT1, ISGF3 |
| Immune response. TLR2 and TLR4 signalling pathways | 57 | 3.29E-02 | 4 | iNOS, TLR4, TLR1, MD-2 |
| Immune response. Role of PKR in stress-induced antiviral cell response | 57 | 3.29E-02 | 4 | IRF1, TLR4, STAT1, BAFF(TNFSF13B) |
| Development .Prolactin receptor signalling | 58 | 3.29E-02 | 4 | OAS1, IRF1, STAT1, Prolactin receptor |
| Immune response. IL-10 signalling pathway | 62 | 3.91E-02 | 4 | CD23, iNOS, STAT1, Bcl-3 |

Supplementary Table 4: List of genes with a significantly enriched numbers of interactions (when compared with the genome-wide background interactions) for the **(A)** 521 differentially expressed probe sets, **(B)** 249 upregulated probe sets, and **(C)** 272 downregulated probe sets for the caloric-restricted subgroup with respect to the control subgroup.

| **Network object name** | ***Network object name in MetaBase™*** | |
| --- | --- | --- |
| Fold-change | *Fold-change after caloric restriction (only for adjusted p value <0.05); >0: upregulated; <0: downregulated* |  |
| R | *Number of network objects in the complete database or background list that interact with the chosen object* |  |
| N | *Total number of gene-based objects in the complete database or background list* |  |
| n | *Number of network objects in the activated dataset(s)* |  |
| Actual | *Number of network objects in the activated dataset(s) that interact with the chosen object* |  |
| Expected | *Mean value for hypergeometric distribution (n*R/N)* |  |
| Ratio | *Connectivity ratio (Actual/Expected)* |  |
| p-value | *Probability of having the given value of Actual or higher (or lower for negative z-score)* |  |
| FDR | *Benjamini–Hochberg-corrected p-values (calculated separately for each object type)* |  |

**(A)** The 521 probe sets differentially regulated after caloric restriction (mapped to n=465 gene objects)

| **Object Type** | **Network Object name** | **Fold-**  **change** | **R** | **N** | **Actual** | **Expected** | **Ratio** | ***P*-value** | **FDR** |
| --- | --- | --- | --- | --- | --- | --- | --- | --- | --- |
| Transcription factors | IRF8 |  | 311 | 21191 | 36 | 6.82 | 5.28 | 3.63E-16 | 7.00E-14 |
| Transcription factors | STAT1 | -1.66 | 565 | 21191 | 49 | 12.40 | 3.95 | 2.18E-16 | 7.00E-14 |
| Transcription factors | STAT2 | -1.51 | 149 | 21191 | 20 | 3.27 | 6.12 | 1.04E-10 | 1.33E-08 |
| Transcription factors | SP1 |  | 1734 | 21191 | 76 | 38.05 | 2.00 | 4.41E-09 | 4.25E-07 |
| Transcription factors | C/EBPbeta |  | 791 | 21191 | 44 | 17.36 | 2.54 | 1.70E-08 | 1.31E-06 |
| Transcription factors | IRF9 | -1.51 | 59 | 21191 | 11 | 1.30 | 8.50 | 5.47E-08 | 3.52E-06 |
| Transcription factors | LXR-alpha |  | 163 | 21191 | 17 | 3.58 | 4.75 | 1.20E-07 | 6.63E-06 |
| Transcription factors | IRF7 |  | 111 | 21191 | 14 | 2.44 | 5.75 | 1.49E-07 | 7.19E-06 |
| Transcription factors | IRF1 | -1.54 | 344 | 21191 | 25 | 7.55 | 3.31 | 1.94E-07 | 8.31E-06 |
| Transcription factors | HNF4-alpha |  | 625 | 21191 | 35 | 13.71 | 2.55 | 4.51E-07 | 1.74E-05 |
| Transcription factors | RelA (p65 NF-kB subunit) |  | 1287 | 21191 | 55 | 28.24 | 1.95 | 1.71E-06 | 6.01E-05 |
| Transcription factors | CAR |  | 69 | 21191 | 10 | 1.51 | 6.61 | 2.51E-06 | 8.08E-05 |
| Transcription factors | NRF2 |  | 275 | 21191 | 20 | 6.03 | 3.31 | 3.21E-06 | 8.33E-05 |
| Transcription factors | GCR-alpha |  | 1348 | 21191 | 56 | 29.58 | 1.89 | 3.24E-06 | 8.33E-05 |
| Transcription factors | NF-kB p50/p50 |  | 86 | 21191 | 11 | 1.89 | 5.83 | 2.81E-06 | 8.33E-05 |
| Transcription factors | USF1 |  | 257 | 21191 | 19 | 5.64 | 3.37 | 4.45E-06 | 1.07E-04 |
| Enzymes | CBP |  | 716 | 21191 | 39 | 15.71 | 2.48 | 1.98E-07 | 1.20E-04 |
| Enzymes | p300 |  | 1046 | 21191 | 49 | 22.95 | 2.14 | 5.04E-07 | 1.52E-04 |
| Transcription factors | c-Jun |  | 1216 | 21191 | 51 | 26.68 | 1.91 | 7.11E-06 | 1.61E-04 |
| Transcription factors | IRF2 |  | 133 | 21191 | 13 | 2.92 | 4.45 | 7.57E-06 | 1.62E-04 |
| Transcription factors | EGR1 |  | 566 | 21191 | 30 | 12.42 | 2.42 | 8.98E-06 | 1.83E-04 |
| Enzymes | IGTP |  | 5 | 21191 | 4 | 0.11 | 36.46 | 1.13E-06 | 2.27E-04 |
| Transcription factors | RXRA |  | 329 | 21191 | 21 | 7.22 | 2.91 | 1.38E-05 | 2.66E-04 |
| Transcription factors | HNF3-alpha |  | 267 | 21191 | 18 | 5.86 | 3.07 | 2.75E-05 | 5.06E-04 |
| Transcription factors | FOXO3A |  | 329 | 21191 | 20 | 7.22 | 2.77 | 4.40E-05 | 7.38E-04 |
| Transcription factors | USF2 |  | 179 | 21191 | 14 | 3.93 | 3.56 | 4.32E-05 | 7.38E-04 |
| Transcription factors | PPAR-alpha | 2.49 | 330 | 21191 | 20 | 7.24 | 2.76 | 4.59E-05 | 7.38E-04 |
| Enzymes | HDAC3 |  | 499 | 21191 | 28 | 10.95 | 2.56 | 6.28E-06 | 9.49E-04 |
| Transcription factors | TCF7L2 (TCF4) |  | 456 | 21191 | 24 | 10.01 | 2.40 | 7.88E-05 | 1.17E-03 |
| Transcription factors | PPAR-gamma |  | 574 | 21191 | 28 | 12.60 | 2.22 | 7.69E-05 | 1.17E-03 |
| Enzymes | OAS1 | -1.74 | 24 | 21191 | 6 | 0.53 | 11.39 | 1.04E-05 | 1.26E-03 |
| Transcription factors | Oct-1 |  | 236 | 21191 | 15 | 5.18 | 2.90 | 2.41E-04 | 3.45E-03 |
| Transcription factors | PXR |  | 98 | 21191 | 9 | 2.15 | 4.19 | 3.06E-04 | 4.21E-03 |
| Transcription factors | LXR-beta |  | 120 | 21191 | 10 | 2.63 | 3.80 | 3.17E-04 | 4.22E-03 |
| Transcription factors | NFYA |  | 197 | 21191 | 13 | 4.32 | 3.01 | 4.32E-04 | 5.55E-03 |
| Other | N-CoR |  | 344 | 21191 | 23 | 7.55 | 3.05 | 2.50E-06 | 5.81E-03 |
| Transcription factors | Androgen receptor |  | 1399 | 21191 | 50 | 30.70 | 1.63 | 4.67E-04 | 5.81E-03 |
| Transcription factors | FKHR |  | 396 | 21191 | 20 | 8.69 | 2.30 | 5.17E-04 | 6.05E-03 |
| Transcription factors | PEA3 |  | 105 | 21191 | 9 | 2.30 | 3.91 | 5.10E-04 | 6.05E-03 |
| Transcription factors | FXR |  | 231 | 21191 | 14 | 5.07 | 2.76 | 6.18E-04 | 7.02E-03 |
| Transcription factors | HNF3-beta |  | 345 | 21191 | 18 | 7.57 | 2.38 | 6.67E-04 | 7.36E-03 |
| Transcription factors | C/EBPalpha |  | 595 | 21191 | 26 | 13.06 | 1.99 | 7.37E-04 | 7.90E-03 |
| Transcription factors | ESR1 (nuclear) |  | 1923 | 21191 | 63 | 42.20 | 1.49 | 8.49E-04 | 8.85E-03 |
| Transcription factors | MIST1 |  | 35 | 21191 | 5 | 0.77 | 6.51 | 9.39E-04 | 9.29E-03 |
| Transcription factors | STAT6 |  | 241 | 21191 | 14 | 5.29 | 2.65 | 9.35E-04 | 9.29E-03 |
| Transcription factors | Bcl-3 | -1.53 | 94 | 21191 | 8 | 2.06 | 3.88 | 1.08E-03 | 1.05E-02 |
| Transcription factors | SHP |  | 142 | 21191 | 10 | 3.12 | 3.21 | 1.19E-03 | 1.12E-02 |
| Transcription factors | TWIST2 |  | 22 | 21191 | 4 | 0.48 | 8.29 | 1.22E-03 | 1.12E-02 |
| Transcription factors | Oct-2 |  | 57 | 21191 | 6 | 1.25 | 4.80 | 1.52E-03 | 1.37E-02 |
| Enzymes | BAL | -2.06 | 13 | 21191 | 4 | 0.29 | 14.02 | 1.40E-04 | 1.41E-02 |
| Transcription factors | p73 |  | 316 | 21191 | 16 | 6.93 | 2.31 | 1.77E-03 | 1.52E-02 |
| Transcription factors | CREB-H |  | 40 | 21191 | 5 | 0.88 | 5.70 | 1.74E-03 | 1.52E-02 |
| Other | ISG15 |  | 253 | 21191 | 18 | 5.55 | 3.24 | 1.34E-05 | 1.55E-02 |
| Other | NCOA1 (SRC1) |  | 313 | 21191 | 20 | 6.87 | 2.91 | 2.17E-05 | 1.68E-02 |
| Transcription factors | HIF1A |  | 881 | 21191 | 33 | 19.33 | 1.71 | 2.08E-03 | 1.71E-02 |
| Transcription factors | c-Fos |  | 674 | 21191 | 27 | 14.79 | 1.83 | 2.08E-03 | 1.71E-02 |
| Transcription factors | IRF3 |  | 130 | 21191 | 9 | 2.85 | 3.16 | 2.32E-03 | 1.86E-02 |
| Transcription factors | KLF15 | 1.60 | 43 | 21191 | 5 | 0.94 | 5.30 | 2.41E-03 | 1.90E-02 |
| Transcription factors | ROR-alpha |  | 157 | 21191 | 10 | 3.45 | 2.90 | 2.50E-03 | 1.93E-02 |
| Transcription factors | STAT3 |  | 929 | 21191 | 34 | 20.39 | 1.67 | 2.60E-03 | 1.94E-02 |
| Transcription factors | c-Rel (NF-kB subunit) |  | 269 | 21191 | 14 | 5.90 | 2.37 | 2.61E-03 | 1.94E-02 |
| Transcription factors | BMAL1 | -1.87 | 186 | 21191 | 11 | 4.08 | 2.70 | 2.78E-03 | 1.99E-02 |
| Transcription factors | RARalpha |  | 392 | 21191 | 18 | 8.60 | 2.09 | 2.75E-03 | 1.99E-02 |
| Transcription factors | DEC1 (Stra13) |  | 134 | 21191 | 9 | 2.94 | 3.06 | 2.84E-03 | 2.00E-02 |
| Transcription factors | SPT4 |  | 14 | 21191 | 3 | 0.31 | 9.77 | 3.19E-03 | 2.20E-02 |
| Transcription factors | HNF1-alpha |  | 318 | 21191 | 15 | 6.98 | 2.15 | 4.71E-03 | 3.19E-02 |
| Enzymes | PCAF |  | 329 | 21191 | 18 | 7.22 | 2.49 | 3.82E-04 | 3.30E-02 |
| Transcription factors | REV-ERBalpha |  | 123 | 21191 | 8 | 2.70 | 2.96 | 5.80E-03 | 3.86E-02 |
| Transcription factors | p53 |  | 1893 | 21191 | 58 | 41.54 | 1.40 | 5.93E-03 | 3.88E-02 |
| Receptors | CD36 | 1.86 | 77 | 21191 | 8 | 1.69 | 4.74 | 2.83E-04 | 3.94E-02 |
| Receptors | IFNAR2 |  | 27 | 21191 | 5 | 0.59 | 8.44 | 2.70E-04 | 3.94E-02 |
| Receptors | TLR2 |  | 68 | 21191 | 7 | 1.49 | 4.69 | 7.16E-04 | 3.98E-02 |
| Receptors | MD-2 | -1.89 | 19 | 21191 | 4 | 0.42 | 9.59 | 6.83E-04 | 3.98E-02 |
| Receptors | CD74 |  | 33 | 21191 | 5 | 0.72 | 6.91 | 7.12E-04 | 3.98E-02 |
| Enzymes | DNMT3A |  | 176 | 21191 | 12 | 3.86 | 3.11 | 5.31E-04 | 4.01E-02 |
| Transcription factors | CLOCK |  | 156 | 21191 | 9 | 3.42 | 2.63 | 7.65E-03 | 4.92E-02 |
| Transcription factors | JunB |  | 244 | 21191 | 12 | 5.35 | 2.24 | 7.93E-03 | 4.94E-02 |
| Transcription factors | PU.1 |  | 502 | 21191 | 20 | 11.02 | 1.82 | 7.94E-03 | 4.94E-02 |

**(B)** The 249 probe sets differentially upregulated after caloric restriction (mapped to n=225 gene objects)

| **Object Type** | **Network Object name** | **Fold-**  **change** | **R** | **N** | **Actual** | **Expected** | **Ratio** | ***P*-value** | **FDR** |
| --- | --- | --- | --- | --- | --- | --- | --- | --- | --- |
| Transcription factors | HNF4-alpha |  | 625 | 21191 | 27 | 6.64 | 4.07 | 6.79E-10 | 2.30E-07 |
| Transcription factors | SP1 |  | 1734 | 21191 | 46 | 18.41 | 2.50 | 5.52E-09 | 9.36E-07 |
| Transcription factors | LXR-alpha |  | 163 | 21191 | 13 | 1.73 | 7.51 | 2.14E-08 | 2.41E-06 |
| Transcription factors | CAR |  | 69 | 21191 | 9 | 0.73 | 12.28 | 4.77E-08 | 4.04E-06 |
| Transcription factors | NRF2 |  | 275 | 21191 | 15 | 2.92 | 5.14 | 2.72E-07 | 1.85E-05 |
| Transcription factors | C/EBPalpha |  | 595 | 21191 | 22 | 6.32 | 3.48 | 4.22E-07 | 2.38E-05 |
| Transcription factors | GCR-alpha |  | 1348 | 21191 | 35 | 14.31 | 2.45 | 8.24E-07 | 3.99E-05 |
| Transcription factors | C/EBPbeta |  | 791 | 21191 | 24 | 8.40 | 2.86 | 4.04E-06 | 1.71E-04 |
| Transcription factors | ESR1 (nuclear) |  | 1923 | 21191 | 42 | 20.42 | 2.06 | 5.17E-06 | 1.95E-04 |
| Transcription factors | HNF3-alpha |  | 267 | 21191 | 13 | 2.84 | 4.59 | 6.01E-06 | 2.04E-04 |
| Transcription factors | TCF7L2 (TCF4) |  | 456 | 21191 | 17 | 4.84 | 3.51 | 8.22E-06 | 2.53E-04 |
| Transcription factors | PXR |  | 98 | 21191 | 8 | 1.04 | 7.69 | 9.88E-06 | 2.79E-04 |
| Transcription factors | PPAR-gamma |  | 574 | 21191 | 19 | 6.10 | 3.12 | 1.30E-05 | 3.38E-04 |
| Transcription factors | USF1 |  | 257 | 21191 | 12 | 2.73 | 4.40 | 2.09E-05 | 5.05E-04 |
| Transcription factors | MIST1 |  | 35 | 21191 | 5 | 0.37 | 13.45 | 3.23E-05 | 6.85E-04 |
| Transcription factors | BMAL1 | -1.87 | 186 | 21191 | 10 | 1.98 | 5.06 | 3.13E-05 | 6.85E-04 |
| Transcription factors | FOXO3A |  | 329 | 21191 | 13 | 3.49 | 3.72 | 5.40E-05 | 1.08E-03 |
| Transcription factors | USF2 |  | 179 | 21191 | 9 | 1.90 | 4.74 | 1.30E-04 | 2.45E-03 |
| Transcription factors | c-Jun |  | 1216 | 21191 | 27 | 12.91 | 2.09 | 2.35E-04 | 3.79E-03 |
| Transcription factors | PPAR-alpha | 2.49 | 330 | 21191 | 12 | 3.50 | 3.43 | 2.26E-04 | 3.79E-03 |
| Transcription factors | SP3 |  | 538 | 21191 | 16 | 5.71 | 2.80 | 2.18E-04 | 3.79E-03 |
| Transcription factors | LXR-beta |  | 120 | 21191 | 7 | 1.27 | 5.49 | 2.99E-04 | 4.60E-03 |
| Transcription factors | HNF3-beta |  | 345 | 21191 | 12 | 3.66 | 3.28 | 3.39E-04 | 4.99E-03 |
| Transcription factors | Androgen receptor |  | 1399 | 21191 | 29 | 14.85 | 1.95 | 4.27E-04 | 5.78E-03 |
| Transcription factors | SREBP1 (nuclear) | 1.57 | 167 | 21191 | 8 | 1.77 | 4.51 | 4.26E-04 | 5.78E-03 |
| Transcription factors | RXRA |  | 329 | 21191 | 11 | 3.49 | 3.15 | 8.24E-04 | 1.03E-02 |
| Transcription factors | SHP |  | 142 | 21191 | 7 | 1.51 | 4.64 | 8.20E-04 | 1.03E-02 |
| Transcription factors | EGR1 |  | 566 | 21191 | 15 | 6.01 | 2.50 | 1.11E-03 | 1.24E-02 |
| Transcription factors | FKHR |  | 396 | 21191 | 12 | 4.21 | 2.85 | 1.13E-03 | 1.24E-02 |
| Transcription factors | KLF15 | 1.60 | 43 | 21191 | 4 | 0.46 | 8.76 | 1.10E-03 | 1.24E-02 |
| Transcription factors | RARalpha |  | 392 | 21191 | 12 | 4.16 | 2.88 | 1.04E-03 | 1.24E-02 |
| Transcription factors | HIF1A |  | 881 | 21191 | 20 | 9.35 | 2.14 | 1.19E-03 | 1.26E-02 |
| Transcription factors | NFYA |  | 197 | 21191 | 8 | 2.09 | 3.83 | 1.25E-03 | 1.28E-02 |
| Transcription factors | ROR-alpha |  | 157 | 21191 | 7 | 1.67 | 4.20 | 1.47E-03 | 1.47E-02 |
| Transcription factors | TWIST2 |  | 22 | 21191 | 3 | 0.23 | 12.84 | 1.57E-03 | 1.52E-02 |
| Transcription factors | AP-2A |  | 361 | 21191 | 11 | 3.83 | 2.87 | 1.73E-03 | 1.63E-02 |
| Other | N-CoR |  | 344 | 21191 | 14 | 3.65 | 3.83 | 2.01E-05 | 1.66E-02 |
| Other | NCOA1 (SRC1) |  | 313 | 21191 | 13 | 3.32 | 3.91 | 3.24E-05 | 1.66E-02 |
| Other | miR-33a-5p |  | 36 | 21191 | 5 | 0.38 | 13.08 | 3.72E-05 | 1.66E-02 |
| Other | APOB |  | 119 | 21191 | 8 | 1.26 | 6.33 | 4.07E-05 | 1.66E-02 |
| Transcription factors | BACH1 |  | 83 | 21191 | 5 | 0.88 | 5.67 | 1.91E-03 | 1.75E-02 |
| Transcription factors | TR4 |  | 84 | 21191 | 5 | 0.89 | 5.61 | 2.02E-03 | 1.76E-02 |
| Transcription factors | Pitx3 |  | 24 | 21191 | 3 | 0.25 | 11.77 | 2.03E-03 | 1.76E-02 |
| Transcription factors | HNF1-alpha |  | 318 | 21191 | 10 | 3.38 | 2.96 | 2.21E-03 | 1.87E-02 |
| Transcription factors | c-Fos |  | 674 | 21191 | 16 | 7.16 | 2.24 | 2.36E-03 | 1.95E-02 |
| Transcription factors | Oct-1 |  | 236 | 21191 | 8 | 2.51 | 3.19 | 3.83E-03 | 3.09E-02 |
| Transcription factors | JunB |  | 244 | 21191 | 8 | 2.59 | 3.09 | 4.67E-03 | 3.60E-02 |
| Transcription factors | PROX1 |  | 102 | 21191 | 5 | 1.08 | 4.62 | 4.67E-03 | 3.60E-02 |
| Transcription factors | c-Jun/c-Jun |  | 11 | 21191 | 2 | 0.12 | 17.12 | 5.80E-03 | 4.37E-02 |
| Transcription factors | CLOCK |  | 156 | 21191 | 6 | 1.66 | 3.62 | 6.51E-03 | 4.80E-02 |
| Transcription factors | p73 |  | 316 | 21191 | 9 | 3.36 | 2.68 | 6.85E-03 | 4.94E-02 |

**(C)** The 272 probe sets differentially downregulated after caloric restriction (mapped to n=241 gene objects)

| **Object Type** | **Network Object name** | **Fold-change** | **R** | **N** | **Actual** | **Expected** | **Ratio** | **p-value** | **FDR** |
| --- | --- | --- | --- | --- | --- | --- | --- | --- | --- |
| Transcription factors | STAT1 | -1.66 | 565 | 21191 | 43 | 6.43 | 6.69 | 2.67E-23 | 8.20E-21 |
| Transcription factors | IRF8 |  | 311 | 21191 | 30 | 3.54 | 8.48 | 2.57E-19 | 3.94E-17 |
| Transcription factors | STAT2 | -1.51 | 149 | 21191 | 17 | 1.70 | 10.03 | 1.28E-12 | 1.16E-10 |
| Transcription factors | IRF1 | -1.54 | 344 | 21191 | 24 | 3.91 | 6.14 | 1.51E-12 | 1.16E-10 |
| Transcription factors | IRF7 |  | 111 | 21191 | 13 | 1.26 | 10.30 | 4.32E-10 | 2.65E-08 |
| Transcription factors | IRF9 | -1.51 | 59 | 21191 | 10 | 0.67 | 14.90 | 1.16E-09 | 5.93E-08 |
| Transcription factors | IRF2 |  | 133 | 21191 | 12 | 1.51 | 7.93 | 4.06E-08 | 1.78E-06 |
| Transcription factors | NF-kB p50/p50 |  | 86 | 21191 | 10 | 0.98 | 10.22 | 4.99E-08 | 1.92E-06 |
| Enzymes | IGTP |  | 5 | 21191 | 4 | 0.06 | 70.34 | 8.09E-08 | 3.19E-05 |
| Transcription factors | RelA (p65 NF-kB subunit) |  | 1287 | 21191 | 32 | 14.64 | 2.19 | 2.63E-05 | 8.97E-04 |
| Enzymes | CBP |  | 716 | 21191 | 23 | 8.14 | 2.83 | 8.03E-06 | 1.06E-03 |
| Enzymes | OAS1 | -1.74 | 24 | 21191 | 5 | 0.27 | 18.32 | 6.50E-06 | 1.06E-03 |
| Enzymes | BAL | -2.06 | 13 | 21191 | 4 | 0.15 | 27.06 | 1.08E-05 | 1.06E-03 |
| Transcription factors | PU.1 |  | 502 | 21191 | 17 | 5.71 | 2.98 | 6.68E-05 | 2.05E-03 |
| Receptors | IFNAR2 |  | 27 | 21191 | 5 | 0.31 | 16.28 | 1.20E-05 | 2.85E-03 |
| Transcription factors | STAT6 |  | 241 | 21191 | 11 | 2.74 | 4.01 | 1.05E-04 | 2.92E-03 |
| Transcription factors | IRF3 |  | 130 | 21191 | 8 | 1.48 | 5.41 | 1.23E-04 | 3.14E-03 |
| Receptors | CD74 |  | 33 | 21191 | 5 | 0.38 | 13.32 | 3.34E-05 | 3.96E-03 |
| Proteases | BAP1 |  | 82 | 21191 | 7 | 0.93 | 7.51 | 4.15E-05 | 4.44E-03 |
| Other | RIG-G | -2.22 | 66 | 21191 | 7 | 0.75 | 9.33 | 9.93E-06 | 8.21E-03 |
| Other | IFIT1 | -3.36 | 15 | 21191 | 4 | 0.17 | 23.45 | 2.02E-05 | 8.21E-03 |
| Other | BBAP | -2.06 | 15 | 21191 | 4 | 0.17 | 23.45 | 2.02E-05 | 8.21E-03 |
| Other | IFI56 |  | 46 | 21191 | 6 | 0.52 | 11.47 | 1.30E-05 | 8.21E-03 |
| Ligands | IL-1 beta |  | 101 | 21191 | 7 | 1.15 | 6.09 | 1.57E-04 | 1.04E-02 |
| Ligands | Fibronectin |  | 297 | 21191 | 12 | 3.38 | 3.55 | 1.62E-04 | 1.04E-02 |
| Transcription factors | Bcl-6 |  | 286 | 21191 | 11 | 3.25 | 3.38 | 4.58E-04 | 1.08E-02 |
| Other | ISG15 |  | 253 | 21191 | 12 | 2.88 | 4.17 | 3.52E-05 | 1.14E-02 |
| Ligands | IL-12 alpha |  | 35 | 21191 | 4 | 0.40 | 10.05 | 6.48E-04 | 1.57E-02 |
| Ligands | Apo-2L(TNFSF10) | -2.06 | 57 | 21191 | 5 | 0.65 | 7.71 | 4.72E-04 | 1.57E-02 |
| Ligands | IL-30 |  | 16 | 21191 | 3 | 0.18 | 16.49 | 7.29E-04 | 1.57E-02 |
| Ligands | IL-12 beta |  | 59 | 21191 | 5 | 0.67 | 7.45 | 5.54E-04 | 1.57E-02 |
| Transcription factors | C/EBPbeta |  | 791 | 21191 | 20 | 9.00 | 2.22 | 7.53E-04 | 1.65E-02 |
| Other | Nod2 (CARD15) |  | 61 | 21191 | 6 | 0.69 | 8.65 | 6.69E-05 | 1.82E-02 |
| Transcription factors | NKRF |  | 39 | 21191 | 4 | 0.44 | 9.02 | 9.82E-04 | 2.01E-02 |
| Transcription factors | c-Rel (NF-kB subunit) |  | 269 | 21191 | 10 | 3.06 | 3.27 | 1.07E-03 | 2.03E-02 |
| Transcription factors | IRF4 |  | 474 | 21191 | 14 | 5.39 | 2.60 | 1.12E-03 | 2.03E-02 |
| Ligands | IP10 | -2.03 | 72 | 21191 | 5 | 0.82 | 6.11 | 1.37E-03 | 2.06E-02 |
| Ligands | IL-4 |  | 70 | 21191 | 5 | 0.80 | 6.28 | 1.21E-03 | 2.06E-02 |
| Ligands | IL-21 |  | 20 | 21191 | 3 | 0.23 | 13.19 | 1.44E-03 | 2.06E-02 |
| Receptors | LMIR4 |  | 3 | 21191 | 2 | 0.03 | 58.62 | 3.84E-04 | 2.27E-02 |
| Receptors | CD209b |  | 3 | 21191 | 2 | 0.03 | 58.62 | 3.84E-04 | 2.27E-02 |
| Ligands | IL-7 |  | 6 | 21191 | 2 | 0.07 | 29.31 | 1.88E-03 | 2.42E-02 |
| Other | GBP1 | -1.83 | 43 | 21191 | 5 | 0.49 | 10.22 | 1.24E-04 | 2.87E-02 |
| Enzymes | p300 |  | 1046 | 21191 | 25 | 11.90 | 2.10 | 3.84E-04 | 3.04E-02 |
| Other | c-IAP2 |  | 135 | 21191 | 8 | 1.54 | 5.21 | 1.60E-04 | 3.25E-02 |
| Receptors | TLR2 |  | 68 | 21191 | 5 | 0.77 | 6.47 | 1.06E-03 | 3.60E-02 |
| Receptors | CCR3 |  | 18 | 21191 | 3 | 0.20 | 14.65 | 1.05E-03 | 3.60E-02 |
| Receptors | IFNGR1 |  | 39 | 21191 | 4 | 0.44 | 9.02 | 9.82E-04 | 3.60E-02 |
| Transcription factors | EGR1 |  | 566 | 21191 | 15 | 6.44 | 2.33 | 2.19E-03 | 3.60E-02 |
| Transcription factors | STAT3 |  | 929 | 21191 | 21 | 10.57 | 1.99 | 2.23E-03 | 3.60E-02 |
| Receptors | MD-2 | -1.89 | 19 | 21191 | 3 | 0.22 | 13.88 | 1.23E-03 | 3.64E-02 |
| Ligands | WNT3 |  | 28 | 21191 | 3 | 0.32 | 9.42 | 3.86E-03 | 4.52E-02 |
| Other | CEACAM1 |  | 50 | 21191 | 5 | 0.57 | 8.79 | 2.55E-04 | 4.56E-02 |
| Other | ISG54 | -2.30 | 51 | 21191 | 5 | 0.58 | 8.62 | 2.80E-04 | 4.56E-02 |
| Transcription factors | FOXP3 |  | 365 | 21191 | 11 | 4.15 | 2.65 | 3.21E-03 | 4.92E-02 |
| Enzymes | Oas1b | -1.72 | 4 | 21191 | 2 | 0.05 | 43.96 | 7.61E-04 | 4.95E-02 |
| Enzymes | PARP-14 | -1.84 | 17 | 21191 | 3 | 0.19 | 15.52 | 8.78E-04 | 4.95E-02 |

Supplementary Table 5: List of “gene–gene pairwise interactions” identified using MetaCore™ network analysis. The algorithm used was “direct interactions”. A positive fold-change indicates the gene was upregulated in the caloric-restricted subgroup with respect to the control subgroup and vice versa. Of the 493 genes, 144 interacted with one or more other genes, resulting in the 239 “gene–gene interactions”, with 162 “gene–gene interactions” for activation of the target genes, 33 “gene–gene interactions” for inhibition of the target genes, and 44 “gene–gene interactions” having unknown effects on the target genes.

| **From: targeting gene** | | | **To: targeted gene** | | |  |
| --- | --- | --- | --- | --- | --- | --- |
| **Network Object** | **Affymetrix ID** | **Fold-**  **change** | **Network Object** | **Affymetrix ID** | **Fold-**  **change** | **MetaCore™-curated**  **expected**  **effect of interaction** |
| BMAL1 | 10556463 | -1.87 | C1orf51 | 10373452 10500272 | 5.25 | Activation |
| PPAR-alpha | 10425987 | 2.49 | PPCKC | 10479047 | 4.39 | Activation |
| SREBP1 (nuclear) | 10386473 | 1.57 | PPCKC | 10479047 | 4.39 | Inhibition |
| ATF-3 | 10361091 | -1.55 | PPCKC | 10479047 | 4.39 | Inhibition |
| ATF/CREB | 10460767 10361090 | -1.55 | PPCKC | 10479047 | 4.39 | Unspecified |
| PPARGC1 (PGC1-alpha) | 10529977 10529979 | -1.65 | PPCKC | 10479047 | 4.39 | Activation |
| BMAL1 | 10556463 | -1.87 | PPCKC | 10479047 | 4.39 | Activation |
| SREBP1 (nuclear) | 10386473 | 1.57 | ABCA1 | 10512949 | 3.90 | Inhibition |
| PPAR-alpha | 10425987 | 2.49 | VLDLR | 10462281 | 2.81 | Activation |
| STAT1 | 10346191 | -1.66 | PL scramblase 1 | 10356082 10587792 | 2.81 | Activation |
| PPAR-alpha | 10425987 | 2.49 | Acot1 | 10397148 | 2.79 | Activation |
| PPAR-alpha | 10425987 | 2.49 | SCD | 10467979 | 2.72 | Activation |
| KLF15 | 10540028 | 1.60 | SCD | 10467979 | 2.72 | Inhibition |
| SREBP1 precursor | 10386473 | 1.57 | SCD | 10467979 | 2.72 | Activation |
| SREBP1 (nuclear) | 10386473 | 1.57 | SCD | 10467979 | 2.72 | Activation |
| G-protein beta | 10587194 | 1.56 | Dopamine transporter | 10406050 | 2.70 | Inhibition |
| PPAR-alpha | 10425987 | 2.49 | PGAR | 10450038 | 2.69 | Activation |
| ELF3 | 10358027 | -1.51 | PGAR | 10450038 | 2.69 | Activation |
| SGK1 | 10362073 | 1.86 | NDRG1 | 10429140 | 2.65 | Unspecified |
| Evi-1 | 10497590 | 1.67 | ZNF145 | 10593225 | 2.63 | Activation |
| PPARGC1 (PGC1-alpha) | 10529977 10529979 | -1.65 | ZNF145 | 10593225 | 2.63 | Activation |
| IRF9 | 10415293 | -1.51 | PPAR-alpha | 10425987 | 2.49 | Activation |
| STAT2 | 10367224 | -1.51 | PPAR-alpha | 10425987 | 2.49 | Unspecified |
| Bcl-3 | 10560685 | -1.53 | PPAR-alpha | 10425987 | 2.49 | Activation |
| PPARGC1 (PGC1-alpha) | 10529977 10529979 | -1.65 | PPAR-alpha | 10425987 | 2.49 | Activation |
| STAT1 | 10346191 | -1.66 | PPAR-alpha | 10425987 | 2.49 | Inhibition |
| BMAL1 | 10556463 | -1.87 | PPAR-alpha | 10425987 | 2.49 | Activation |
| ZHX2 | 10424213 | -1.59 | MDR1 | 10519527 | 2.34 | Inhibition |
| miR-200b-3p | 10519268 | -2.00 | MDR1 | 10519527 | 2.34 | Inhibition |
| BMAL1 | 10556463 | -1.87 | NQO1 | 10581538 | 2.26 | Activation |
| Evi-1 | 10497590 | 1.67 | HYEP | 10360684 | 2.24 | Activation |
| KLF15 | 10540028 | 1.60 | PDK4 | 10543017 | 2.23 | Activation |
| Bcl-3 | 10560685 | -1.53 | PDK4 | 10543017 | 2.23 | Activation |
| PPARGC1 (PGC1-alpha) | 10529977 10529979 | -1.65 | PDK4 | 10543017 | 2.23 | Activation |
| SREBP1 (nuclear) | 10386473 | 1.57 | ACSL3 | 10347748 | 2.11 | Unspecified |
| CRY2 | 10485170 | 1.81 | PER1 | 10377439 | 2.10 | Activation |
| Suv39H1 | 10603431 | 1.65 | PER1 | 10377439 | 2.10 | Unspecified |
| E4BP4 | 10409278 | -1.69 | PER1 | 10377439 | 2.10 | Inhibition |
| BMAL1 | 10556463 | -1.87 | PER1 | 10377439 | 2.10 | Activation |
| ATF-3 | 10361091 | -1.55 | GCL cat | 10587266 | 2.04 | Unspecified |
| ATF/CREB | 10460767 10361091 | -1.55 | GCL cat | 10587266 | 2.04 | Activation |
| RBBP8 (CtIP) | 10453867 | 1.70 | TFF1 | 10449666 | 2.04 | Inhibition |
| Sno-N | 10491300 | -1.58 | TFF1 | 10449666 | 2.04 | Activation |
| PPARGC1 (PGC1-alpha) | 10529977 10529979 | -1.65 | MAOB | 10603746 | 2.01 | Activation |
| KLF15 | 10540028 | 1.60 | Metallothionein-II | 10574023 | 1.94 | Unspecified |
| E4BP4 | 10409278 | -1.69 | Neuregulin 4 | 10593776 | 1.90 | Unspecified |
| miR-200b-3p | 10519268 | -2.00 | hnRNP A3 | 10344741 10437222 10473008 10492045 10503370 10600593 | 1.88 | Inhibition |
| PPAR-alpha | 10425987 | 2.49 | SGK1 | 10362073 | 1.86 | Activation |
| PPAR-alpha | 10425987 | 2.49 | CD36 | 10528207 | 1.86 | Activation |
| SREBP1 precursor | 10386473 | 1.57 | CD36 | 10528207 | 1.86 | Activation |
| IRF1 | 10376060 | -1.54 | CD36 | 10528207 | 1.86 | Inhibition |
| ATF-3 | 10361091 | -1.55 | CD36 | 10528207 | 1.86 | Unspecified |
| HDL proteins | 10430166 | -1.66 | CD36 | 10528207 | 1.86 | Activation |
| SREBP1 (nuclear) | 10386473 | 1.57 | Rdh7 | 10373330 | 1.84 | Unspecified |
| TGM2 | 10489204 | -3.10 | Actin | 10582592 | 1.83 | Unspecified |
| E4BP4 | 10409278 | -1.69 | CRY2 | 10485170 | 1.81 | Unspecified |
| BMAL1 | 10556463 | -1.87 | CRY2 | 10485170 | 1.81 | Activation |
| SREBP1 (nuclear) | 10386473 | 1.57 | GAS2L3 | 10371770 | 1.81 | Unspecified |
| RRM2 | 10394978 | 4.02 | PLAU (UPA) | 10413047 | 1.78 | Inhibition |
| VLDLR | 10462281 | 2.81 | PLAU (UPA) | 10413047 | 1.78 | Inhibition |
| PLAUR (uPAR) | 10550906 | 1.55 | PLAU (UPA) | 10413047 | 1.78 | Activation |
| KLF15 | 10540028 | 1.60 | SLC16A9 | 10363860 | 1.76 | Activation |
| Evi-1 | 10497590 | 1.67 | LAMC2 | 10358787 | 1.75 | Unspecified |
| WRN | 10578071 | -1.59 | CYP2B6 | 10551197 | 1.75 | Activation |
| PVR | 10560709 | 1.50 | CD96 | 10439762 | 1.68 | Activation |
| PPAR-alpha | 10425987 | 2.49 | DR6(TNFRSF21) | 10445241 | 1.67 | Activation |
| ATF-3 | 10361091 | -1.55 | NIP3 | 10568785 | 1.66 | Activation |
| Evi-1 | 10497590 | 1.67 | Suv39H1 | 10603431 | 1.65 | Activation |
| ELF3 | 10358027 | -1.51 | TGF-beta receptor type II | 10597518 | 1.64 | Activation |
| BMAL1 | 10556463 | -1.87 | KLF15 | 10540028 | 1.60 | Activation |
| BMAL1 | 10556463 | -1.87 | FTHFSDC1 | 10367641 | 1.59 | Unspecified |
| Evi-1 | 10497590 | 1.67 | Ku80 | 10347232 | 1.58 | Unspecified |
| RhoG | 10566132 | -1.54 | Ku80 | 10347232 | 1.58 | Activation |
| PPAR-alpha | 10425987 | 2.49 | CPOX | 10436392 | 1.58 | Activation |
| PPARGC1 (PGC1-alpha) | 10529977 10529979 | -1.65 | CPOX | 10436392 | 1.58 | Unspecified |
| PPAR-alpha | 10425987 | 2.49 | SREBP1 precursor | 10386473 | 1.57 | Activation |
| SREBP1 (nuclear) |  | 1.57 | SREBP1 precursor | 10386473 | 1.57 | Activation |
| BIN1 (Amphiphysin II) | 10454580 | 1.56 | SREBP1 precursor | 10386473 | 1.57 | Activation |
| BMAL1 | 10556463 | -1.87 | SREBP1 precursor | 10386473 | 1.57 | Activation |
| PLAU (UPA) | 10413047 | 1.78 | PLAUR (uPAR) | 10550906 | 1.55 | Activation |
| Bcl-3 | 10560685 | -1.53 | PLAUR (uPAR) | 10550906 | 1.55 | Inhibition |
| CDC14a | 10501629 | -1.50 | ERK3 | 10594988 | 1.51 | Unspecified |
| PPAR-alpha | 10425987 | 2.49 | G6PT | 10381387 | 1.51 | Activation |
| SREBP1 (nuclear) | 10386473 | 1.57 | G6PT | 10381387 | 1.51 | Unspecified |
| ATF/CREB | 10460767 10361091 | -1.55 | G6PT | 10381387 | 1.51 | Unspecified |
| PPARGC1 (PGC1-alpha) | 10529977 10529979 | -1.65 | G6PT | 10381387 | 1.51 | Activation |
| BMAL1 | 10556463 | -1.87 | G6PT | 10381387 | 1.51 | Unspecified |
| E4BP4 | 10409278 | -1.69 | Prolactin receptor | 10423049 | -1.51 | Unspecified |
| OAS1 | 10533256 | -1.74 | Prolactin receptor | 10423049 | -1.51 | Inhibition |
| STAT2 | 10367224 | -1.51 | IRF9 | 10415293 | -1.51 | Activation |
| IRF1 | 10376060 | -1.54 | IRF9 | 10415293 | -1.51 | Unspecified |
| STAT1 | 10346191 | -1.66 | Zibra | 10415293 | -1.51 | Unspecified |
| STAT2 | 10367224 | -1.51 | TAP1 (PSF1) | 10444244 | -1.51 | Activation |
| IRF1 | 10376060 | -1.54 | TAP1 (PSF1) | 10444244 | -1.51 | Activation |
| ATF/CREB | 10460767 10361091 | -1.55 | TAP1 (PSF1) | 10444244 | -1.51 | Activation |
| STAT1 | 10346191 | -1.66 | TAP1 (PSF1) | 10444244 | -1.51 | Activation |
| SGK1 | 10362073 | 1.86 | STAT2 | 10367224 | -1.51 | Activation |
| IRF9 | 10415293 | -1.51 | STAT2 | 10367224 | -1.51 | Activation |
| STAT1 | 10346191 | -1.66 | STAT2 | 10367224 | -1.51 | Unspecified |
| STAT1 | 10346191 | -1.66 | Bcl-3 | 10560685 | -1.53 | Activation |
| STAT1 | 10346191 | -1.66 | TRIM5 | 10566333 | -1.53 | Activation |
| IRF9 | 10415293 | -1.51 | IRF1 | 10376060 | -1.54 | Activation |
| STAT2 | 10367224 | -1.51 | IRF1 | 10376060 | -1.54 | Activation |
| DNAPTP6 | 10346348 | -1.56 | IRF1 | 10376060 | -1.54 | Unspecified |
| STAT1/STAT2 | 10367224 10346191 | -1.66 | IRF1 | 10376060 | -1.54 | Activation |
| STAT1 | 10346191 | -1.66 | IRF1 | 10376060 | -1.54 | Activation |
| Annexin V | 10497817 | -1.80 | IRF1 | 10376060 | -1.54 | Unspecified |
| BAL | 10435457 10439268 | -2.06 | IRF1 | 10376060 | -1.54 | Inhibition |
| IRF1 | 10376060 | -1.54 | CTIP1 | 10374727 | -1.54 | Unspecified |
| PPARGC1 (PGC1-alpha) | 10529977 10529979 | -1.65 | ATF-3 | 10361091 | -1.55 | Activation |
| IRF1 | 10376060 | -1.54 | CIITA | 10433507 | -1.55 | Activation |
| STAT1 | 10346191 | -1.66 | CIITA | 10433507 | -1.55 | Activation |
| IRF1 | 10376060 | -1.54 | PSMB9 | 10450145 | -1.57 | Activation |
| STAT1 | 10346191 | -1.66 | PSMB9 | 10450145 | -1.57 | Activation |
| Evi-1 | 10497590 | 1.67 | Sno-N | 10491300 | -1.58 | Unspecified |
| CIITA | 10433507 | -1.55 | RING6 | 10444229 | -1.62 | Activation |
| PPAR-alpha | 10425987 | 2.49 | Caprin-2 | 10549473 | -1.62 | Activation |
| STAT1 | 10346191 | -1.66 | TGTP | 10385518 10385533 | -1.62 | Activation |
| STAT2 | 10367224 | -1.51 | MxA | 10437224 | -1.62 | Activation |
| IRF1 | 10376060 | -1.54 | MxA | 10437224 | -1.62 | Activation |
| STAT1 | 10346191 | -1.66 | MxA | 10437224 | -1.62 | Activation |
| STAT1 | 10346191 | -1.66 | K12 | 10394068 | -1.65 | Unspecified |
| PPAR-alpha | 10425987 | 2.49 | PPARGC1 (PGC1-alpha) | 10529977 10529979 | -1.65 | Activation |
| NQO1 | 10581538 | 2.26 | PPARGC1 (PGC1-alpha) | 10529977 10529979 | -1.65 | Activation |
| SREBP1 precursor | 10386473 | 1.57 | PPARGC1 (PGC1-alpha) | 10529977 10529979 | -1.65 | Activation |
| BMAL1 | 10556463 | -1.87 | PPARGC1 (PGC1-alpha) | 10529977 10529979 | -1.65 | Unspecified |
| ZBP1 | 10490150 | -2.72 | RIPK3 | 10420198 | -1.66 | Activation |
| ZNF145 | 10593225 | 2.63 | STAT1 | 10346191 | -1.66 | Activation |
| SGK1 | 10362073 | 1.86 | STAT1 | 10346191 | -1.66 | Activation |
| IRF9 | 10415293 | -1.51 | STAT1 | 10346191 | -1.66 | Activation |
| Bcl-3 | 10560685 | -1.53 | STAT1 | 10346191 | -1.66 | Inhibition |
| ATF-3 | 10361091 | -1.55 | STAT1 | 10346191 | -1.66 | Activation |
| BAL | 10435457 10439268 | -2.06 | STAT1 | 10346191 | -1.66 | Activation |
| USP18 | 10541307 | -2.55 | STAT1 | 10346191 | -1.66 | Inhibition |
| STAT1 | 10346191 | -1.66 | BAFF(TNFSF13B) | 10570018 | -1.67 | Activation |
| IRF1 | 10376060 | -1.54 | OAS2 | 10533198 | -1.67 | Activation |
| STAT1 | 10346191 | -1.66 | OAS2 | 10533198 | -1.67 | Activation |
| ISGF3 | 10367224 10415293 10346191 | -1.66 | OAS2 | 10533198 | -1.67 | Activation |
| PPAR-alpha | 10425987 | 2.49 | E4BP4 | 10409278 | -1.69 | Inhibition |
| BACE2 | 10437222 | 1.72 | PPT1 | 10507784 | -1.70 | Unspecified |
| SREBP1 (nuclear) | 10386473 | 1.57 | PPT1 | 10507784 | -1.70 | Activation |
| IRF1 | 10376060 | -1.54 | XAF1 | 10378068 | -1.71 | Activation |
| STAT1 | 10346191 | -1.66 | XAF1 | 10378068 | -1.71 | Activation |
| Evi-1 | 10497590 | 1.67 | p22-phox | 10582303 | -1.72 | Activation |
| STAT1 | 10346191 | -1.66 | p22-phox | 10582303 | -1.72 | Activation |
| STAT2 | 10367224 | -1.51 | Oas1b | 10525158 | -1.72 | Activation |
| STAT1 | 10346191 | -1.66 | Oas1b | 10525158 | -1.72 | Unspecified |
| E430004N04Rik | 10362359 | 1.62 | VISA | 10476276 | -1.72 | Activation |
| RIG-G | 10462618 | -2.22 | VISA | 10476276 | -1.72 | Activation |
| LGP2 | 10391207 | -2.34 | VISA | 10476276 | -1.72 | Inhibition |
| RKIP | 10362359 10466439 | 1.62 | OAS1 | 10533256 | -1.74 | Activation |
| IRF9 | 10415293 | -1.51 | OAS1 | 10533256 | -1.74 | Unspecified |
| STAT2 | 10367224 | -1.51 | OAS1 | 10533256 | -1.74 | Activation |
| IRF1 | 10376060 | -1.54 | OAS1 | 10533256 | -1.74 | Activation |
| STAT1 | 10346191 | -1.66 | OAS1 | 10533256 | -1.74 | Activation |
| ISGF3 | 10367224 10415293 10346191 | -1.66 | OAS1 | 10533256 | -1.74 | Activation |
| PPARGC1 (PGC1-alpha) | 10529977 10529979 | -1.65 | UDP | 10374236 | -1.76 | Activation |
| CD36 | 10528207 | 1.86 | TLR4 | 10505517 | -1.77 | Activation |
| CIRBP | 10364712 | 1.86 | TLR4 | 10505517 | -1.77 | Activation |
| ATF-3 | 10361091 | -1.55 | TLR4 | 10505517 | -1.77 | Inhibition |
| TLR1 | 10530145 | -1.82 | TLR4 | 10505517 | -1.77 | Inhibition |
| MD-2 | 10344966 | -1.89 | TLR4 | 10505517 | -1.77 | Activation |
| MD-1 | 10404606 | -2.04 | TLR4 | 10505517 | -1.77 | Inhibition |
| CaMK II gamma | 10417972 | 1.85 | MIG | 10531407 | -1.79 | Activation |
| STAT2 | 10367224 | -1.51 | MIG | 10531407 | -1.79 | Unspecified |
| STAT1 | 10346191 | -1.66 | MIG | 10531407 | -1.79 | Activation |
| Evi-1 | 10497590 | 1.67 | LBH | 10446763 | -1.80 | Activation |
| IRF1 | 10376060 | -1.54 | IL-18BP | 10566050 | -1.81 | Activation |
| STAT1 | 10346191 | -1.66 | IL-18BP | 10566050 | -1.81 | Activation |
| IRF9 | 10415293 | -1.51 | GBP1 | 10496555 | -1.83 | Activation |
| STAT2 | 10367224 | -1.51 | GBP1 | 10496555 | -1.83 | Activation |
| IRF1 | 10376060 | -1.54 | GBP1 | 10496555 | -1.83 | Activation |
| STAT1 | 10346191 | -1.66 | GBP1 | 10496555 | -1.83 | Activation |
| STAT1 | 10346191 | -1.66 | Caspase-4 | 10582997 | -1.87 | Activation |
| PER1 | 10377439 | 2.10 | BMAL1 | 10556463 | -1.87 | Inhibition |
| CRY2 | 10485170 | 1.81 | BMAL1 | 10556463 | -1.87 | Inhibition |
| PPARGC1 (PGC1-alpha) | 10529977 10529979 | -1.65 | BMAL1 | 10556463 | -1.87 | Activation |
| STAT1 | 10346191 | -1.66 | MD-2 | 10344966 | -1.89 | Activation |
| MD-1 | 10404606 | -2.04 | MD-2 | 10344966 | -1.89 | Inhibition |
| STAT1 | 10346191 | -1.66 | CD23 | 10576757 | -1.90 | Activation |
| PARP-14 | 10439249 | -1.84 | CD23 | 10576757 | -1.90 | Inhibition |
| IRF1 | 10376060 | -1.54 | ART2.2 | 10565994 | -1.90 | Unspecified |
| SGK1 | 10362073 | 1.86 | NHE3 | 10406176 | -1.91 | Activation |
| CaMK II gamma | 10417972 | 1.85 | NHE3 | 10406176 | -1.91 | Inhibition |
| PLAU (UPA) | 10413047 | 1.78 | PDGF-C | 10492689 | -1.96 | Activation |
| RNaseL | 10350742 | -1.74 | FZD7 | 10346607 | -1.96 | Inhibition |
| ATF-3 | 10361091 | -1.55 | LEC1 | 10502748 10502766 10502774 10502778 10502780 | -2.02 | Unspecified |
| CaMK II gamma | 10417972 | 1.85 | IP10 | 10531415 | -2.03 | Activation |
| IRF9 | 10415293 | -1.51 | IP10 | 10531415 | -2.03 | Unspecified |
| STAT2 | 10367224 | -1.51 | IP10 | 10531415 | -2.03 | Activation |
| Bcl-3 | 10560685 | -1.53 | IP10 | 10531415 | -2.03 | Inhibition |
| IRF1 | 10376060 | -1.54 | IP10 | 10531415 | -2.03 | Activation |
| STAT1 | 10346191 | -1.66 | IP10 | 10531415 | -2.03 | Activation |
| ISGF3 | 10367224 10415293 10346191 | -1.66 | IP10 | 10531415 | -2.03 | Activation |
| IRF1 | 10376060 | -1.54 | BAL | 10435457 10439268 | -2.06 | Activation |
| IRF1 | 10376060 | -1.54 | BBAP | 10439268 | -2.06 | Activation |
| STAT1 | 10346191 | -1.66 | BBAP | 10439268 | -2.06 | Activation |
| BBAP |  | -2.06 | BAL | 10435457 10439268 | -2.06 | Activation |
| IRF9 | 10415293 | -1.51 | Apo-2L(TNFSF10) | 10491091 | -2.06 | Activation |
| STAT2 | 10367224 | -1.51 | Apo-2L(TNFSF10) | 10491091 | -2.06 | Unspecified |
| IRF1 | 10376060 | -1.54 | Apo-2L(TNFSF10) | 10491091 | -2.06 | Activation |
| STAT1 | 10346191 | -1.66 | Apo-2L(TNFSF10) | 10491091 | -2.06 | Activation |
| ISGF3 | 10367224 10415293 10346191 | -1.66 | Apo-2L(TNFSF10) | 10491091 | -2.06 | Activation |
| E4BP4 | 10409278 | -1.69 | Apo-2L(TNFSF10) | 10491091 | -2.06 | Inhibition |
| CIITA | 10433507 | -1.55 | HLA-DRA1 | 10450161 | -2.08 | Activation |
| STAT1 | 10346191 | -1.66 | TSA-1 | 10424676 | -2.18 | Activation |
| IRF9 | 10415293 | -1.51 | RIG-G | 10462618 | -2.22 | Activation |
| STAT2 | 10367224 | -1.51 | RIG-G | 10462618 | -2.22 | Activation |
| IRF1 | 10376060 | -1.54 | RIG-G | 10462618 | -2.22 | Activation |
| STAT1 | 10346191 | -1.66 | RIG-G | 10462618 | -2.22 | Activation |
| STAT1 | 10346191 | -1.66 | BRK | 10490611 | -2.25 | Unspecified |
| STAT1 | 10346191 | -1.66 | Mucin 4 | 10435112 | -2.25 | Activation |
| PPAR-alpha | 10425987 | 2.49 | MBL2 | 10462473 | -2.27 | Activation |
| ZNF145 | 10593225 | 2.63 | ISG54 | 10462613 | -2.30 | Activation |
| IRF9 | 10415293 | -1.51 | ISG54 | 10462613 | -2.30 | Activation |
| STAT2 | 10367224 | -1.51 | ISG54 | 10462613 | -2.30 | Activation |
| IRF1 | 10376060 | -1.54 | ISG54 | 10462613 | -2.30 | Activation |
| STAT1 | 10346191 | -1.66 | ISG54 | 10462613 | -2.30 | Activation |
| ISGF3 | 10367224 10415293 10346191 | -1.66 | ISG54 | 10462613 | -2.30 | Activation |
| IRF1 | 10376060 | -1.54 | SP100 | 10347948 10356262 | -2.43 | Activation |
| STAT1 | 10346191 | -1.66 | SP100 | 10347948 10356262 | -2.43 | Activation |
| STAT2 | 10367224 | -1.51 | USP18 | 10541307 | -2.55 | Unspecified |
| STAT1 | 10346191 | -1.66 | USP18 | 10541307 | -2.55 | Activation |
| STAT1 | 10346191 | -1.66 | microRNA 147 | 10475517 | -2.62 | Activation |
| STAT1 | 10346191 | -1.66 | Sca-1 | 10429564 | -2.67 | Unspecified |
| STAT1 | 10346191 | -1.66 | DUOX2 | 10486956 | -2.92 | Activation |
| CaMK II gamma | 10417972 | 1.85 | iNOS | 10379228 | -2.92 | Activation |
| Actin | 10582592 | 1.83 | iNOS | 10379228 | -2.92 | Activation |
| ELF3 | 10358027 | -1.51 | iNOS | 10379228 | -2.92 | Activation |
| STAT2 | 10367224 | -1.51 | iNOS | 10379228 | -2.92 | Activation |
| Bcl-3 | 10560685 | -1.53 | iNOS | 10379228 | -2.92 | Activation |
| IRF1 | 10376060 | -1.54 | iNOS | 10379228 | -2.92 | Activation |
| STAT1 | 10346191 | -1.66 | iNOS | 10379228 | -2.92 | Activation |
| E4BP4 | 10409278 | -1.69 | iNOS | 10379228 | -2.92 | Unspecified |
| STAT2 | 10367224 | -1.51 | Ceb1 | 10538590 | -3.23 | Unspecified |
| IRF9 | 10415293 | -1.51 | IFIT1 | 10462623 | -3.36 | Activation |
| STAT2 | 10367224 | -1.51 | IFIT1 | 10462623 | -3.36 | Activation |
| STAT1 | 10346191 | -1.66 | IFIT1 | 10462623 | -3.36 | Activation |
| ACTA1 | 10582592 | 1.83 | DNase I | 10433241 | -3.77 | Inhibition |

Supplementary Table 6: DAPI staining of bacteria in mice faeces.

| Total bacteria population | | | | | |
| --- | --- | --- | --- | --- | --- |
| Sample ID | log_10_ CFU/g | Sample ID | log_10_ CFU/g | Sample ID | log_10_ CFU/g |
| Ad lib 1 | 8.60 (0.01)^a^ | AT CR1 | -^b^ | AT 1 | - |
| Ad lib 2 | 8.47 (0.01) | AT CR2 | - | AT 2 | - |
| Ad lib 3 | 8.80 (0.04) | AT CR3 | - | AT 3 | - |
| Ad lib 4 | 8.50 (0.04) | AT CR4 | - | AT 4 | - |
| Ad lib 5 | 8.45 (0.03) | AT CR5 | - | AT 5 | - |
| Ad lib 6 | 8.40 (0.10) | AT CR6 | - | AT 6 | - |
|  |  | AT CR7 | - | AT 7 | - |

^a^ Mean (standard error of the mean). ^b^ The population level was less than 10^6^ CFU/g faeces. n=3

Supplementary Table 7: Metabolite quantification in **(A)** small intestine content and **(B)** fecal water measured by 1H-NMR. Data are presented as mean ± SEM for area under the curve of NMR signal normalized to the *ad libitum* control group. *p*-values were calculated using ANOVA and post-tests were used to determine significance of mean difference between 2 groups. *: different from AL, &: different from antibiotics-treated (AT).

**(A)** Small intestine

| Metabolite | Chemical shift (ppm) | Ad lib | CR | AT | AT-CR | p-val |
| --- | --- | --- | --- | --- | --- | --- |
| Phenylalanine | 7.33 | 1±0.17 | 1.22±0.07 | 0.78±0.19 | 1.07±0.11 | 0.256 |
| Tyrosine | 6.9 | 1±0.2 | 1.18±0.07 | 0.8±0.16 | 0.91±0.13 | 0.355 |
| Inosine | 6.11 | 1±0.28 | 0.72±0.12 | 1.22±0.35 | 0.96±0.23 | 0.646 |
| α-glucose | 5.24 | 1±0.42 | 0.29±0.1 | 0.9±0.46 | 0.22±0.06 | 0.258 |
| β-glucose | 4.65 | 1±0.41 | 0.34±0.09 | 0.89±0.44 | 0.22±0.06 | 0.258 |
| Taurine (free) | 3.27 | 1±0.22 | 0.46±0.05 | 0.62±0.18 | 0.55±0.19 | 0.17 |
| Glycerophospho-choline | 3.23 | 1±0.31 | 0.17±0.03* | 0.48±0.12 | 0.13±0.04* | 0.007 |
| Phosphocholine | 3.23 | 1±0.26 | 0.32±0.04 | 0.67±0.21 | 0.42±0.16 | 0.091 |
| Choline | 3.21 | 1±0.22 | 0.52±0.06 | 0.7±0.17 | 0.62±0.17 | 0.238 |
| Lysine | 3.15 | 1±0.18 | 0.66±0.05 | 0.81±0.16 | 0.74±0.15 | 0.414 |
| Taurine (conjugated to bile acids) | 3.09 | 1±0.26 | 2.19±0.29 | 2.58±0.84 | 3.12±0.73 | 0.099 |
| Creatine | 3.04 | 1±0.23 | 0.69±0.09 | 0.99±0.32 | 1.09±0.39 | 0.787 |
| Succinate | 2.41 | 1±0.1 | 0.61±0.08 | 0.72±0.18 | 0.52±0.11 | 0.074 |
| Acetate | 1.92 | 1±0.16 | 0.75±0.05 | 0.58±0.12 | 0.68±0.14 | 0.111 |
| CA+TCA | 1.67 | 1±0.23 | 2.32±0.26* | 1.73±0.47 | 3.02±0.71* | 0.027 |
| Alanine | 1.48 | 1±0.13 | 0.78±0.09 | 0.65±0.05* | 0.55±0.04* | 0.006 |
| Lactate | 1.34 | 1±0.16 | 0.5±0.05 | 0.57±0.13 | 0.49±0.14* | 0.029 |
| TβMCA+βMCA | 1.04 | 1±0.21 | 1.21±0.04 | 3.33±1.09* | 2.63±0.56 | 0.045 |
| TCA+CA | 0.93 | 1±0.25 | 2.68±0.36* | 1.7±0.48 | 3.36±0.88* | 0.02 |
| CA | 0.73 | 1±0.35 | 2.99±0.53* | 0.84±0.24 | 1.61±0.33 | 0.001 |
| TCA | 0.73 | 1±0.29 | 2.82±0.44* | 2.05±0.65 | 4.07±1.11* | 0.024 |
| TβMCA | 0.7 | 1±0.29 | 1.08±0.11 | 5.18±1.84* | 3.68±0.91 | 0.025 |
| TUDCA | 0.68 | 1±0.3 | 1.96±0.26 | 0.85±0.31 | 0.87±0.22 | 0.03 |

**(B)** Feces

| Metabolite | Chemical shift (ppm) | Ad lib | CR | AT | AT-CR | p-val |
| --- | --- | --- | --- | --- | --- | --- |
| Phenylalanine | 7.43 | 1±0.04 | 0.84±0.06 | 0.91±0.08 | 1.09±0.09 | 0.096 |
| Tyrosine | 6.91 | 1±0.05 | 0.91±0.06 | 0.77±0.06 | 1.06±0.1 | 0.034 |
| 3-HPP | 6.87 | 1±0.16 | 1±0.16 | 0.21±0.01* | 0.28±0.02* | 7.00E-06 |
| Uracil | 5.81 | 1±0.1 | 1.03±0.12 | 0.05±0* | 0.05±0.02* | 2.10E-10 |
| Glucose | 3.25 | 1±0.12 | 0.59±0.05 | 0.12±0* | 0.15±0.04* | 5.67E-10 |
| Glycerophosphocholine | 3.21 | 1±0.45 | 0.53±0.09 | 0.27±0.01 | 0.24±0.01 | 0.091 |
| Choline | 3.2 | 1±0.12 | 1.04±0.25 | 1.61±0.2 | 1.93±0.53 | 0.131 |
| Taurine (conjugated to bile acids) | 3.08 | 1±0.37 | 1.44±0.4 | 30.89±3.11* | 11.53±2.07^*,$^ | 7.66E-12 |
| Creatinine | 3.04 | 1±0.28 | 0.76±0.18 | 2.81±0.28 | 8.58±1.09^*,$^ | 4.43E-10 |
| 5-aminovalerate | 3.01 | 1±0.28 | 0.51±0.12 | 0.18±0.02* | 0.29±0.1* | 0.005 |
| Trimethylamine | 2.9 | 1±0.11 | 0.56±0.12* | 0.13±0.01* | 0.21±0.02* | 1.03E-07 |
| Aspartate | 2.8 | 1±0.12 | 1.31±0.1 | 0.55±0.03* | 0.68±0.09 | 0.00001 |
| Dimethylamine | 2.74 | 1±0.15 | 3.76±0.83* | 0.8±0.04 | 0.87±0.09 | 0.00004 |
| Succinate | 2.41 | 1±0.91 | 0.1±0.02 | 0.02±0 | 0.03±0.01 | 0.362 |
| Glutamate | 2.36 | 1±0.06 | 0.55±0.07* | 0.29±0.01* | 0.31±0.02* | 4.11E-11 |
| N-acetyl groups | 2.06 | 1±0.21 | 1±0.07 | 0.26±0.01* | 0.27±0.01* | 9.53E-06 |
| Acetate | 1.92 | 1±0.14 | 0.72±0.11 | 0.01±0* | 0.02±0.01* | 2.90E-09 |
| Alanine | 1.49 | 1±0.18 | 1.05±0.24 | 1.65±0.25 | 2.62±0.25^*,$^ | 0.00007 |
| Lactate | 1.34 | 1±0.04 | 0.96±0.12 | 1.71±0.12* | 1.89±0.16* | 2.86E-06 |
| 2-ketoisovalerate | 1.13 | 1±0.24 | 0.54±0.08* | 0.05±0* | 0.06±0.01* | 0.000024 |
| Propionate | 1.07 | 1±0.13 | 0.61±0.11* | 0.01±0* | 0.01±0* | 2.62E-09 |
| Isoleucine | 1.02 | 1±0.07 | 0.78±0.05* | 0.44±0.05* | 0.48±0.06* | 1.92E-07 |
| Valine | 1 | 1±0.06 | 0.87±0.08 | 0.92±0.1 | 0.94±0.08 | 0.74 |
| Leucine | 0.96 | 1±0.04 | 0.82±0.04* | 0.69±0.06* | 0.79±0.05* | 0.001 |
| Butyrate | 0.9 | 1±0.25 | 0.24±0.03* | 0.03±0* | 0.03±0* | 1.316E-05 |
| Bile acids | 0.73 | 1±0.54 | 0.68±0.24 | 1.79±0.34 | 0.64±0.13^$^ | 0.095 |
