# Supplementary material for: Complementary intestinal mucosa and microbiota responses to caloric restriction
Source: Sci Rep. 2018 Jul 27;8:11338. doi: 10.1038/s41598-018-29815-7 (PMC6063912; doi:10.1038/s41598-018-29815-7)
Supplement: Supplementary file 2 — Supplementary figures [file 41598_2018_29815_MOESM2_ESM.pdf]

Figure S1

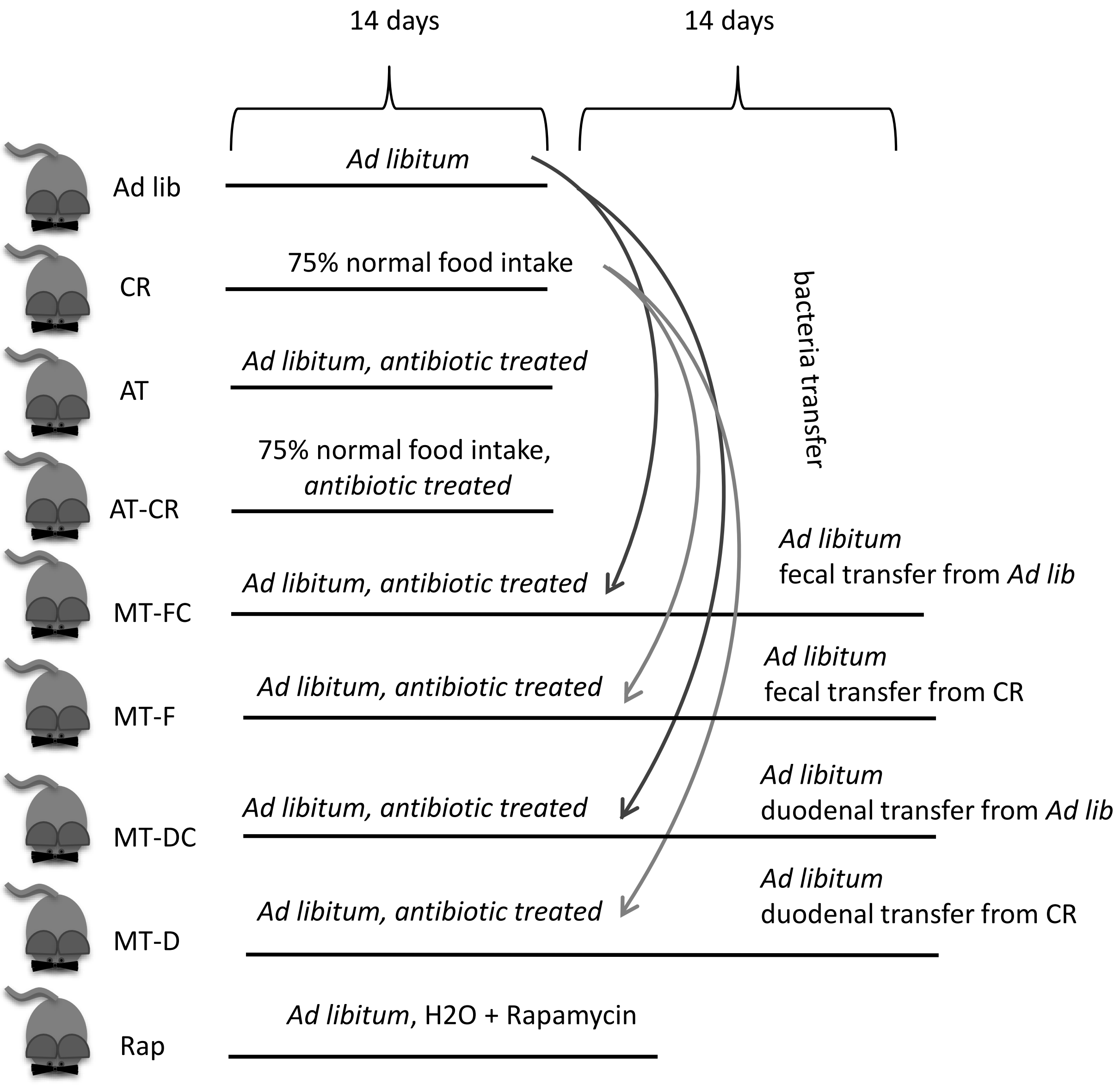
14 days
14 days
Ad lib
Ad libitum
CR
75% normal food intake
bacteria transfer
AT
Ad libitum, antibiotic treated
AT-CR
75% normal food intake, *antibiotic treated*
MT-FC
Ad libitum, antibiotic treated
Ad libitum
fecal transfer from *Ad lib*
MT-F
Ad libitum, antibiotic treated
Ad libitum
fecal transfer from CR
MT-DC
Ad libitum, antibiotic treated
Ad libitum
duodenal transfer from *Ad lib*
MT-D
Ad libitum, antibiotic treated
Ad libitum
duodenal transfer from CR
Rap
Ad libitum, H2O + Rapamycin

Figure S2

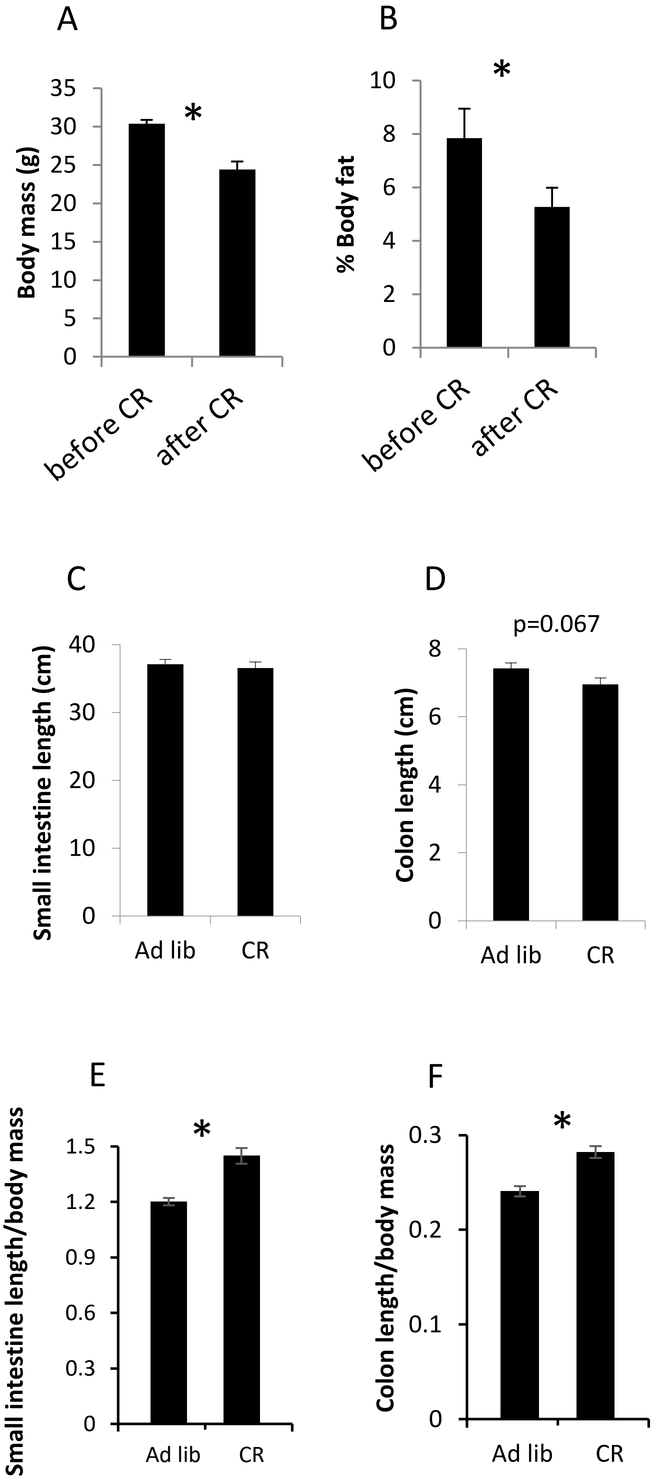
A
B
Body mass (g)
35
30
25
20
15
10
5
0
*
before CR
after CR
% Body fat
10
8
6
4
2
0
*
before CR
after CR
C
D
Small intestine length (cm)
40
30
20
10
0
Ad lib
CR
p=0.067
Colon length (cm)
8
6
4
2
0
Ad lib
CR
E
F
Small intestine length/body mass
1.5
1.2
0.9
0.6
0.3
0
*
Ad lib
CR
Colon length/body mass
0.3
0.2
0.1
0
*
Ad lib
CR

Figure S3

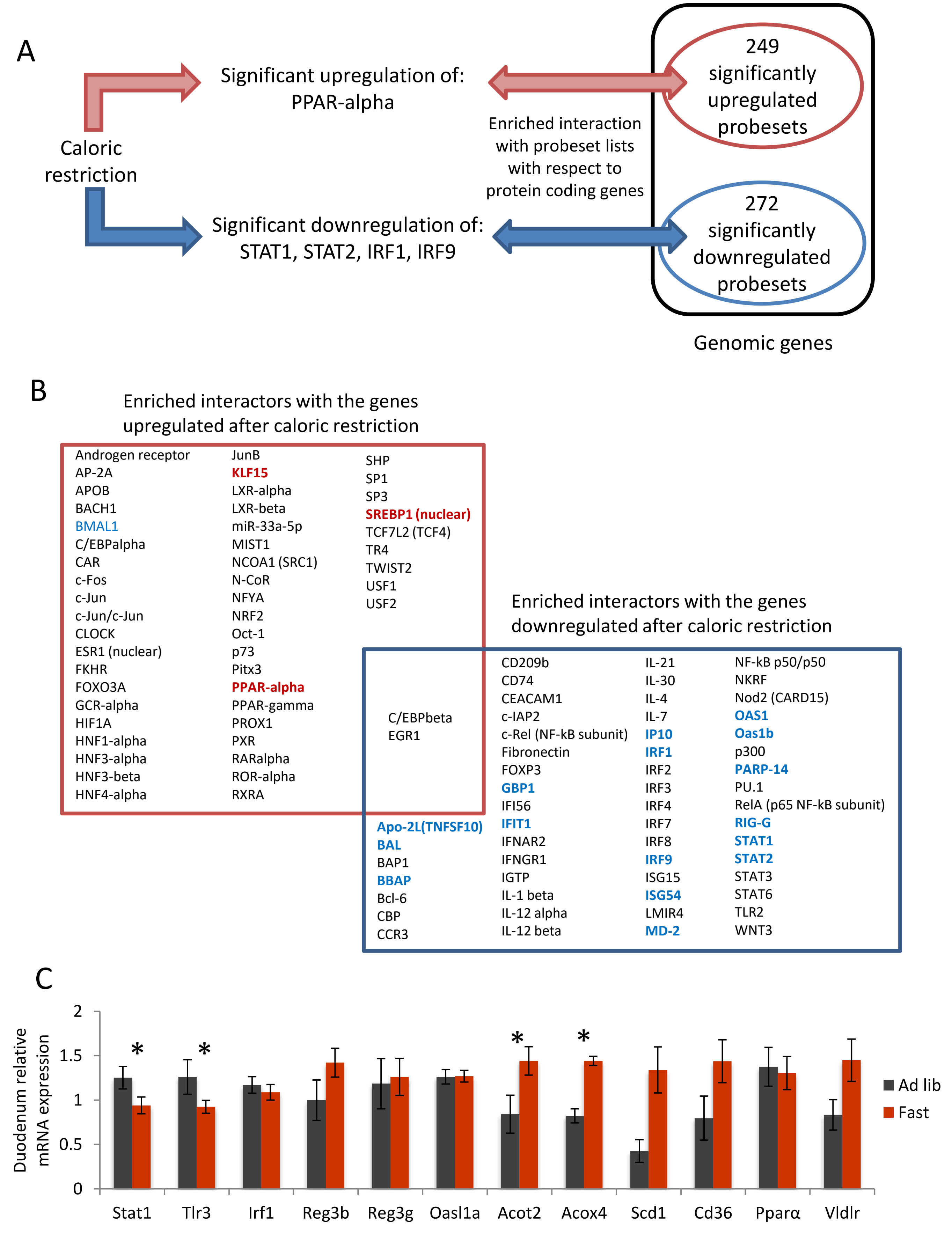
A
Caloric restriction
Significant upregulation of:
PPAR-alpha
Significant downregulation of:
STAT1, STAT2, IRF1, IRF9
Enriched interaction with probeset lists with respect to protein coding genes
249 significantly upregulated probesets
272 significantly downregulated probesets
Genomic genes
B
Enriched interactors with the genes upregulated after caloric restriction
Androgen receptor
AP-2A
APOB
BACH1
BMAL1
C/EBPalpha
CAR
c-Fos
c-Jun
c-Jun/c-Jun
CLOCK
ESR1 (nuclear)
FKHR
FOXO3A
GCR-alpha
HIF1A
HNF1-alpha
HNF3-alpha
HNF3-beta
HNF4-alpha
JunB
KLF15
LXR-alpha
LXR-beta
miR-33a-5p
MIST1
NCOA1 (SRC1)
N-CoR
NFYA
NRF2
Oct-1
p73
Pitx3
PPAR-alpha
PPAR-gamma
PROX1
PXR
RARalpha
ROR-alpha
RXRA
SHP
SP1
SP3
SREBP1 (nuclear)
TCF7L2 (TCF4)
TR4
TWIST2
USF1
USF2
C/EBPbeta
EGR1
Enriched interactors with the genes downregulated after caloric restriction
Apo-2L(TNFSF10)
BAL
BAP1
BBAP
Bcl-6
CBP
CCR3
CD209b
CD74
CEACAM1
c-IAP2
c-Rel (NF-kB subunit)
Fibronectin
FOXP3
GBP1
IFI56
IFIT1
IFNAR2
IFNGR1
IGTP
IL-1 beta
IL-12 alpha
IL-12 beta
IL-21
IL-30
IL-4
IL-7
IP10
IRF1
IRF2
IRF3
IRF4
IRF7
IRF8
IRF9
ISG15
ISG54
LMIR4
MD-2
NF-kB p50/p50
NKRF
Nod2 (CARD15)
OAS1
Oas1b
p300
PARP-14
PU.1
RelA (p65 NF-kB subunit)
RIG-G
STAT1
STAT2
STAT3
STAT6
TLR2
WNT3
C
Duodenum relative mRNA expression
0
0.5
1
1.5
2
*
*
*
*
Stat1
Tlr3
Irf1
Reg3b
Reg3g
Oasl1a
Acot2
Acox4
Scd1
Cd36
Pparα
Vldlr
Ad lib
Fast

Figure S4

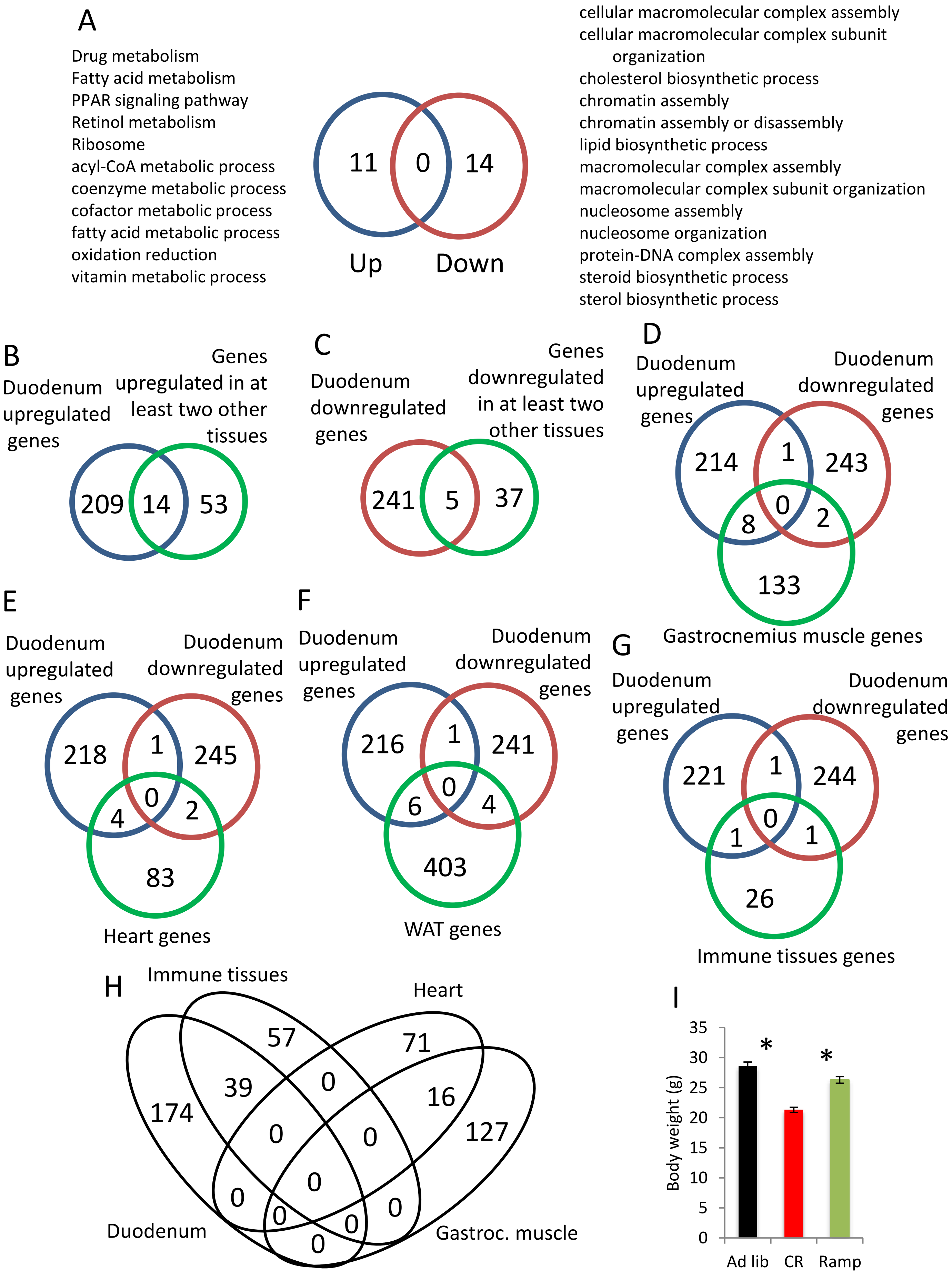
A
Drug metabolism
Fatty acid metabolism
PPAR signaling pathway
Retinol metabolism
Ribosome
acyl-CoA metabolic process
coenzyme metabolic process
cofactor metabolic process
fatty acid metabolic process
oxidation reduction
vitamin metabolic process
11
0
14
Up
Down
DNA packaging
cellular macromolecular complex assembly
cellular macromolecular complex subunit organization
cholesterol biosynthetic process
chromatin assembly
chromatin assembly or disassembly
lipid biosynthetic process
macromolecular complex assembly
macromolecular complex subunit organization
nucleosome assembly
nucleosome organization
protein-DNA complex assembly
steroid biosynthetic process
sterol biosynthetic process
B
Duodenum upregulated genes
Genes upregulated in at least two other tissues
209
14
53
C
Duodenum downregulated genes
Genes downregulated in at least two other tissues
241
5
37
D
Duodenum upregulated genes
Duodenum downregulated genes
214
1
243
8
0
2
133
Gastrocnemius muscle genes
E
Duodenum upregulated genes
Duodenum downregulated genes
218
1
245
4
0
2
83
Heart genes
F
Duodenum upregulated genes
Duodenum downregulated genes
216
1
241
6
0
4
403
WAT genes
G
Duodenum upregulated genes
Duodenum downregulated genes
221
1
244
1
0
1
26
Immune tissues genes
H
Immune tissues
Heart
Duodenum
Gastroc. muscle
57
71
39
0
16
174
0
0
127
0
0
0
0
0
0
I
Body weight (g)
35
30
25
20
15
10
5
0
*
*
Ad lib
CR
Ramp

Figure S5

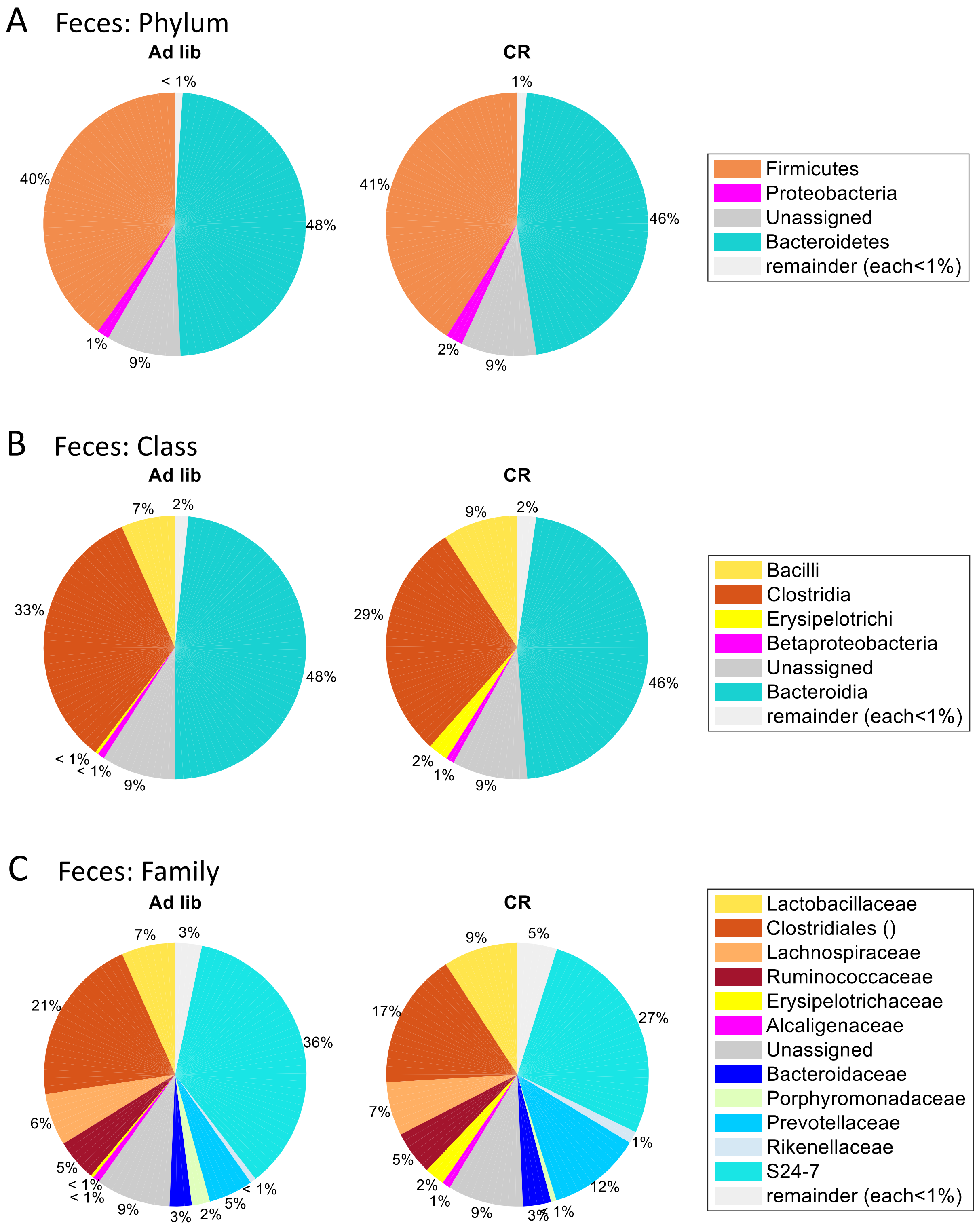
A Feces: Phylum
Ad lib
CR
< 1%
40%
48%
1%
9%
1%
41%
46%
2%
9%
Firmicutes
Proteobacteria
Unassigned
Bacteroidetes
remainder (each<1%)
B Feces: Class
Ad lib
CR
7%
2%
33%
48%
< 1%
< 1%
9%
9%
2%
29%
46%
2%
1%
9%
Bacilli
Clostridia
Erysipelotrichi
Betaproteobacteria
Unassigned
Bacteroidia
remainder (each<1%)
C Feces: Family
Ad lib
CR
7%
3%
21%
36%
6%
5%
< 1%
< 1%
9%
3%
2%
5%
< 1%
9%
5%
17%
27%
7%
5%
2%
1%
9%
3%
< 1%
1%
12%
1%
Lactobacillaceae
Clostridiales ()
Lachnospiraceae
Ruminococcaceae
Erysipelotrichaceae
Alcaligenaceae
Unassigned
Bacteroidaceae
Porphyromonadaceae
Prevotellaceae
Rikenellaceae
S24-7
remainder (each<1%)

Figure S6

D Small intestine: Phylum

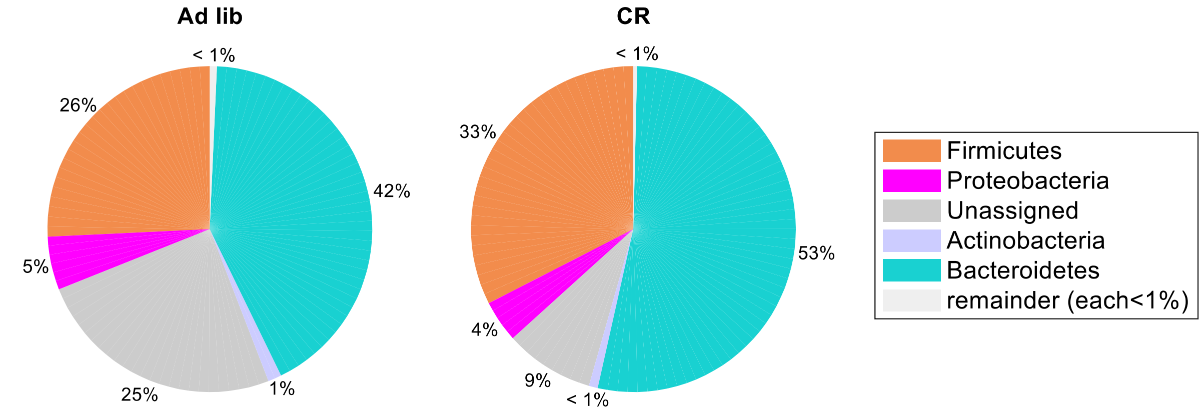

E Small intestine: Class

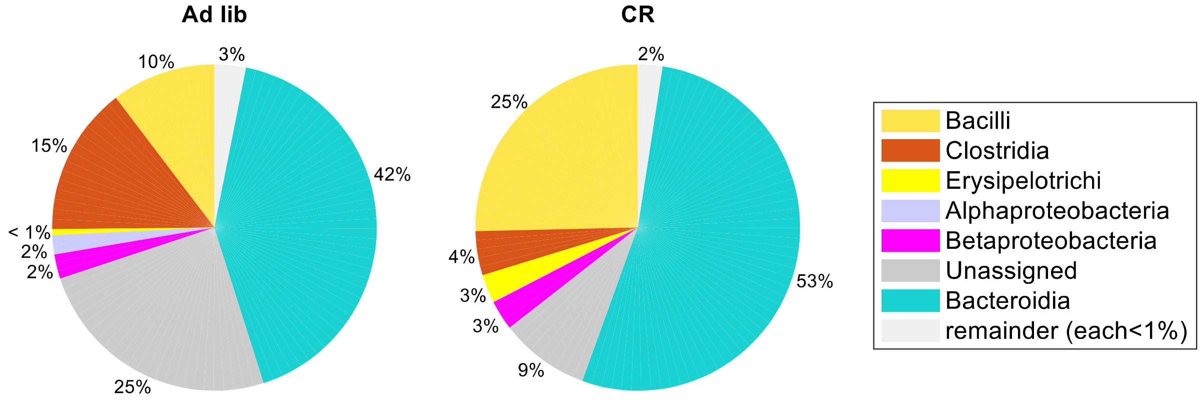

F Small intestine: Family

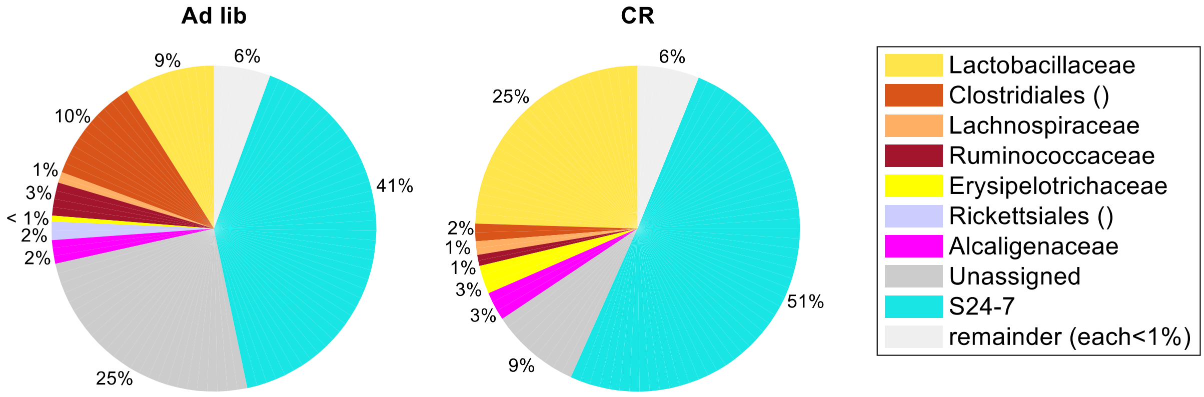

Figure S7

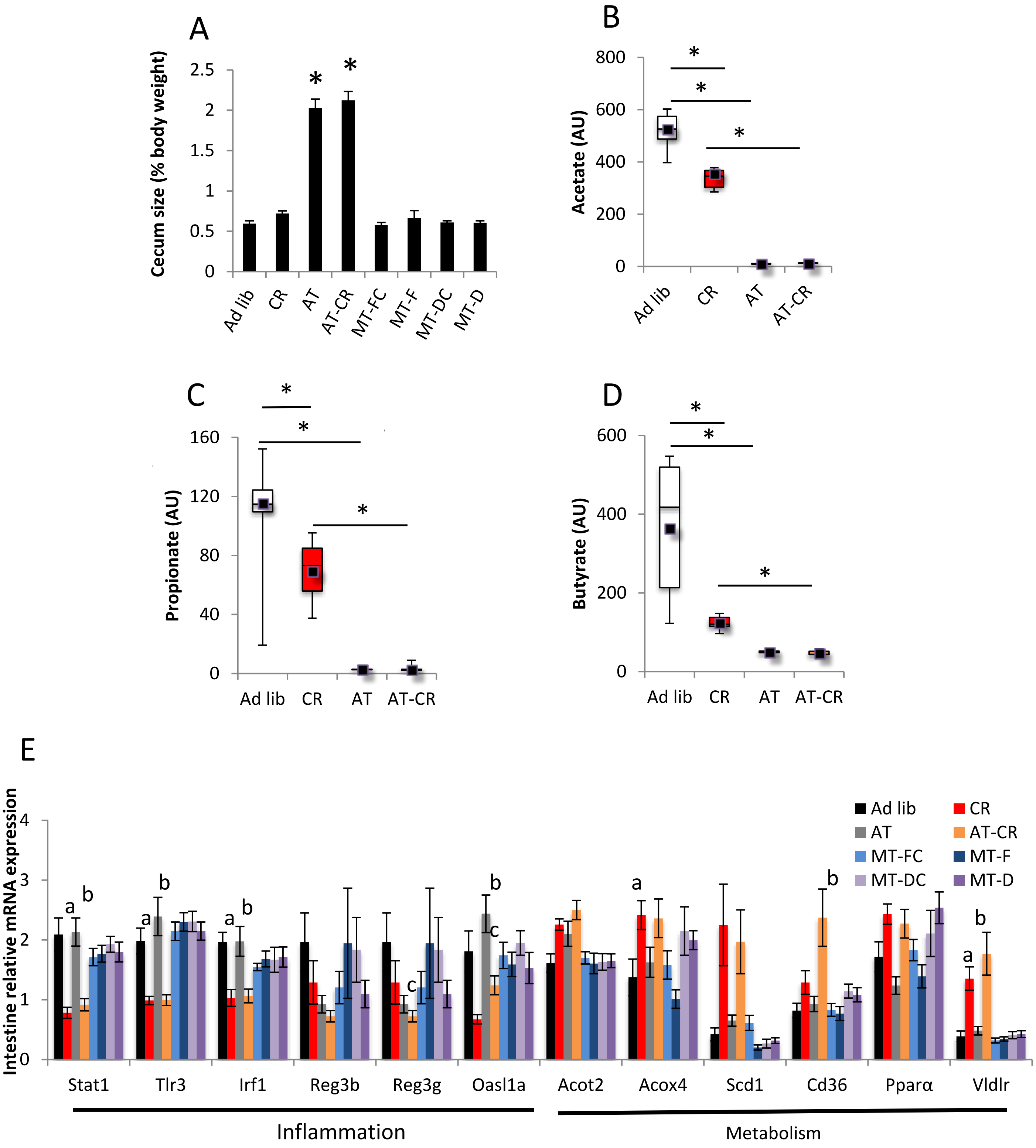
A
Cecum size (% body weight)
2.5
2
1.5
1
0.5
0
*
*
Ad lib
CR
AT
AT-CR
MT-FC
MT-F
MT-DC
MT-D
B
Acetate (AU)
800
600
400
200
0
*
*
*
Ad lib
CR
AT
AT-CR
C
Propionate (AU)
160
120
80
40
0
*
*
*
Ad lib
CR
AT
AT-CR
D
Butyrate (AU)
600
400
200
0
*
*
*
Ad lib
CR
AT
AT-CR
E
Intestine relative mRNA expression
4
3
2
1
0
Ad lib
CR
AT
AT-CR
MT-FC
MT-F
MT-DC
MT-D
a
b
c
Stat1
Tlr3
Irf1
Reg3b
Reg3g
Oasl1a
Acot2
Acox4
Scd1
Cd36
Pparα
Vldlr
Inflammation
Metabolism

Figure S8

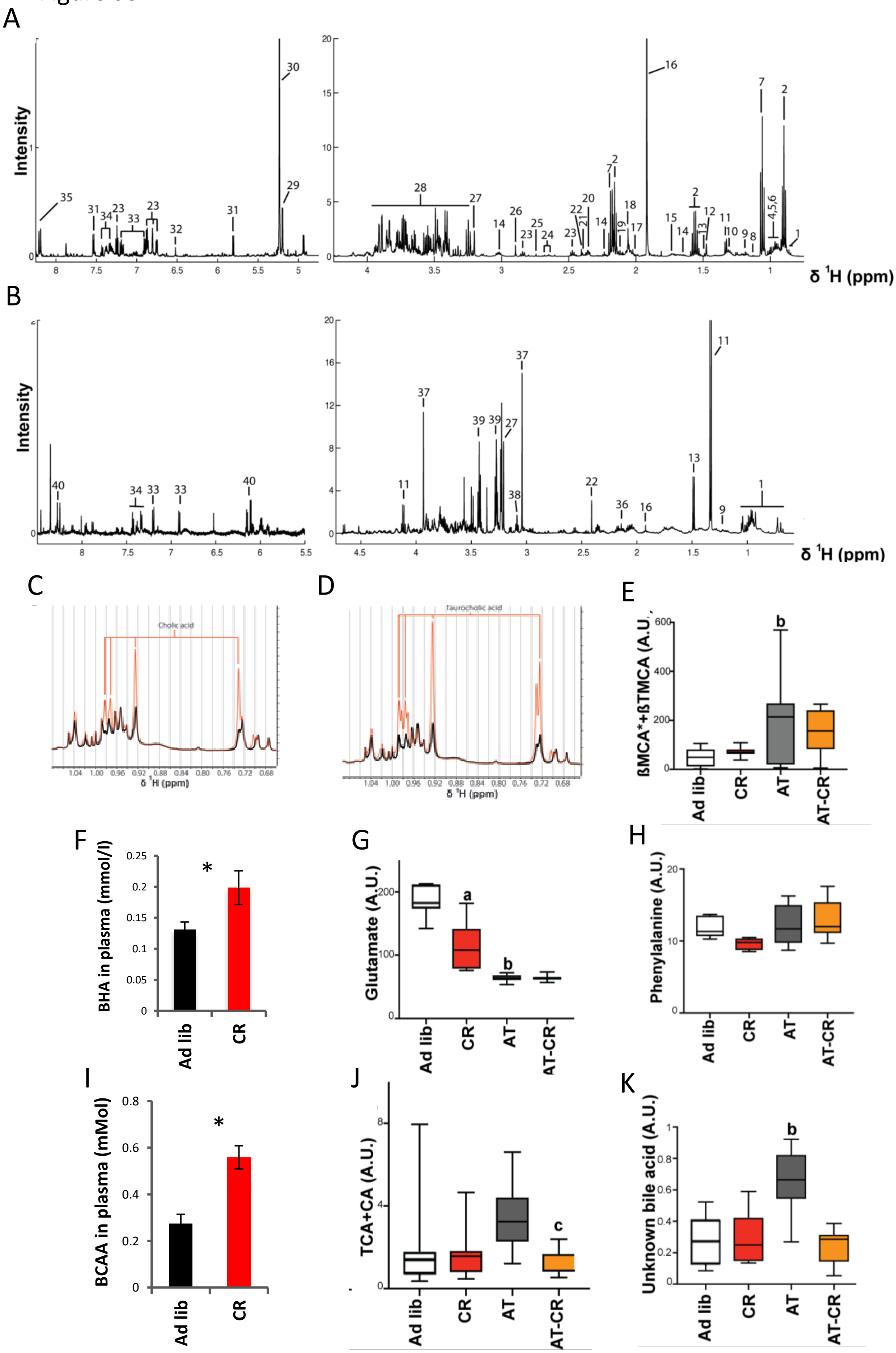

A
Intensity
δ ¹H (ppm)
B
Intensity
δ ¹H (ppm)
C
Cholic acid
δ ¹H (ppm)
D
Taurocholic acid
δ ¹H (ppm)
E
ßMCA*+ßTMCA (A.U.)
Ad lib
CR
AT
AT-CR
F
BHA in plasma (mmol/l)
Ad lib
CR
G
Glutamate (A.U.)
Ad lib
CR
AT
AT-CR
H
Phenylalanine (A.U.)
Ad lib
CR
AT
AT-CR
I
BCAA in plasma (mMol)
Ad lib
CR
J
TCA+CA (A.U.)
Ad lib
CR
AT
AT-CR
K
Unknown bile acid (A.U.)
Ad lib
CR
AT
AT-CR
