# Supplementary material for: Complementary intestinal mucosa and microbiota responses to caloric restriction
Source: Sci Rep. 2018 Jul 27;8:11338. doi: 10.1038/s41598-018-29815-7 (PMC6063912; doi:10.1038/s41598-018-29815-7)
Supplement: Supplementary file 3 — Supplementary information [file 41598_2018_29815_MOESM3_ESM.docx]

Complementary intestinal mucosa and microbiota responses to caloric restriction

Kalina Duszka, Sandrine Ellero-Simatos, Ghim Siong Ow, Marianne Defernez, Eeswari Paramalingam, Adrian Tett, Shi Ying, Jürgen König, Arjan Narbad, Vladimir A Kuznetsov, Hervé Guillou, Walter Wahli

**Supplementary mice experimental procedures**

**Animal care and experimental procedures**

Mice treated with antibiotics maintained the same body weight as the untreated mice (data not shown). Mice faeces were collected on days 12 and 13 of caloric restriction (CR) and snap frozen. Mice body composition was measured under anaesthesia before and after the CR period using an EchoMRI whole-body composition analyser (EchoMRI, Huston, TX, USA). Mice were euthanized using CO_2_, with blood drawn by cardiac puncture. Small intestine and colon scrapings which contain a collection of mucosal cells was collected ^1^. The scrapings as well as the contents of the small intestine were snap frozen and stored at − 80 °C until processed.

**qPCR**

RNA was isolated from intestinal scrapings using the RNeasy mini kit (Qiagen). Samples were thawed in lysis buffer, disrupted using a syringe and needle, and processed following the manufacturer’s recommendations. SuperScript® II Reverse Transcriptase (Invitrogen^TM^, Life Technologies) and random primers (Promega, Madison, WI, USA) were used for the reverse transcription step. Quantitative real-time PCR (qRT-PCR) reactions were carried out using the Applied Biosystems 7900HT (Life Technologies) with the SYBR green PCR Master Mix (Applied Biosystems, Life Technologies). The primers used are listed in the table below.

**RT-qPCR primer list:**

|  | Forward (5′-3′) | Reverse (5′-3′) |
| --- | --- | --- |
| Cd36 | TGATACTATGCCCGCCTCTCC | TTTCCCACACTCCTTTCTCCTCTA |
| Scd1 | GCCCACATGCTCCAAGAGAT | GGGCACTGTCTTCACCTTCT |
| Acot4 | TGCGGTACATGCTTCGACAT | TGGAAACTGTGGCTGAGACAT |
| Vldlr | GGTTACCAAGTATCTGTA | CATAGAAATATCTTCAAAGTG |
| Oas1a | ATGGAGCACGGACTCAGGA | TCACACACGACATTGACGGC |
| Stat1 | CAGTATGATGAGCACAGTA | AAGTCCTTCAGAGTAACAG |
| Tlr3 | GTATTGCCTGGTTTGTTAATTGG | AAGAGTTCAAAGGGGGCACT |
| Irf1 | CCCAGCTCTTGCTTTCGGA | AAGCCCAGTAGTTCACGACC |
| Reg3b | TGGGAATGGAGTAACAATG | GGCAACTTCACCTCACAT |
| Reg3g | TTCCTGTCCTCCATGATCAAAA | CATCCACCTCTGTTGGGTTCA |
| Acox2 | GCCTCATGCAATACTCTGGC | GGTACCAAGAACCTCTGTCCTG |
| Pparα | TACTGCCGTTTTCACAAGTGC | AGGTCGTGTTCACAGGTAAGA |
| Gpr43 | CCCTGTGCACATCCTCCTGC | GCGTTCCATGCTGATGCCCG |
| Gpr41 | TCCTGCCGTTTCGCATGGTGG | ACCGCCGTCAGGAAGAGGGAG |

**Functional enrichment analysis**

Gene ontology functional enrichment analyses were performed via DAVID Bioinformatics 6.7 ^2^. For the experimental data generated from our current work, the background set was defined as the subset of 26,966 probe sets from the Affymetrix MoGene 1.0 ST array after removal of control probe sets and probe sets that did not have annotation data from Affymetrix. Functional enrichment analyses were performed for gene sets defined in the categories GOTERM_BP_FAT and KEGG_PATHWAY. Functionally enriched DAVID terms were defined as those with a Benjamini-corrected *p*-value of less than 0.05.

For the analyses of the results, the background set was defined as the list of probe sets from the Affymetrix Mouse Genome 430 2 Array. Functional enrichment analyses were performed via DAVID Bioinformatics. Pathway enrichment analyses were also performed via MetaCore^TM^ (GeneGo Inc.), which is based on a database generated by manual curation of extensive literature references.

**Tissue specificity of genes differentially expressed after caloric restriction**

Tissue specificity of the proteins represented by CR mediated DEGs was analyzed using GO annotation dataset Gene Ontology Analysis (GOA) provided by the UniProt (UP) Consortium (GOA) ^3^. Using the UP_TISSUE tool in the DAVID Bioinformatics Resource 6.7, we specified the protein representing the DEGs in the mouse duodenum mucosa.

To assess the tissue differences in biological processes effected by CR, we tested whether the differentially expressed genes in duodenum were involved in the same biological processes as those differentially expressed in colon, hypothalamus, liver or muscle ^4,5^. The probesets that were identified by Selman *et al*. ^5^ as differentially expressed in colon, hypothalamus, liver and muscle were pooled together for gene ontology analysis as described earlier. The up and down-regulated genes in colon, liver, muscle or hypothalamus were found to be involved in distinct biological processes and pathways.

**Enrichment and significance of protein interactions**

We tested the null hypothesis that for any given gene in the genome, there was a difference in the number of interactions of that gene with the genes of interest as compared to the number of interactions of that same gene with all other genes in the genome. Hypergeometric tests were used to assess the fold enrichment and statistical significance. The *p*-values were adjusted using the Benjamini–Hochberg multivariate correction for each object type (e.g., transcription factors, enzymes, receptors, proteases, ligands, etc.) separately. A corrected *p*-value cut-off of 0.05 was used as the significance threshold. MetaCore^TM^ software (GeneGo Inc.) was used for the analysis.

**Network analysis**

MetaCore^TM^ was used for the network analysis of the 521 differentially regulated probe sets (adjusted *p*≤0.05). The probe set IDs were converted to a MetaCore^TM^ gene network object; although, several probe set IDs could not be mapped to MetaCore^TM^ gene network objects and several mapped to multiple network objects and vice versa. After mapping, there were 493 MetaCore^TM^ gene network objects that were used for building the network. The “direct interactions” algorithm was used with default parameters.

**Bioinformatics and statistics**

For mice experimental data (body weight, body composition, and lengths of the small intestine and colon) and qRT-PCR experiments, statistical analysis was performed using the two-sided student’s t-test or one-way ANOVA with a Bonferroni post-hoc test. Error bars in graphs represent standard error. For microarray results analysis, RMA normalization was implemented using the Affymetrix Expression Console (v1.3.0.187). Differential expression analysis was performed using R (v3.0.1) and the limma package from Bioconductor. The fold-changes were calculated for the CR subgroup with respect to the *ad libitum* subgroup. A positive fold-change implied that the gene expression was upregulated in the CR mice with respect to the *ad libitum* mice. A negative fold-change implied that the gene expression was downregulated in the CR mice vs. *ad libitum* mice. Statistically significant and biologically meaningful probe sets that were differentially expressed between the two subgroups were selected based on an adjusted *p*-value cut-off of 0.05 and using a fold-change greater than 1.5 or smaller than -1.5.

Bioinformatics analysis of the resulting sequencing reads was performed using the Quantitative Insights Into Microbial Ecology (QIIME) pipeline and the Ribosomal Database Project (RDP) classifier to analyse 16S rDNA sequencing ^6,7^. ChimeraSlayer was used to filter trimmed reads for chimeric sequences, and RDP classifier (version 2.10) was used for bacterial taxonomy assignment with a confidence value threshold of 50%, with trimmed reads clustered into operational taxonomic units at the 97% identity level.

**NMR**

Duodenal content and faecal extracts for NMR spectroscopy were prepared by mixing 20-50 mg of samples with 500 μl of phosphate buffer (0.2 M, pH = 7.4) containing 90% D_2_O and 1% (w/v) sodium 3-(trimethylsilyl) propionate (TSP). After vortex mixing, the samples were frozen in liquid nitrogen and thawed and subsequently homogenized with a tissue lyser (QIAGEN, Hilden, Germany) at 20 Hz for 40 s, followed by centrifugation at 10,000g for 10 min at 4 °C. Supernatants were collected, and the remaining pellet was further extracted as described above. Supernatants obtained from two extractions were combined and centrifuged at 10,000g for 10 min at 4 °C. A total of 600 μl of supernatant was transferred into NMR tubes with an outer diameter of 5 mm. All ^1^H NMR spectra were obtained on a Bruker DRX-600-Avance NMR spectrometer (Bruker, Wissembourg, France) operating at 600.13 MHz for ^1^H resonance frequency using an inverse detection 5 mm ^1^H-^13^C-^15^N cryoprobe attached to a cryoplatform (the preamplifier cooling unit). The ^1^H NMR spectra were acquired at 300K using the Carr-Purcell-Meiboom-Gill (CPMG) spin-echo pulse sequence with pre-saturation and a total spin-echo delay (2nτ) of 100 msec. A total of 128 transients were collected into 64,000 data points using a spectral width of 12 ppm, a relaxation delay of 2.5 s, and an acquisition time of 2.28 sec. Metabolites were assigned using previously published data ^8^ and additional two-dimensional NMR experiments on selected samples. Data were analysed by applying an exponential window function with a line broadening of 0.3 Hz prior to Fourier transformation. The resultant spectra were phased, baseline corrected, and calibrated to TSP (δ 0.00) manually using MestRenova (mNova 10.0). The spectra were subsequently imported into MATLAB (R2014a, The MathsWorks inc.). All data were analysed on full-resolution spectra (33,600 data points). The region containing the water resonance (δ 4.6-5.2) was removed, and spectra were normalized to the probabilistic quotient ^9^ and aligned using a previously published function ^10^. Data were mean-centred prior to analysis using orthogonal projection on latent structure-discriminant analysis (O-PLS-DA). The ^1^H NMR data were used as independent variables (X matrix) and regressed against a dummy matrix (Y matrix) indicating the class of samples (ad-lib or CR) ^11^. The O-PLS-derived model was evaluated for goodness of prediction (Q^2^Y value) using 8-fold cross-validation. The reliability of each model was established using a permutation test of the Y vector (1000 permutations) to determine a *p*-value for each Q^2^Y, as previously described ^12^. The final models were fitted with one predictive component and no orthogonal components and parameters are indicated in the figures. To identify metabolites responsible for discrimination between the animal groups, the O-PLS-DA correlation coefficients (r^2^) were calculated for each variable and back-scaled into a spectral domain, so that the shape of the NMR spectra and the sign of the coefficients were preserved ^13^. The weights of the variables were color-coded, according to the square of the O-PLS-DA correlation coefficients. Correlation coefficients extracted from significant models were filtered so that only significant correlations above the threshold defined by Pearson’s critical correlation coefficient (*p* < 0.05; |r^2^| > 0.49) were considered significant. For illustration purposes, the area under the curve for several signals of interest was integrated and significance was tested using one-way ANOVA and Sidak’s multiple comparison post-tests.

**REFERENCES**

1 Evans, E. M. & Burdett, K. The use of isolated cells to assess the contribution of the mucosal epithelium to the metabolism of the intestinal wall. *Gut* **14**, 98-103 (1973).

2 Huang da, W., Sherman, B. T. & Lempicki, R. A. Systematic and integrative analysis of large gene lists using DAVID bioinformatics resources. *Nat Protoc* **4**, 44-57 (2009).

3 Dimmer, E. C. *et al.* The UniProt-GO Annotation database in 2011. *Nucleic Acids Res* **40**, D565-570 (2012).

4 Barger, J. L. *et al.* Identification of tissue-specific transcriptional markers of caloric restriction in the mouse and their use to evaluate caloric restriction mimetics. *Aging Cell* **16**, 750-760 (2017).

5 Selman, C. *et al.* Coordinated multitissue transcriptional and plasma metabonomic profiles following acute caloric restriction in mice. *Physiol Genomics* **27**, 187-200 (2006).

6 Caporaso, J. G. *et al.* QIIME allows analysis of high-throughput community sequencing data. *Nature Methods* **7**, 335-336 (2010).

7 Wang, Q., Garrity, G. M., Tiedje, J. M. & Cole, J. R. Naive Bayesian classifier for rapid assignment of rRNA sequences into the new bacterial taxonomy. *Applied and Environmental Microbiology* **73**, 5261-5267 (2007).

8 Zhao, Y. *et al.* Gut microbiota composition modifies fecal metabolic profiles in mice. *J Proteome Res* **12**, 2987-2999 (2013).

9 Dieterle, F., Ross, A., Schlotterbeck, G. & Senn, H. Probabilistic quotient normalization as robust method to account for dilution of complex biological mixtures. Application in 1H NMR metabonomics. *Anal Chem* **78**, 4281-4290 (2006).

10 Veselkov, K. A. *et al.* Recursive segment-wise peak alignment of biological (1)h NMR spectra for improved metabolic biomarker recovery. *Anal Chem* **81**, 56-66 (2009).

11 Trygg, J. & Wold, S. Orthogonal projections to latent structures (O-PLS). *Journal of Chemometrics* **16**, 119-128 (2002).

12 Eriksson, L., Trygg, J. & Wold, S. CV-ANOVA for significance testing of PLS and OPLS® models. *Journal of Chemometrics* **22**, 594-600 (2008).

13 Cloarec, O. *et al.* Evaluation of the orthogonal projection on latent structure model limitations caused by chemical shift variability and improved visualization of biomarker changes in 1H NMR spectroscopic metabonomic studies. *Anal Chem* **77**, 517-526 (2005).

**Supplementary figure legends**

**Supplementary Figure 1: Experimental setup.** Mice were divided into experimental groups with a minimum of eight animals per group

**Supplementary Figure 2:**  **Body parameters of mice.** Mouse body weight (A), body fat content (B), and the lengths of the small intestine (C) and colon (D) were measured under *ad libitum* conditions and after 14 days of CR. The lengths of the small intestine (E) and colon (F) were corrected for body mass.

**Supplementary Figure 3:** **Analysis of enriched interacting partners of the genes affected by CR and gene expression in mucosa of overnight fasting.** (A) Putative effect of CR on key transcriptional factors and subsequent impact on the gene transcriptional network. (B) Comparison of the genes with significantly enriched interactions with the upregulated or downregulated genes after CR. The gene expression in the duodenal epithelium scrapings of mice fasted overnight (Fast) was determined by RT-qPCR and compared with that of *ad libitum* (Ad lib) mice (C). Two-tailed Student’s t-tests were used to determine statistical significance; n=8, **p*<0.05. All data are presented as the mean±SEM.

**Supplementary Figure 4: Caloric restriction (CR) impacts gene expression in multiple tissues and body weight of rapamycin treated mice.** Venn diagram of the enriched GO terms identified for the upregulated and downregulated probe sets for the following tissues studied by Selman *et al*. ^5^: colon, liver, muscle, and/or hypothalamus. Up: upregulated after CR; Down: downregulated after CR. Background: Affymetrix 430 2 array probe sets (B). Venn diagrams of genes differentially expressed in duodenum and differentially expressed in other tissues for (C) up-regulation and (D) down-regulation after CR. Genes were considered differentially expressed in other tissues if the differential expression were observed in at least two tissues from colon, muscle, liver and hypothalamus. Venn diagrams comparing genes regulated in duodenum and (D) gastocnemius muscle, (E) heart, (F) WAT, (G) immune tissues and (H) multiple tissues based on publication by Barger *et al*. ^4^ Body weight was measured for mice under *ad libitum* feeding, after CR, and after rapamycin treatment (E).

**Supplementary Figure 5:** **Pie chart of the faecal microbiota sequencing results.** Data presented are averages for each group, in percentage abundance for phylum (A), class (B), and family (C). Only taxa for which at least one of the group averages is above 1% are shown; other taxa are labelled as ‘remainder (each<1%)’. Taxa with known order and unassigned family are labelled with the order, followed by (). Unassigned data (including phylum) is labelled ‘unassigned’.

**Supplementary Figure 6: Pie chart of the small intestine microbiota sequencing results.** Charts present composition of small intestine flora on the level of phylum (A), class (B), and family (C). Taxa for which at least one of the group averages is above 1% are shown; other taxa are labelled as ‘<1%’. Taxa with known order and unassigned family are labelled with the order, followed by (). Unassigned data (including phylum) is labelled ‘unassigned’.

**Supplementary Figure 7: Microbiota manipulation and CR impact mice cecum size, faecal metabolites and gene expression.** The cecum size was recorded for mice from all experimental groups (A). Faecal content of the SCFAs: acetate (B), propionate (C), and butyrate (D) derived from ^1^H-NMR spectra from faecal extracts for ad lib, CR, AT (antibiotic treated), and AT-CR mice. (E) Gene transcription was determined by RT-qPCR of duodenal epithelium scrapings of several groups of mice: Ad lib, CR, AT, AT-CR, mice transplanted with the faecal microbiota from *ad libitum* mice (MT-FC), mice transplanted with the faecal microbiota from CR mice (MT-F), mice transplanted with the duodenal microbiota from *ad libitum* mice (MT-DC), and mice transplanted with the duodenal microbiota from CR mice (MT-D). One-way ANOVA and the Bonferroni post-hoc test were used to compare the experimental groups; n=8. The following letters indicate statistically significant differences between the groups: a, *ad libitum* and CR; b, AT and AT-CR; and c, CR and AT-CR. All data are presented as the mean±SEM.

**Supplementary Figure 8: Complementary metabolomic results.** Typical 600 MHz ^1^H-NMR spectra of (A) faecal extracts and (B) duodenal content. Key: 1 – bile acids (mixed); 2 – butyrate; 3 – caprylate; 4 – valine; 5 – leucine; 6 – isoleucine; 7 – propionate; 8 – α-ketoisovalerate; 9 – ethanol; 10 – threonine; 11 – lactate; 12 – unknown; 13 – alanine; 14 – 5-amino-valerate; 15 – putrescine; 16 – acetate; 17 – proline; 18 – N-acetyl groups of proteins; 19 – glutamine; 20 – glutamate; 21 – pyruvate; 22 – succinate; 23 – 3-hydroxyphenylpropionic acid; 24 – aspartate; 25 – dimethylamine; 26 – trimethylamine; 27 – choline; 28 – oligosaccharides; 29 – xylose; 30 – α-glucose; 31 – uracil; 32 – fumarate; 33 – tyrosine; 34 – phenylalanine; 35 – hypoxanthine; 36 – methionine; 37 – creatine; 38 – taurine (conjugated to bile acids); 39 – taurine (free); 40 – inosine. Spikes in ^1^H-NMR spectra from a duodenal content (C, D) sample (black) and the same sample plus a standard solution of cholic acid (C) or cholic acid+taurocholic acid (D). The area under the curve of the ^1^H-NMR spectra was integrated for several signals of interest from the duodenal content (G) or faeces (G, H, J, K). Metabolite data are presented as 10-90 percentile boxplots with means. Groups were compared using one-way ANOVA and Sidak’s multiple comparison post-tests. a: *p*<0.05 compared with ad lib, b: *p*<0.05 compared with ad lib, c: *p*<0.05 compared with AT. Plasma concentrations of BHA (F) and BCAAs (H). Two-tailed Student’s t-tests; *p*<0.05.
